# Readdressing the Ongoing Challenge of Missing Data in Youth Ecological Momentary Assessment Studies: Meta-Analysis Update

**DOI:** 10.2196/65710

**Published:** 2025-04-30

**Authors:** Konstantin Drexl, Vanisha Ralisa, Joëlle Rosselet-Amoussou, Cheng K Wen, Sébastien Urben, Kerstin Jessica Plessen, Jennifer Glaus

**Affiliations:** 1 Division of Child and Adolescent Psychiatry Department of Psychiatry Lausanne University Hospital and University of Lausanne Lausanne Switzerland; 2 Medical Library-Cery Department of Psychiatry Lausanne University Hospital and University of Lausanne Prilly Switzerland; 3 Dornsife Center for Self-Report Science College of Letters, Arts,. and Sciences University of Southern California Los Angeles United States

**Keywords:** youth, adolescents, children, ecological momentary assessment, experience sampling methodology, ambulatory assessment, missing data, dropout, meta-analysis, mobile devices

## Abstract

**Background:**

Ecological momentary assessment (EMA) is pivotal in longitudinal health research in youth, but potential bias associated with nonparticipation, omitted reports, or dropout threatens its clinical validity. Previous meta-analytic evidence is inconsistent regarding specific determinants of missing data.

**Objective:**

This meta-analysis aimed to update and expand upon previous research by examining key participation metrics—acceptance, compliance, and retention—in youth EMA studies. In addition, it sought to identify potential moderators among sample and design characteristics, with the goal of better understanding and mitigating the impact of missing data.

**Methods:**

We used a bibliographic database search to identify EMA studies involving children and adolescents published from 2001 to November 2023. Eligible studies used mobile-delivered EMA protocols in samples with an average age up to 18 years. We conducted separate meta-analyses for acceptance, compliance, and retention rates, and performed meta-regressions to address sample and design characteristics. Furthermore, we extracted and pooled sample-level effect sizes related to correlates of response compliance. Risk of publication bias was assessed using funnel plots, regression tests, and sensitivity analyses targeting inflated compliance rates.

**Results:**

We identified 285 samples, including 17,441 participants aged 5 to 17.96 years (mean age 14.22, SD 2.24 years; mean percentage of female participants 55.7%). Pooled estimates were 67.27% (*k*=88, 95% CI 62.39-71.96) for acceptance, 71.97% (*k*=216, 95% CI 69.83-74.11) for compliance, and 96.57% (*k*=169, 95% CI 95.42-97.56) for retention. Despite overall poor moderation of participation metrics, acceptance rates decreased as the number of EMA items increased (log-transformed b=−0.115, SE 0.036; 95% CI −0.185 to −0.045; *P*=.001; *R*^2^=19.98), compliance rates declined by 0.8% per year of publication (SE 0.25, 95% CI −1.3 to −0.3; *P*=.002; *R*^2^=4.17), and retention rates dropped with increasing study duration (log-transformed b=−0.061, SE 0.015; 95% CI −0.091 to 0.032; *P*<.001; *R*^2^=10.06). The benefits of monetary incentives on response compliance diminished as the proportion of female participants increased (b=−0.002, SE 0.001; 95% CI −0.003 to −0.001; *P*=.003; *R*^2^=9.47). Within-sample analyses showed a small but significant effect indicating higher compliance in girls compared to boys (*k*=25; g=0.18; 95% CI 0.06-0.31; *P*=.003), but no significant age-related effects were found (*k*=14; *z* score=0.05; 95% CI −0.01 to 0.16).

**Conclusions:**

Despite a 5-fold increase in included effect sizes compared to the initial review, the variability in rates of missing data that one can expect based on specific sample and design characteristics remains substantial. The inconsistency in identifying robust moderators highlights the need for greater attention to missing data and its impact on study results. To eradicate any health-related bias in EMA studies, researchers should collectively increase transparent reporting practices, intensify primary methodological research, and involve participants’ perspectives on missing data.

**Trial Registration:**

PROSPERO CRD42022376948; https://www.crd.york.ac.uk/PROSPERO/view/CRD42022376948

## Introduction

### Background

Contemporary longitudinal health research focusing on youth increasingly uses the collection of self-reports, which are prompted multiple times per day as participants navigate their daily lives. These techniques are collectively known as ecological momentary assessment (EMA), the experience sampling methodology (ESM), and ambulatory assessment (AA), the latter also encompassing data passively collected via sensors [1]. Initially developed as an ecologically valid instrument to study adolescent development and well-being [2], EMA has evolved into a tool for approaching real-time processes by recording momentary snapshots of participants’ daily real-world environments. Today, it is considered as the method of choice for state-of-the-art multivariate modeling frameworks investigating health behaviors [3] and psychopathology [4,5], with affect dynamics being a major area of innovation [6]. The success of EMA in health research stems from its ability to meet the demands of personalized precision medicine, which requires large amounts of data per person and scalable data collection methods [7]. Despite its widely recognized benefits in capturing subjective time-series data as participants go about their daily lives, the technical requirement for young participants to complete multiple self-evaluations throughout the day poses significant challenges.

### The Importance of Missing Data in EMA Research

One of the critical challenges in EMA studies is to achieve high acceptance, compliance, and retention rates. The acceptance rate, also known as the participation or enrollment rate, represents the percentage of approached individuals who consent to enroll in the study. Throughout this paper, we use the term *acceptance rate* because this term was initially introduced in the discussion of an earlier meta-analysis on missing data in EMA research [8]. Response compliance, defined as the proportion of completed self-evaluations relative to the maximum possible within the study protocol, is expressed as a percentage and represents the inverse of missing data on the prompt level [8,9]. Retention, the opposite of dropout, denotes the percentage of participants who remain engaged throughout the study duration, regardless of their inclusion in subsequent analysis.

Together, these participation metrics capture different forms of missing data at various stages of a study protocol. Nonacceptance (ie, refusing to enroll when invited) results in the absence of an entire time series for a given individual. Compliance rates, which are often reported as sample averages, reflect the proportion of data collected over time from participants who do enroll. Failed retention (ie, dropout) leads to an unplanned truncation of the time series.

When researchers focus solely on data quantity, they may opt to oversample, if unrecruited participants (nonacceptance), missing momentary measures (low compliance), or truncated time series (dropout) do not contain systematically different information. However, if any of these forms of missingness is tied to the underlying phenomena of interest (eg, stress reactivity), these metrics can signal substantial risks to a study’s validity by introducing self-selection bias. This bias may occur when only certain types of participants, perhaps those more resilient or less burdened by the demands of daily assessments, enroll and remain engaged [10]. Consequently, the observed data may no longer represent the intended spectrum of real-life experiences and obscure the underlying phenomena of interest [11], compromising the validity of the research findings.

However, researchers can still investigate clinically relevant selection bias in cases of noncompliance and dropout through missing data analyses, for instance by associating baseline symptom severity with dropout or by correlating compliance rates with the clinical constructs of interest [12]. If refusal to participate occurs before any screening, missing data analysis becomes more complex [13], making nonacceptance the most obscure form of unobserved data in practice.

Furthermore, setting up an EMA study requires balancing the need for sufficient data points with the practicalities and burden of the method [14]. Therefore, understanding how different aspects of the EMA design and participant characteristics influence participation metrics is crucial. By leveraging this knowledge, researchers can tailor EMA studies to enhance acceptability and engagement among youth, thereby mitigating risks of selection bias and enhancing the integrity of the research findings.

### Prior Meta-Analytic Findings

The first meta-analysis on missing data in EMA youth research was conducted by Wen et al [15], focusing on EMA delivered via mobile devices. Their systematic literature search and selection process identified 42 studies published up to March 2016. From these, 36 studies reported compliance rates, yielding a pooled estimate of 78.3%. Surprisingly, the tested design factors (ie, study duration, assessment frequency, adjunctive use of wearables, and incentivization strategy) and participant characteristics (ie, age, gender, and clinical status) did not significantly moderate the overall compliance rate [15]. However, subgroup analyses indicated that higher assessment frequencies led to higher compliance rates in clinical groups; the opposite was true for nonclinical groups, but no such effect was observed for study length.

Researchers conducted further meta-analyses and pooled sample studies, synthesizing data from several hundred studies, either in adult populations [8,16] or without age restrictions (ie, age-inclusive) [17-20]. These studies reported similar overall compliance estimates and noted substantial heterogeneity between studies. Such findings on the impact of design factors as the number of EMA items, sampling schedule, and study duration on response rates have been mixed. While individual studies have found that planning more days with EMA [17], higher prompt frequency [8], and longer surveys [20] correlated with lower compliance rates, these effects have not been consistently replicated in other studies [16,18,20,21]. Similarly, sample characteristics, such as age [20], gender [8], and clinical status [18] showed significant moderation of compliance rates in individual meta-analyses but not across multiple studies. Moreover, 2 meta-analyses found no significant moderators among sample characteristics [17,19]. Financial incentives emerged as a relatively consistent moderator, showing benefits for compliance in 3 meta-analyses [17,19,20], although 2 others found no effect [16,18].

Retention rates have been synthesized in fewer meta-analyses [8,17,19], noting lower retention for psychotic samples [8] and higher retention in intervention studies and those with compliance-contingent incentivization [17]. However, the largest meta-analysis so far found no evidence for any candidate moderator [19].

The existing synthesis of evidence holds limited relevance for contemporary EMA research in youth. Much of the data derives from adult studies, the findings of which may not translate directly to youth populations due to developmental differences in motivational systems and responses to incentives [22-24]. Cross-disciplinary research suggests that youth’s motivation and engagement benefit from playful visual design and gamification [25-27], as well as from parental involvement [28]. However, these design enhancements have not yet been subject to meta-analytic synthesis. Furthermore, the rapid expansion of EMA research into diverse populations and technical advancements over the past 7 years necessitates an updated review and meta-analysis that incorporate these evolving design strategies.

### This Review and Meta-Analysis Update

We conducted this meta-analysis update following expert consensus guidelines [29] and began with consultations with the first author of the initial review to identify significant extensions. Our objectives were to (1) revise the literature search and inclusion process from the prior meta-analysis in youth populations [15] to search for previously undetected samples; (2) provide a cumulative meta-analytic update of all available participation metrics, including acceptance and retention rates; and (3) test novel moderators, such as the implementation of gamification and parental involvement. The methodological approaches described in subsequent sections were informed by a careful review of prior work [8,15-20] to address generic biases in meta-analyses [30] and biases specific to EMA literature, such as inflated compliance rates.

## Methods

### Registration and Reporting

This meta-analysis update was registered in PROSPERO (CRD42022376948) in 2022. Reporting of this paper adheres to the PRISMA (Preferred Reporting Items for Systematic Reviews and Meta-Analyses) 2020 (Multimedia Appendix 1 contains the PRISMA checklist [31].

### Literature Search

The research strategy combined 2 concepts—youth as the population and EMA as the intervention. The following bibliographic databases were searched by a medical librarian (JR-A): MEDLINE ALL Ovid, Embase, APA PsycInfo Ovid, CINAHL with Full Text EBSCO, Web of Science Core Collection, and the Cochrane Library Wiley. The search strategies were designed to identify studies using EMA or related methodologies in youth populations. Search equations were adapted for each database to retrieve semantic descriptions of EMA data collection that did not necessarily use prominent key terms related to EMA, ESM, and AA (refer to Multimedia Appendix 2 [17-19,32-47] for further details). The databases were searched from 2001 to November 2023. The searches were performed without language restriction. The final search strategies were peer reviewed by another information specialist. Multimedia Appendix 2 provides details regarding the search syntax (field labels, Boolean, and proximity operators), keywords (free text words), and index terms (subject headings) used for each database.

The backward citation search was carried out (JR-A) on a selection of 19 review articles of interest (literature review or systematic reviews), using Citationchaser [48]. In total, 16 reviews [17,32-46] were found with the database search and 3 [18,19,47] others were known to the research team. While these reviews targeted studies explicitly labeled as EMA, ESM, or AA protocols, their scope included often broader populations beyond youth.

To improve the comprehensiveness of our search strategy, we also examined the included studies from 4 compliance-focused meta-analyses within the backward sources [15,17-19]. While some overlap was anticipated due to methodological similarities, the degree of overlap was limited for various reasons, including differing inclusion criteria. Additional hand-screening of review tables and supplementary data was conducted as bibliographic details in large meta-analytic datasets are often omitted from main articles.

### Inclusion and Exclusion Criteria

On the basis of a widely accepted working definition of EMA methodology [49], we included surveys collected more than once per day in the natural environment via remote mobile technology and were actively responded to by participants. Eligibility was further defined by a hierarchical list of 12 criteria defining the type of research, sampling characteristics, longitudinal design parameters, the setting, and the modalities of data collection. All eligibility criteria were clarified regarding indications of inclusion versus exclusion (Textbox 1).

Of note, we accepted publications in any language based on the multilingual composition of the review team and the availability of translation software. Furthermore, study quality was not considered as an a priori criteria of eligibility because there is no well-established consensus on specific criteria of quality in this data collection framework. However, the exclusion criteria in this study limit excessive heterogeneity in data collection methodologies. Furthermore, some design characteristics were excluded that are known to be prone to bias, such as the risk of recall bias that arises in paper-pencil formats of EMA that allow backfilling of missed responses. Compared to the initial review [15], we adopted a more inclusive definition of youth [50] and included samples with an average age of 18 years. Blinded pairs of raters (KD and students) assessed eligibility based on titles, abstracts, and full texts through incremental working packages, resolving disagreements with one supervisor (ie, either JG or SU).

**Inclusion and exclusion criteria.**

**Inclusion criteria**

- Type of publication: report of primary empirical research
- Sample characteristics: human participants; samples of multiple participants; youth (ie, mean age ≤18 years)
- Longitudinal design: time-series data (ie, consecutive assessments across multiple consecutive days); momentary assessments (ie, prompt frequency ≥1 per d on average)
- Setting of data collection: free-living environment (eg, unrestricted daily life and routine clinical settings)
- Modalities of data collection: active-subjective self-reports (adjunctive use of sensors allowed); first-person self-report by youth; automated ecological momentary assessment delivery (eg, prompting tactile surveys and speaking interview bot); structured format (ie, repetition of a fixed catalog of items); digitized mobile data capture

**Exclusion criteria**

- Type of publication: other article types (eg, reviews, comments, letters to the editors, and conference abstracts)
- Sample characteristics: samples of nonhuman origin, for example, animals; single-case studies; adults (ie, mean age >18 years)
- Longitudinal design: no time series (eg, cross-sectional, pre-post, and panel designs); low-frequent designs (eg, daily-diary or weekly designs)
- Setting of data collection: simulated environments (ie, experimental control of events and environment, eg, Trier-Stress-Test)
- Modalities of data collection: exclusive use of passive-objective data (eg, wearable sensors and phone use logs); parent report or other-person-report; personal phone interviews; free formats (eg, personal diaries) or permanent free choice among formats (eg, item response, free-format report, or sensor-based sampling); paper-pencil format

### Data Extraction

To identify unique samples from multiple publications, we detected coauthorship networks which we disentangled by progressively matching information on study identifiers (eg, funding and institutional review board numbers), country, year, and sample size. Studies with distinct groups and sufficient information on participant flow or response compliance were extracted at the group level (refer to Sample identification in Multimedia Appendix 3 [8,15,18-20,51-56]). We extracted information on (1) sample characteristics (eg, age and clinical status); (2) participant flow; (3) general study design; (4) EMA design (eg, study length and prompt frequency); (5) EMA questionnaires; (6) technical implementation of EMA; (7) compliance rates and correlates thereof; and (8) incentivization strategies and participant care. To facilitate reliable extraction, we extended the basic operationalizations of acceptance, compliance, and retention rates provided in the Introduction section of this paper to accommodate deviating but compatible reporting practices in the literature. The detailed operationalizations of all variables are provided in the sections Extraction of Included Variables and Harmonization of Reported Compliance Rates in Multimedia Appendix 3. Similar to previous large-scale analyses in the field [16,19], we adapted the extraction process to accommodate the high number of studies and variables to practicable workloads. Following training on 10.1% (26/258) of studies, extractors (KD, VR, and 4 master’s-level students) conducted single extractions reviewed by KD and VR, with modifications logged for regular meetings.

### Risk of Bias

In line with previous reviews [8,19], we did not conduct formal assessments of study quality or risk of bias as per PRISMA guidelines, for two main reasons. First, we considered participation metrics to be minimally affected by publication bias or practices such as p-hacking, because they are rarely the primary outcome of interest. Second, the field lacks a standardized definition of the quality of conducting an EMA study, which cannot be replaced by checklists for reporting an EMA study [32,57]. Thus, we approached assessment of publication bias [58] by visual inspection of funnel plot asymmetry and regression tests with sample size as the predictor [8,59,60]. In addition, we addressed inflated compliance rates from studies reporting compliance only for participants who entered analyses because they met a certain compliance threshold (eg, 70%) [15]. Therefore, we extracted relevant information to quantify the associated weight of effect sizes and to compare inflated versus uninflated compliance rates [16].

### Statistical Analyses

Preprocessing steps involved harmonizing reported information on response compliance as the number of completed over the planned EMA surveys to fractions from 0 to 1 (refer to Harmonization of Compliance Metrics in Multimedia Appendix 3). For studies reporting central tendency and spread in quantiles, we estimated the arithmetic mean and the SD performing the maximum likelihood approach [51] using the R (R Foundation for Statistical Computing) package *estmeansd* [61]. Subsequently, we imputed missing SDs using a meta-regression model with squared compliance rate as a predictor [8] (refer to Imputation of Missing Sampling Variances in Multimedia Appendix 3). We applied the arcsine transformation to acceptance and retention rates to obtain robust variances and to accommodate samples with 100% values [8] (refer to Statistical Analyses in Multimedia Appendix 3 for respective equations).

We used random-effects models to obtain pooled overall estimates for acceptance, compliance, and retention. The exploration of heterogeneity was conducted via the *Q* test [62]. We identified influential samples by Cook distance larger than the median plus 6 times the IQR of their distribution. Similarly, standardized residuals larger than the 100×(1−0.05/(2×*k*))th percentile of a standard normal distribution indicated outliers [63].

Expecting widespread missingness across reported variables, we conducted separate meta-regressions with single predictors. On the basis of power simulations adapted from previous work [52], we required predictor variables to provide at least 40 effect sizes and at least 10 effect sizes per category, targeting a statistical power of 80% for realistic effect sizes at an α level restricted to 0.005 for multiple testing (refer to Power Analyses for Meta-Regressions in Multimedia Appendix 3 for further details). Where appropriate, we combined 2 or more categories to allow for examination of candidate predictors (refer to Extraction of Included Variables in Multimedia Appendix 3). The categorical and continuous predictors included in this study are provided in Textbox 2.

We performed logarithmic or exponential transformation of continuous predictors with excessive positive or negative skew above 3.0, respectively. Additional meta-regressions with multiple predictors tested interactions between major participant characteristics (ie, age, gender, and clinical status) and parameters of protocol intensity (ie, number of EMA days, prompt frequency, and number of items), as well as monetary incentives.

We conducted additional random-effects models on within-study correlates of response compliance and between-person comparisons, if at least 10 effect sizes were available for subsequent evaluation of funnel plot asymmetry [64]. All models were fitted using restricted maximum likelihood estimation techniques with the *metafor* [60] package in R, version 4.3.0 [65]. Statistical code, materials, and datasets are available via the research compendium [66] hosted by the Open Science Framework.

**Categorical and continuous predictors.**

**Categorical predictors**

- clinical status (2 levels; clinical vs healthy)
- diagnostic domains (2 levels; somatic vs psychiatric)
- treatment status (2 levels; none vs pre-, peri-, or posttreatment setting)
- treatment setting (2 levels; inpatients vs outpatients)
- assessment of symptoms (yes or no)
- signaling schedule (2 levels; fixed vs random)
- enhancement of ecological momentary assessment (EMA) material (2 levels; none or not reported vs visual elements or gamification)
- adjunctive passive recordings (yes or no)
- compliance reinforcement (yes or no)
- training of participants in responding to EMA surveys (yes or no)
- participant care (2 levels; none or remote availability vs active strategies)
- parent involvement (4 levels; no parent involvement, providing sporadic assessments, assisting child EMA, and parallel parent EMA)

**Continuous predictors**

- mean sample age (in years)
- percentage of female participants (other sex or gender identities were not considered due to infrequent reporting)
- percentage of White participants
- number of days with EMA
- prompt frequency (ie, number of planned surveys per day)
- response duration
- maximum number of items within a single EMA
- amount of monetary incentivization (in US $)
- year of publication

## Results

### Literature Search and Selection

**Figure 1:**
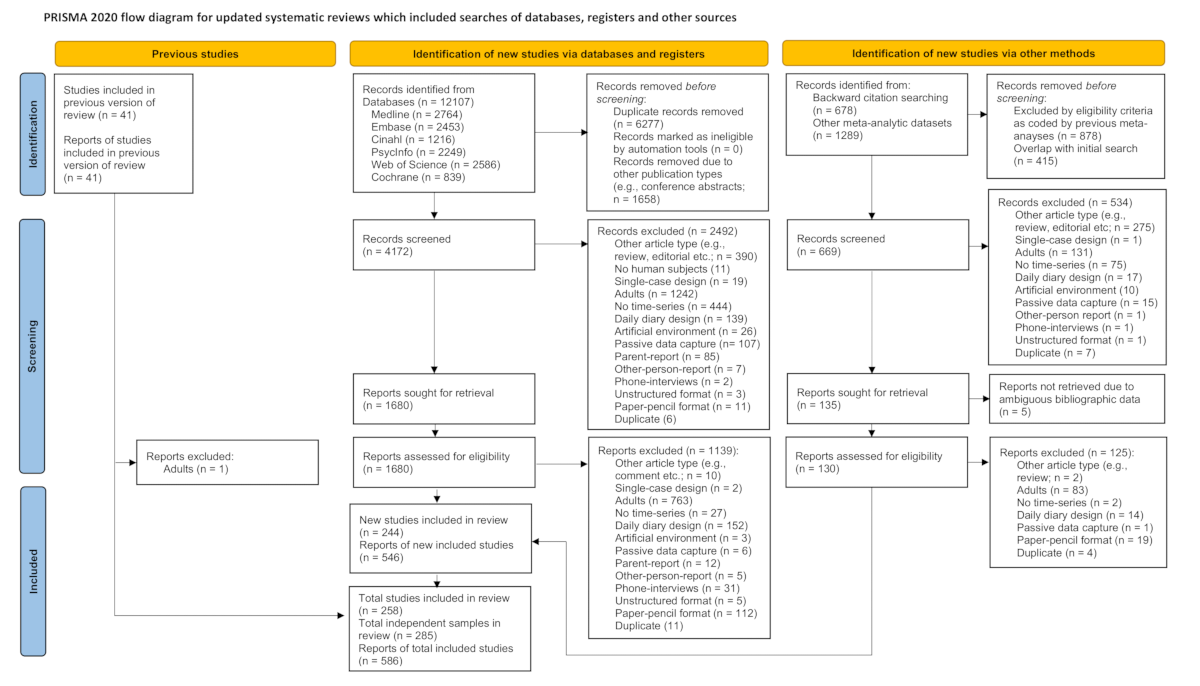
PRISMA (Preferred Reporting Items for Systematic Reviews and Meta-Analyses) 2020 flow diagram. The literature search combined studies selected by the previous edition of the meta-analysis via identification of new studies via databases and registers and identification of new studies via targeted backward chasing of systematic reviews and meta-analyses. Unique studies identified via databases and those identified via other methods do not strictly sum up due to multiple publications on same samples. We adapted the PRISMA 2020 flow diagram [48] by adding further detail on reasons for exclusion at the level of title-abstract screening.

Results of the literature search and selection process are quantified in the extended PRISMA 2020 [67] flow diagram (Figure 1) with regard to all exclusion criteria (Textbox 1). During the initial title-abstract screening of 4172 records, we excluded 2492 (59.7%) records. We were able to obtain all full texts of the remaining 1680 (40.3%) records. Full-text assessment resulted in the exclusion of roughly two-thirds (n=1139, 67.8%) versus one-third considered eligible for extraction (n=541, 32.2%).

The additional search strategy based on targeted backward citation chasing and screening of published meta-analytic datasets led to title-abstract screening of 669 newly identified records. However, 534 (79.8%) records did not qualify for full-text screening and 5 of the remaining 135 (21.2%) could not be retrieved due to ambiguous bibliographic information. Full-text screening excluded the majority (n=125, 96.2%) of these records contributing only 5 newly identified study reports to the overall included 586 study reports (refer to Multimedia Appendix 4 for alphabetical reference list, and Multimedia Appendix 5 [26,63,68-72] for interrater reliability).

Following further deduplication of multiple publications on identical studies, we identified 258 distinct studies covering 285 independent samples [26,33,68-323]. With respect to the eligibility criteria of the initial review, our revised search strategy identified 31 additional samples for the initial review period (ie, 2001 to 2016).

### Descriptive Summary

The selected studies collectively included 17,441 participants (mean percentage of female participants 55.7%; mean percentage of White participants 60.1%), with average ages spanning from 5 to 17.96 years (mean age 14.22, SD 2.24 years; refer to Table 1). Most of the samples were recruited from the United States (169/285, 59.3%). Regarding clinical profiles, 136 (47.7%) samples were recruited from general populations without specific attention to clinical status, 97 (34%) samples met specific diagnostic requirements (eg, via disorder-specific expert interviews [76]), 8 (2.8%) samples consisted of individuals at risk of developing a condition (eg, according to familial incidence [141]), and 29 (10.2%) samples qualified as having no disorders (eg, via extensive diagnostic interviews [115,324]; refer to Table S2 in Multimedia Appendix 5 for complete diagnostic breakdown).

The average prompt frequency was 4.9 per day (SD 5.3 per d, median 4; range 1-57.4) over an average of 18.1 days (SD 28.6 d, median 10; range 1-274). Prompt frequency and number of assessment days showed a negative and statistically significant correlation (*r*=–0.40, 95% CI −0.50 to −0.29; t_256_=−6.94; *P*<.001; Figures S1-S3 in Multimedia Appendix 5). The number of items in the EMA questionnaires was reported for slightly over half the investigated samples (154/285, 54%) among which the individual numbers varied widely, with an average of 19.3 items (SD 23.65, median 15; range 1-248). The presented EMA material involved visual design enhancements or elements of gamification in 38 (13.3%) samples. Monetary compensation was noted in 136 (47.7%) samples with an average amount of US $111.4 (SD US $144.5; median 75, range 5-905). The extracted variables can be explored via an interactive shiny app [325].

**Table 1:** Descriptive statistics of the included studies.

Characteristic			Study level (K=258), k (%)	Sample level (K=285), k (%)
**General characteristics**				
	**Year of earliest publication**			
		2001-2009	14 (5.4)	15 (5.3)
		2010-2019	116 (45)	128 (44.9)
		≥2020	128 (49.6)	142 (49.8)
	**Sample size (n) at enrollment**			
		3-49	64 (24.8)	69 (24.2)
		50-99	36 (14)	39 (13.7)
		≥100	51 (19.8)	53 (18.6)
		Not reported	107 (41.5)	124 (43.5)
**Sample characteristics**				
	**Mean sample age (y)**			
		5-12	67 (26)	74 (26)
		≥13	189 (73.3)	208 (73)
		Not reported	2 (0.8)	3 (1.1)
	**Gender (% girls)**			
		0-49	84 (32.6)	92 (32.3)
		≥50	165 (64)	183 (64.2)
		Not reported	9 (3.5)	10 (3.5)
	**Clinical status**			
		Clinical	83 (32.2)	97 (34)
		At risk	7 (2.7)	8 (2.8)
		Healthy	20 (7.8)	29 (10.2)
		Convenience	124 (48.1)	136 (47.7)
		Mixed	24 (9.3)	15 (5.3)
**Design characteristics**				
	**Number of EMA^a^** **days**			
		1-9	117 (45.3)	131 (46)
		10-28	108 (41.9)	119 (41.8)
		≥29	28 (10.9)	29 (10.2)
		Not reported	5 (1.9)	6 (2.1)
	**Prompt frequency (per d)**			
		1-4	159 (61.6)	172 (60.4)
		≥5	77 (29.8)	87 (30.5)
		Not reported	22 (8.5)	26 (9.1)
	**Sampling scheme**			
		Fixed sampling	92 (35.7)	99 (34.7)
		Random or semirandom sampling	125 (48.4)	140 (49.1)
		Not reported	41 (15.9)	46 (16.1)
	**Number of items**			
		1-19	75 (29.1)	82 (28.8)
		20-39	33 (12.8)	38 (13.3)
		≥40	11 (4.3)	11 (3.9)
		Not reported	139 (53.9)	154 (54)
	**Enhancement of EMA material**			
		Gamification	20 (7.8)	22 (7.7)
		Visual elements	13 (5)	16 (5.6)
		None or not reported	225 (87.2)	247 (86.7)
	**Type of incentivization**			
		Monetary	116 (45)	125 (43.9)
		Nonmonetary	5 (1.9)	5 (1.8)
		Both	11 (4.3)	11 (3.9)
		Not reported	126 (48.8)	144 (50.5)
	**Monetary incentive (US $)**			
		5-99	71 (27.5)	77 (27)
		≥100	50 (19.4)	52 (18.2)
		Not reported	137 (53.1)	156 (54.7)
	**Providing feedback**			
		No or not reported	237 (91.9)	264 (92.6)
		Feedback	21 (8.1)	21 (7.4)
	**EMA training**			
		Individually; face-to-face	101 (39.1)	116 (40.7)
		Grouped sessions	18 (7)	21 (7.4)
		Remotely (print or online)	20 (7.8)	22 (7.7)
		Multiple	4 (1.6)	5 (1.8)
		Not reported	115 (44.6)	121 (42.5)
	**Parent involvement**			
		No involvement	201 (77.9)	222 (77.9)
		Some parent reports	15 (5.8)	18 (6.3)
		Parents assist child EMA	21 (8.1)	23 (8.1)
		Parallel parent EMA	20 (7.8)	22 (7.7)
**Outcome characteristics**				
	**Acceptance rate (%)**			
		21-39	12 (4.7)	13 (4.6)
		40-79	47 (18.2)	54 (18.9)
		≥80	21 (8.1)	21 (7.4)
		Not reported	178 (69)	197 (69.1)
	**Retention rate (%)**			
		34-39	2 (0.8)	2 (0.7)
		40-79	12 (4.7)	12 (4.2)
		≥80	137 (53.1)	147 (51.6)
		Not reported	107 (41.5)	124 (43.5)
	**Mean compliance rate (%)**			
		1-39	9 (3.5)	9 (3.2)
		40-79	121 (46.9)	135 (47.4)
		≥80	78 (30.2)	85 (29.8)
		Not reported	50 (19.4)	56 (19.6)

^a^EMA: ecological momentary assessment.

### Meta-Analyses of Acceptance, Compliance, and Retention Rates

In our meta-analysis, acceptance rates were available for 30.9% (88/285) of samples, compliance rates for 216 (75.8%) samples, and retention rates for 161 (56.5%) samples (refer to Figures S4 and S5 in Multimedia Appendix 5, for detailed patterns of missingness and distributions, respectively). After removal of 1 influential sample [68] that did not relate to the planned moderator analyses (refer to Influential Samples in Multimedia Appendix 5), we noted an average acceptance rate of 67.27% (95% CI 62.39-71.96, 95% prediction interval [PI] 22.99-97.84), an average compliance rate of 71.97% (95% CI 69.83-74.11, 95% PI 41.14-102.8), and an average retention rate of 96.57% (95% CI 95.42-95.42, 95% PI 74.46-100; refer to Figure S7 in Multimedia Appendix 5 for caterpillar plots). *Q* tests indicated that the underlying true effects were heterogeneous (*Ps*<.001). According to fit indexes, the low proportion of samples nested within studies did not support 3-level models (refer to Table S3 in Multimedia Appendix 5). Regression tests for funnel plot asymmetry were statistically significant for acceptance (*P*<.001), and compliance (*P*=.002), but not for retention (*P*=.15; Figure S8 in Multimedia Appendix 5). Visual inspection suggested lack of large sample sizes (ie, high precision) at the upper bounds of participation metrics. However, this asymmetry may be artifactual due to an inherent association of sample size and the difficulty of maintaining engagement in all participants when conducting large-scale projects. The regression test results remained robust when removing 1 sample with an outstanding sample size [322] (*Ps*≤.044).

### Meta-Regression Analyses

**Figure 2:**
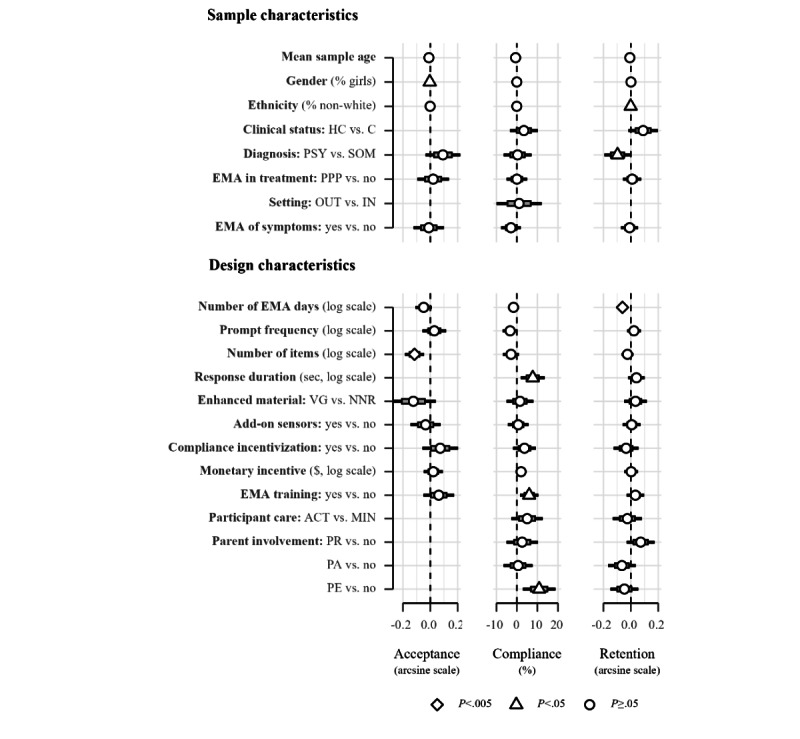
Meta-regression coefficients of sample and design characteristics for participation metrics. Gray and black error bars correspond to the SE and 95% CI, respectively. The dashed line represents the null effect of regression coefficients. Acceptance and retention rates were arcsine transformed before plotting on the natural scale. In addition, moderator variables with skew >3.0 were log-transformed. For binary and categorical predictors, the category expressing absence of the respective sample or design characteristics was used as the reference category. Undepicted estimates were not calculated due to low cell frequencies or overall missingness. ACT: active forms of participant care; CG: clinical groups; EMA: ecological momentary assessment; HC: healthy controls; IN: inpatient; MIN: minimal forms of participant care; NNR: none or not reported; OUT: outpatient; PA: parents assisting their children’s participation; PE: parallel parent ecological momentary assessment; PPP: pre, peri, or posttreatment; PR: sporadic parent reports; PSY: psychiatric disorders; SOM: somatic diseases; VG: visual enhancement or gamification.

Among candidate moderators (refer to Figure 2 and Table S4 in Multimedia Appendix 5), only a higher number of items in EMA surveys robustly predicted lower acceptance rates (41/285, 14.4%; b=−0.115, SE 0.036; 95% CI −0.185 to −0.045; *P*=.001; *R*^2^=19.98; Figure S9 in Multimedia Appendix 5). To illustrate predictions from this log-transformed predictor, doubling the number of items of an EMA survey from 20 to 40 items would predict a 7.91% decline in acceptance from 60.95% (95% CI 54.6-67.11; 95% PI 23.31-92.2) to 53.04% (95% CI 44.29-61.69; 95% PI 16.58-87.69). A negative effect of female participation was not significant at the restricted α level of .005 (*P*=.02). For compliance, response duration (*P*=.009), participant training (*P*=.009), and parallel parent EMA (*P*=.007) showed positive associations with compliance, but these effects did not survive our restricted significance criterion. However, compliance rates declined significantly by 0.8% per year of publication (216/285, 75.8%; SE 0.25; 95% CI −1.3 to −.3; *P*=.002; *R*^2^=4.17; Figure S9 in Multimedia Appendix 5). Retention showed a robust negative association with the number of assessment days (158/285, 55.4%; b=−0.061, SE 0.015; 95% CI −0.091 to −0.032; *P*<.001; *R*^2^=10.06; Figure S9 in Multimedia Appendix 5). Accordingly, doubling the planned number of assessment days from 14 to 28 corresponds to a reduction in retention of approximately 1.76% from 96.02% (95% CI 94.79-97.09; 95% PI 73.72-100) to 94.26% in 28 days (95% CI 92.3-95.94; 95% PI 69.94-100).

We detected no significant difference between samples with reportedly inflated compliance rates and uninflated compliance rates (*P*=.85). Further sensitivity analyses of meta-regressions based exclusively on uninflated rates resulted in a substantial loss of precision, with none of the described effects surviving the restricted α level (refer to Figure S10 in Multimedia Appendix 5). Higher-order meta-regression models revealed that the percentage of female participants and the log-transformed amount of monetary incentives interacted significantly, showing decreasing benefits of monetary incentives with higher female participation (103/285, 36.1%; b=−0.002, SE 0.001; 95% CI −0.003 to −0.001; *P*=.003; *R*^2^=9.47; Table S5 in Multimedia Appendix 5).

### Meta-Analyses of Within-Study Correlates of Compliance

**Figure 3:**
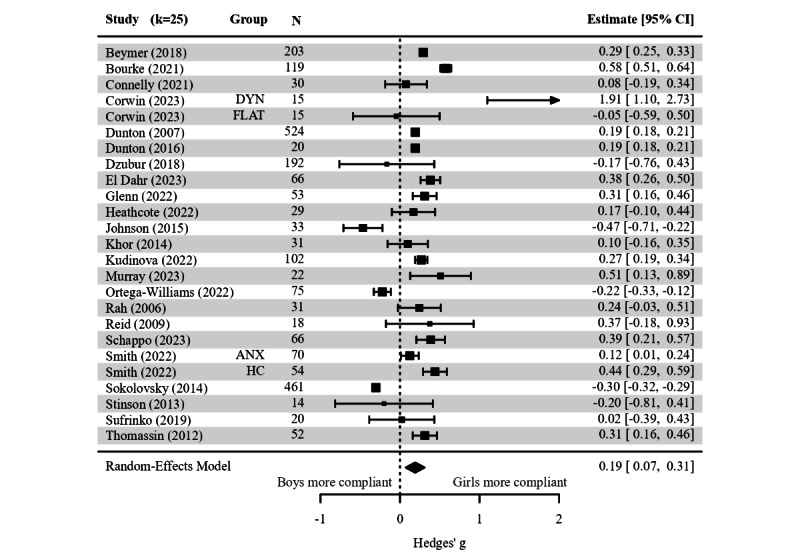
Forest plot of gender differences in response compliance across studies. The size of the square corresponds to the relative weight of the analysis in the meta-analytic random-effects model. Error bars show the 95% CI for each sample estimate. Point estimates below 0 provide support for boys being more compliant than girls, whereas point estimates above 0 provide support for girls being more compliant than boys. The diamond below the sample-specific part of the forest plot depicts the pooled estimate and its width marks the corresponding CI. A pooled effect size is considered significant when the diamond does not cross the dashed line at 0 [79,84,89,91,92,109,127,131,140,151,182,195,201,214,227,239,240, 242,246,261,265,283,289]. ANX: anxiety disorders; DYN: dynamic incentivization scheme; FLAT: flat incentivization scheme; HC: healthy controls.

**Figure 4:**
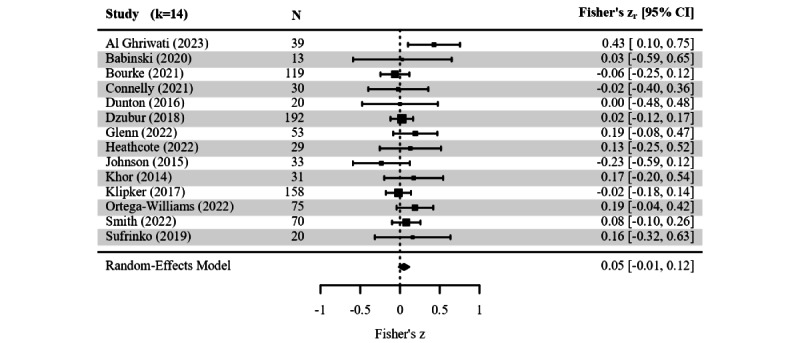
Forest plot of age-compliance correlation coefficients across studies. The size of the square corresponds to the relative weight of the analysis in the meta-analytic random-effects model. Error bars show the 95% CI for each sample estimate. Diverse types of correlation coefficients were harmonized as Fisher’s z scores to facilitate quantitative synthesis. The diamond below the sample-specific part of the forest plot depicts the pooled estimate and its width marks the corresponding 95% CI. A pooled effect size is considered significant when the diamond does not cross the dashed line at 0 [84,92,140,189,195,201,214,227,240,246,265,283,297,302].

The results of missing data analyses regarding sample and design characteristics were available in 52 (20.2%) studies, and 42 (16.3%) provided at least 1 effect size. We performed meta-analysis for differences by gender (25/285, 8.8%), and age (14/285, 4.9%), but not for baseline symptom severity (9/285, 3.2%), ethnicity (5/285, 1.8%), and clinical status (5/285, 1.8%). Contrary to the meta-regression findings, pooled gender differences showed a small significant effect with higher compliance found in girls (*g*=0.18, SE 0.06; 95% CI 0.06-0.31; *P*=.003; Figure 3). The pooled correlation between age and response compliance remained small and did not reach significance (*z* score=0.05, 95% CI −0.01 to 0.16; Figure 4). Neither regression tests nor visual inspection indicated funnel plot asymmetry, and we detected no influential samples (refer to Figure S11 in Multimedia Appendix 5). However, the present pooled effects ignored 23 narrative findings, with 10 and 11 null effects described for gender and age, respectively.

## Discussion

### Principal Findings

Aiming for a complete capture of EMA research conducted in youth, this meta-analysis provides a comprehensive cumulative update marking a 5-fold increase in the total number of included compliance rates compared to the initial review [15]. In addition, it includes the first youth-specific analysis of acceptance and retention and investigates a fine-grained set of sample and design characteristics as potential moderators of missing data.

Our meta-analysis of acceptance rates is also the first of its kind among the wider EMA literature. The negative association between the number of items and acceptance warrants further explanation. At first glance, it may seem puzzling that survey volume would deter individuals who have not yet experienced the burden of completing an EMA. Here, we outline several considerations that might explain this result but require deeper empirical investigation. First, even if the exact number of items was not explicitly disclosed in all studies, potential participants could have inferred from general study descriptions (eg, the time anticipated to respond to one survey), presented training material, or the collaborator’s attitude about the study’s demands that the protocol involved a substantial number of questions. Although we cannot determine these commonly unreported factors of communication using meta-analysis, an experimental study documented the negative effect of described protocol intensity, including the time required to respond to an EMA prompt and on the willingness of adult crowd-workers to participate in hypothetical studies [326]. While this finding substantially supports the assumption that anticipated burden influences the acceptance rate, such experimental research is still lacking in youth. Moreover, we did not observe similar effects for study length and prompt frequency.

Second, the negative relationship between survey volume and acceptance might also reflect broader study characteristics. For instance, the larger-scale studies in our meta-analysis tended to have both more survey items as indicated by the strongest correlation found between the study variables (*r*=.53, refer to Figure S2 in Multimedia Appendix 5) and lower acceptance rates (refer to Figure S8 in Multimedia Appendix 5). This suggests that broader study design strategies beyond item count alone may be at play linking more intense EMA surveys and high outreach of invitations, with less efficient recruitment. For instance, planning an EMA study with long questionnaires and less targeted recruitment can be described as an opportunistic or unselective design strategy. Specifically, large-scale invitations may involve less-engaging formats (eg, flyers vs individual recruitment by clinician). The intention to maximize the number of collected variables and participants without concentrating on specific target phenomena or a specific target population might then come at the cost of low acceptance. However, this does not unavoidably translate into a threat of validity for specific research paradigms, such as epidemiological phenotyping studies that also control representativeness in their recruitment. Unfortunately, only 14.4% (41/285) of the studies included in our meta-analysis reported both the number of items and the acceptance rate, which limited our ability to isolate the direct effect of survey volume from other confounding variables. Future research should more systematically assess how adolescents learn about the scope and intensity of EMA protocols to clarify how perceived burden may deter participation. Until then, researchers can involve youth from the target population as young partners to inquire about their hypothetical acceptance of invitation to enroll across varying numbers of items. Further pilot testing can examine whether shorter, more targeted surveys will even promote data quality, for instance by minimizing careless response behavior.

Pooling all available information on response compliance, the cumulative estimate of the compliance rate decreased by approximately 6% compared to the result of the previous meta-analysis (71.94% vs 78.26%) [15]. This difference mirrors the general downward trend of 0.8% per publication year, which also aligns with the previously reported, yet steeper, yearly decline of 3.1% in health-related adult EMA research [16]. In line with the same adult meta-analysis, we found no indication that reportedly inflated compliance rates were systematically higher than those that were uninflated. Nonetheless, without knowing the respective *raw* compliance rates, we inevitably overestimate the pooled rate. For instance, if the raw compliance rates scored on average 10% below their reported value, their accumulated meta-analytic weight of 21% would pull the pooled estimate down to 69.84%.

Consistent with the initial review [15], sample, design characteristics, and reporting practices did not reduce unexplained heterogeneity among compliance rates, except for the significant interaction of smaller benefits of monetary incentivization with higher female participation. If we assume lower baseline compliance in boys compared to girls, as indicated by our within-sample finding, this interaction may open a door for practical intervention. Researchers may involve youth from the target population as young partners to attenuate this gender gap in compliance by calibrating monetary incentives to reach equal engagement of boys and girls. At the same time, this interpretation is incoherent with the lack of an equivocal main effect of gender at the between-study level.

In contrast, the balance of female and male gender could also act as an outcome of the proposed monetary incentives at recruitment. From this perspective, incentivization plans may be tailored to balance gender proportions in conditions that are less prevalent in boys (eg, nonsuicidal self-injury) or to control for general self-selection bias of youth identifying as male in mental health research [327].

Of note, the conditions of receiving monetary incentives may be of relevance as well. Other authors cautioned for potential negative effects of conditioning financial rewards to tightly to expected compliance rates [19], hypothesizing that this strategy could draw on the motivation to respond to several prompts above the required minimum. Negative reactivity on the incentivization strategy may also interfere with data quality, if participants prioritize providing the required number of responses rather than engaging in active reflection of their current mental states.

At the same time, we caution against overemphasizing this finding which remains limited in explanation of heterogeneity, modest practical effect size, the strong assumption of baseline gender balance in the recruited population, and the lack of interaction with clinical variables. In addition, this finding may be subject to the ecologic fallacy that arises in meta-analysis when summary statistics act as observations [30]. For instance, the varying proportion of females may be confounded by other sample-level characteristics, such as the disorder (eg, anorexia nervosa vs attention-deficit/hyperactivity disorder) that can explain the gender composition as well as sensitivity to external rewards and the resulting response compliance. Conversely, within-study effects overcome this limitation by directly comparing girls’ and boys’ response compliance. However, only 8.8% of all included studies quantified gender differences and potential moderators, such as the incentivization strategy was not even available, as it is usually fixed for all participants within the same study. Nonetheless, the finding of girls being more compliant aligns with previous pooled within-study results in adolescents [9]. Looking ahead, research should explore how incentives and number of items interact with sex and gender diversity in clinical samples to further refine compliance strategies, thereby enhancing study design and ensuring more representative, high-quality data.

This meta-analysis provides the first youth-specific synthesis of retention rates, which were close to full retention (96.57%). The number of assessment days was the only factor associated with lower retention, yet the expected loss of participants over time was surprisingly low, predicting average retention rates above 90% for up to 100 days of EMA collection. While publication bias toward optimal retention rates was not evident in funnel plot asymmetry, we underline that our synthesis of retention rates lacks nearly half of all evidence due to poor reporting of participant flow in the literature. Finally, no interactions of the number of assessment days and clinical variables were found, which suggests that individual clinical burden does not necessarily translate to sensitivity to experienced burden in EMA studies.

### How Do We Deal With Heterogeneity?

In line with previous meta-analyses [8,15-19], substantial heterogeneity in compliance rates persists in the literature and is also present in acceptance and retention rates. Moreover, the robustly significant moderators explain only a limited amount of heterogeneity (highest *R*^2^=20%). Accordingly, we found no indication that nonparticipation, omitted responses, or dropouts are selectively linked to clinical characteristics, though small cell frequencies across diagnostic categories hindered further in-depth analysis of specific diseases [32]. However, this does not imply that participation metrics from EMA research manifest randomly and irrespective of the researchers’ attempts to prevent missing data. On the contrary, the reported prediction intervals, as well as the extensive spread of compliance rates in recent years (refer to Figure S9 in Multimedia Appendix 5), suggest that EMA protocols can successfully minimize loss of data, even in disadvantageous configurations with many EMA items, a high proportion of male participants, and low financial incentives.

The persistence of statistical heterogeneity may also reflect that researchers continuously tackle the barriers of EMA research in the field. For example, a recent study applied EMA in such marginalized minority groups as asylum-seeking children [177], while other EMA protocols were scaled to public implementation [26], where any app-store user could become a participant but would receive only minimal participant care [9,19]. Although such pioneering work may suffer from more missing data, it is not necessarily poorly conducted regarding the valuable insights it offers. Specific implementations of EMA will need to address specific sources of bias (eg, language barriers). We underline that preparing an EMA protocol implies a deliberate process of adapting the recommendations [9,328] to the needs of the target group and, in the best case, involves the latter as young partners to identify systematic challenges in recruitment and data collection [329].

However, this perspective on heterogeneity challenges the interpretation of cumulative meta-analytic estimates as benchmarks for individual data collections, as many citing authors did with the initial meta-analysis in youth [15]. Given that incomplete reporting in journal articles undermines many of the present findings, one may question if the published journal article offers an adequate source of information for meta-analyzing the complex determinants of missing data. As a consequence, future meta-analytic endeavors may consider such alternative data collection methods [330] as purely survey-based inquiry from EMA researchers identified via a systematic literature search [331]. Meta-analytic synthesis may also follow a more selective approach by focusing on effect sizes from experimental primary methods research. However, seminal demonstrations of well-conducted experimental research on important EMA design parameters, such as prompt frequency and number of items, do not yet provide consistent effects and are primarily available from adult student populations [332,333].

To help researchers build adequate expectations for future EMA studies based on this work, we strongly recommend filtering the dataset [66] to studies that relate to the individual design and population of interest. Finally, we provide a web-based shiny app [325] that facilitates this task through an easy-to-use interface.

### Limitations

Several limitations exist in this meta-analysis. We did not search for unpublished studies or trial registries, nor did we contact authors for additional data, which could have filled gaps in the reported information. Our inclusion of diverse EMA study designs may have introduced excessive heterogeneity, affecting the clarity of effect sizes, and we assumed the accuracy of reported data without verification. While conventional approaches to study quality and risk of bias assessment do not apply to this meta-analysis on descriptive participation metrics, a consensual definition of quality in EMA data collection could improve confidence. Finally, our penalty for multiple testing could have obscured genuine effects, particularly interactions.

### Recommendations for Transparent EMA Study Reporting

This meta-analysis is not the first to highlight the unfortunate lack of reporting transparency in EMA study reports [15,16,19]. As transparent research practices continue to gain traction, we would like to remind readers—especially those less familiar with open science—that several EMA-specific reporting guidelines and checklists have been available for years [1,9,32]. Indeed, these guidelines informed much of our own data extraction.

Our large-scale review of the youth EMA literature also revealed that the typical journal article format and word-limit constraints often restrict the level of methodological detail one can feasibly include. However, such limitations need not pose a dead end. Multiple formats allow for full disclosure of methods outside the main text, including preregistrations, registered reports, and supplemental materials. Ideally, authors can preregister or prepare a registered report of their detailed EMA protocols before data collection, following established templates [334]. Similarly, researchers drafting manuscripts (including protocol papers) can consider adding a “detailed EMA protocol” section to the supplemental materials. Existing reporting guidelines [1,9,32] can be directly integrated as tables, with each cell describing design elements rather than pointing to page numbers.

We further encourage authors to provide in-depth missing data analyses, including correlates of all participation metrics. Variables reflecting target phenomena or constructs should be examined for potential associations with missingness at both the participant (eg, baseline depression predicting compliance) and the momentary level (eg, perceived stress predicting next moment missingness). Again, supplemental materials offer an ideal space for these additional analyses. Researchers who have already published their main findings could consider releasing a dedicated paper that focuses on feasibility, data availability, and data quality, with instructive examples available (eg, [262]).

All these formats supported our data extraction process in this meta-analysis.

### Conclusions

Overall, this meta-analysis marks a significant advance in the synthesis of health research using EMA in youth populations. Consistent with previous large-scale meta-analyses, heterogeneity among participation metrics remained largely unaffected by the tested moderators. However, underreporting, especially of within-study correlates of missing data, undermined this investigation. To address the limitations identified in this and other studies, future research should focus on improving reporting transparency, expanding methodological research, and involving young partners to enhance study design and data integrity.

Specifically, the strategic integration of participant perspectives may involve young partners in the identification of research priorities, recruitment strategies, and strategies for preventing missing data. A participatory design process can also tailor monetary and nonmonetary incentives to match participant preferences. Further confidence in the protocol can be gained by conducting a preliminary pilot phase to identify and address missing data patterns, to optimize compliance, and minimize attrition. This participatory approach further allows calibrating specific design parameters, such as the use of gamification or parent involvement. Neither of both emerged as significant (*P*>.06) predictor of participation metrics across studies, though tailoring these factors to the participants’ lived experience may promote data quantity and quality in specific target populations and settings. These measures, paired with transparent reporting practices, will help in reducing missing data and boost the clinical utility of EMA studies in diverse youth populations.

## Appendix

Multimedia Appendix 1 PRISMA 2020 checklist.

Multimedia Appendix 2 Search strategy.

Multimedia Appendix 3 Detailed methods.

Multimedia Appendix 4 Included references.

Multimedia Appendix 5 Detailed results.

## Abbreviations

AA: ambulatory assessment
EMA: ecological momentary assessment
ESM: experience sampling methodology
PRISMA: Preferred Reporting Items for Systematic Reviews and Meta-Analyses

## Data Availability

The datasets generated and analyzed during this study are available in the Open Science Repository [66]. This includes bibliographic data of all screened records, the respective reasons for exclusions, as well as the meta-analytic dataset that was used for statistical analyses.
