# Supplementary material for: Readdressing the Ongoing Challenge of Missing Data in Youth Ecological Momentary Assessment Studies: Meta-Analysis Update
Source: J Med Internet Res. 2025 Apr 30;27:e65710. doi: 10.2196/65710 (PMC12079076; doi:10.2196/65710)
Supplement: Multimedia Appendix 2 [file jmir_v27i1e65710_app2.pdf]

This is a Multimedia Appendix to the article **Readdressing the Ongoing Challenge of Missing Data in Youth Ecological Momentary Assessment Studies: Meta-Analysis Update** published in the Journal of Medical Internet Research. For full copyright and citation information see http://dx.doi.org/10.2196/jmir.65710

Drexl K[a], Ralisa V[a] Rosselet-Amoussou J[b], Wen CK[c1], Urben S[a], Plessen KJ[a], Glaus J[a]

[a]Division of Child and Adolescent Psychiatry, Department of Psychiatry, **Lausanne University Hospital and University of Lausanne**, Lausanne, Switzerland

[b]Medical Library-Cery, **Lausanne University Hospital and University of Lausanne**, Switzerland

[c]Dornsife Center for Self-Report Science, **University of Southern California**, Los Angeles, CA, USA

Detailed Search strategy

Table of contents

Bibliographic database search strategies 2
Additional search strategies 8
References 9

Bibliographic database search strategies

Joëlle Rosselet Amoussou, Medical Library-Cery, Lausanne University Hospital and University of Lausanne, Site de Cery, 1008 Prilly, Switzerland, ORCID 0000-0001-6871-5350

The research strategies were peer reviewed by another information specialist prior to execution.

Embase.com

2453 references found, 10 November 2023

('child'/de OR 'school child'/exp OR 'adolescent'/exp OR 'adolescence'/exp OR 'high school student'/exp OR 'elementary student'/exp OR 'middle school student'/exp OR (child* OR adolescen* OR preadolescen* OR pre-adolescen* OR teen* OR youth* OR "high school" OR "elementary school" OR "elementary student*" OR "middle school"):ab,ti,kw) AND ('ecological momentary assessment'/exp OR 'electronic diary'/exp OR 'experience sampling'/exp OR ('naturalistic inquiry'/exp AND 'daily life activity'/exp) OR ("momentary assess*" OR "ambulatory assess*" OR "experience sampling" OR "intensive longitudinal" OR (electronic NEXT/3 (diary OR diaries)) OR e-diary OR e-diaries OR "mobile diary" OR "mobile diaries" OR "mobile assess*"):ab,ti,kw OR (('mobile phone'/exp OR 'text messaging'/exp OR 'mobile health'/exp OR (smartphone* OR "smart phone*" OR "mobile phone*" OR "cell phone*" OR cellphone* OR "mobile device*" OR "text messag*" OR "mobile health" OR mHealth):ab,ti,kw) AND ('ambulatory monitoring'/exp OR ("Ambulatory monitoring" OR momentary):ab,ti,kw OR (("real time" OR real-world):ab,ti,kw AND ('questionnaire'/exp OR 'self report'/de OR 'self evaluation'/de OR 'diary'/exp OR (measur* OR questionnaire* OR scale* OR self-report* OR "self evaluation" OR diary OR diaries OR assess*):ab,ti,kw)) OR (daily NEAR/3 (measur* OR questionnaire* OR scale* OR evaluation* OR report* OR assess* OR diary OR diaries)):ab,ti,kw))) NOT (([conference abstract]/lim OR [conference paper]/lim) AND [<1966-2019]/py) AND [2001-2023]/py

Medline ALL Ovid

Ovid MEDLINE(R) ALL 1946 to November 09, 2023
limit yr="2001 -Current"
2764 references found, 10 November 2023

(exp Child/ OR Adolescent/ OR (child* OR adolescen* OR preadolescen* OR pre-adolescen* OR teen* OR youth* OR "high school" OR "elementary school" OR "elementary student*" OR "middle school").ab,ti,kf.) AND (Ecological Momentary Assessment/ OR ("momentary assess*" OR "ambulatory assess*" OR "experience sampling" OR "intensive longitudinal" OR (electronic ADJ3 (diary OR diaries)) OR e-diary OR e-diaries OR "mobile diary" OR "mobile diaries" OR "mobile assess*").ab,ti,kf. OR ((exp Cell Phone/ OR (smartphone* OR "smart phone*" OR "mobile phone*" OR "cell phone*" OR cellphone* OR "mobile device*" OR "text messag*" OR "mobile health" OR mHealth).ab,ti,kf.) AND (Monitoring, Ambulatory/ OR ("Ambulatory monitoring" OR momentary).ab,ti,kf. OR (("real time" OR real-world).ab,ti,kf. AND (exp "Surveys and Questionnaires"/ OR diagnostic self evaluation/ OR (measur* OR questionnaire* OR scale* OR self-report* OR "self evaluation" OR diary OR diaries OR assess*).ab,ti,kf.)) OR (daily ADJ3 (measur* OR questionnaire* OR scale* OR evaluation* OR report* OR assess* OR diary OR diaries)).ab,ti,kf.)))

APA PsycInfo Ovid

APA PsycInfo 1806 to October Week 5 2023
limit 1 to yr="2001 -Current"
2249 references found, 10 November 2023

(exp adolescent development/ OR exp childhood development/ OR adolescent attitudes/ OR child attitudes/ OR adolescent psychopathology/ OR child psychopathology/ OR adolescent psychology/ OR child psychology/ OR middle school students/ OR exp elementary school students/ OR junior high school students/ OR (100 OR 160 OR 180 OR 200).ag. OR (child* OR adolescen* OR preadolescen* OR pre-adolescen* OR teen* OR youth* OR "high school" OR "elementary school" OR "elementary student*" OR "middle school").mp.) AND (ecological momentary assessment/ OR mobile assessment/ OR ("momentary assess*" OR "ambulatory assess*" OR "experience sampling" OR "intensive longitudinal" OR (electronic ADJ3 (diary OR diaries)) OR e-diary OR e-diaries OR "mobile diary" OR "mobile diaries" OR "mobile assess*").mp. OR ((exp mobile phones/ OR text messaging/ OR mobile health/ OR (smartphone* OR "smart phone*" OR "mobile phone*" OR "cell phone*" OR cellphone* OR "mobile device*" OR "text messag*" OR "mobile health" OR mHealth).mp.) AND (("Ambulatory monitoring" OR momentary).mp. OR (("real time" OR real-world).mp. AND (exp questionnaires/ OR self-report/ OR self-evaluation/ OR exp rating scales/ OR (measur* OR questionnaire* OR scale* OR self-report* OR "self evaluation" OR diary OR diaries OR assess*).mp.)) OR (daily ADJ3 (measur* OR questionnaire* OR scale* OR evaluation* OR report* OR assess* OR diary OR diaries)).mp.)))

CINAHL with Full Text EBSCO

Date de publication: 20010101-20231231
1216 references found, 10 November 2023

(MH "Child+" OR MH "Adolescence+" OR MH "Students, Elementary" OR MH "Students, High School" OR MH "Students, Middle School" OR TI (child* OR adolescen* OR preadolescen* OR pre-adolescen* OR teen* OR youth* OR "high school" OR "elementary school" OR "elementary student*" OR "middle school") OR AB (child* OR adolescen* OR preadolescen* OR pre-adolescen* OR teen* OR youth* OR "high school" OR "elementary school" OR "elementary student*" OR "middle school")) AND ((MH "Naturalistic Inquiry" AND MH "Activities of Daily Living") OR TI ("momentary assess*" OR "ambulatory assess*" OR "experience sampling" OR "intensive longitudinal" OR (electronic W2 (diary OR diaries)) OR e-diary OR e-diaries OR "mobile diary" OR "mobile diaries" OR "mobile assess*") OR AB ("momentary assess*" OR "ambulatory assess*" OR "experience sampling" OR "intensive longitudinal" OR (electronic W2 (diary OR diaries)) OR e-diary OR e-diaries OR "mobile diary" OR "mobile diaries" OR "mobile assess*") OR ((MH "Cellular Phone" OR MH "Text Messaging" OR MH "Smartphone" OR TI (smartphone* OR "smart phone*" OR "mobile phone*" OR "cell phone*" OR cellphone* OR "mobile device*" OR "text messag*" OR "mobile health" OR mHealth) OR AB (smartphone* OR "smart phone*" OR "mobile phone*" OR "cell phone*" OR cellphone* OR "mobile device*" OR "text messag*" OR "mobile health" OR mHealth)) AND (TI ("Ambulatory monitoring" OR momentary) OR AB ("Ambulatory monitoring" OR momentary) OR ((TI ("real time" OR real-world) OR AB ("real time" OR real-world)) AND (MH "Questionnaires+" OR MH "Scales" OR MH "Diaries" OR MH "Self Assessment" OR TI (measur* OR questionnaire* OR scale* OR self-report* OR "self evaluation" OR diary OR diaries OR assess*) OR AB (measur* OR questionnaire* OR scale* OR self-report* OR "self evaluation" OR diary OR diaries OR assess*))) OR TI (daily N2 (measur* OR questionnaire* OR scale* OR evaluation* OR report* OR assess* OR diary OR diaries)) OR AB (daily N2 (measur* OR questionnaire* OR scale* OR evaluation* OR report* OR assess* OR diary OR diaries)))))

Cochrane Database of Systematic Reviews Wiley

Issue 11 of 12, November 2023
Publication Year from 2001 to 2023
1 references found, 10 November 2023

((child* OR adolescen* OR preadolescen* OR pre-adolescen* OR teen* OR youth* OR "high school" OR "elementary school" OR (elementary NEXT/1 student*) OR "middle school"):ab,ti,kw) AND (((momentary NEXT assess*) OR (ambulatory NEXT/1 assess*) OR "experience sampling" OR "intensive longitudinal" OR (electronic NEXT/3 (diary OR diaries)) OR e-diary OR e-diaries OR "mobile diary" OR "mobile diaries" OR (mobile NEXT/1 assess*)):ab,ti,kw OR (((smartphone* OR (smart NEXT/1 phone*) OR (mobile NEXT/1 phone*) OR (cell NEXT/1 phone*) OR cellphone* OR (mobile NEXT/1 device*) OR (text NEXT/1 messag*) OR "mobile health" OR mHealth):ab,ti,kw) AND (("Ambulatory monitoring" OR momentary):ab,ti,kw OR (("real time" OR real-world):ab,ti,kw AND ((measur* OR questionnaire* OR scale* OR self-report* OR (self NEXT/1 evaluation*) OR diary OR diaries OR assess*):ab,ti,kw)) OR (daily NEAR/3 (measur* OR questionnaire* OR scale* OR evaluation* OR report* OR assess* OR diary OR diaries)):ab,ti,kw)))

Cochrane Central Register of Controlled Trials Wiley

Issue 10 of 12, October 2023
Publication Year from 2001 to 2023
838 references found, 10 November 2023

((child* OR adolescen* OR preadolescen* OR pre-adolescen* OR teen* OR youth* OR "high school" OR "elementary school" OR (elementary NEXT/1 student*) OR "middle school"):ab,ti,kw) AND (((momentary NEXT assess*) OR (ambulatory NEXT/1 assess*) OR "experience sampling" OR "intensive longitudinal" OR (electronic NEXT/3 (diary OR diaries)) OR e-diary OR e-diaries OR "mobile diary" OR "mobile diaries" OR (mobile NEXT/1 assess*)):ab,ti,kw OR (((smartphone* OR (smart NEXT/1 phone*) OR (mobile NEXT/1 phone*) OR (cell NEXT/1 phone*) OR cellphone* OR (mobile NEXT/1 device*) OR (text NEXT/1 messag*) OR "mobile health" OR mHealth):ab,ti,kw) AND (("Ambulatory monitoring" OR momentary):ab,ti,kw OR (("real time" OR real-world):ab,ti,kw AND ((measur* OR questionnaire* OR scale* OR self-report* OR (self NEXT/1 evaluation*) OR diary OR diaries OR assess*):ab,ti,kw)) OR (daily NEAR/3 (measur* OR questionnaire* OR scale* OR evaluation* OR report* OR assess* OR diary OR diaries)):ab,ti,kw)))

Web of Science Core Collection

Science Citation Index Expanded (1900-present), Social Sciences Citation Index (1900-present), Arts & Humanities Citation Index (1975-present), Conference Proceedings Citation Index-Science (1990-present), Book Citation Index (2005-present), Emerging Sources Citation Index (2005-present), Current Chemical Reactions and Index Chemicus

Advanced search > More options > Exact search
2586 references found, 10 November 2023

TS=(((child* OR adolescen* OR preadolescen* OR pre-adolescen* OR teen* OR youth* OR "high school" OR "elementary school" OR "elementary student*" OR "middle school")) AND (("momentary assess*" OR "ambulatory assess*" OR "experience sampling" OR "intensive longitudinal" OR (electronic NEAR/2 (diary OR diaries)) OR e-diary OR e-diaries OR "mobile diary" OR "mobile diaries" OR "mobile assess*") OR (((smartphone* OR "smart phone*" OR "mobile phone*" OR "cell phone*" OR cellphone* OR "mobile device*" OR "text messag*" OR "mobile health" OR mHealth)) AND ("Ambulatory monitoring" OR momentary OR (("real time" OR real-world) AND ((measur* OR questionnaire* OR scale* OR self-report* OR "self evaluation" OR diary OR diaries OR assess*))) OR (daily NEAR/2 (measur* OR questionnaire* OR scale* OR evaluation* OR report* OR assess* OR diary OR diaries)))))) AND PY=(2001-2023)

Backward citation search using Citationchaser

Haddaway, N. R., Grainger, M. J., Gray, C. T. (2021) citationchaser: An R package and Shiny app for forward and backward citations chasing in academic searching. doi: 10.5281/zenodo.4543513

See Appendix C, which explains the selection of the 19 articles on which the citation search was carried out.

DOI

10.1007/s11920-018-0913-z
10.1016/j.psychsport.2020.101825
10.1017/S0033291717001659
10.1111/jora.12468
10.1093/jpepsy/jsx078
10.1111/add.14503
10.2196/jmir.4954
10.1016/j.appet.2019.104465
10.1007/s12402-018-0261-1
10.3389/fpsyg.2022.844698
10.1177/10731911211032718
10.1037/emo0000970
10.2196/11967
10.1016/j.jad.2019.11.156
10.1007/s10578-021-01177-8
10.1007/s00787-022-01972-1
10.1038/s41390-023-02918-2
10.2196/jmir.6641
10.1177/10731911211067538

CitationChaser report :

References from your articles (backward citation chasing)
Your 19 articles contained a total of 1,247 references. This corresponds to 1,000 unique IDs. Your RIS file is ready for download and contains 1,000 records exported from Lens.org.
After deduplication with the previous exports > 678 to screen

Additional search strategies

Konstantin Drexl, Division of Child and Adolescent Psychiatry, Department of Psychiatry, Lausanne University Hospital and University of Lausanne, Avenue d'Echallens 9, 1004 Lausanne, Switzerland. konstantin.drexl@chuv.ch https://orcid.org/0000-0001-9023-9629

Given the immense workload associated with citation chasing based on the included set of 285 studies, we decided to conduct a targeted approach focusing on backward citation chasing on review articles with relevant scope. During the phase of title-and-abstract screening of the principal database search, screeners tagged all review articles which were subsequently screened by KD for explicit overlap with the present scope of selected studies. Eligible reviews were required to follow a systematic search strategy for detecting studies labelled as "Ecological Momentary Assessment", "Experience Sampling Methodology", or "Ambulatory Assessment". The aim of this approach was to benefit from the diverse search methods across included reviews. Beyond the method descriptors that were required to be used by the reviews, their specific eligibility criteria may differ from the present review. Specifically, eligible reviews covered but were not restricted to youth age groups. From the 262 tagged systematic and non-systematic review articles, 16 systematic reviews were selected for backward chasing[1–16]. Three more systematic reviews meeting these criteria were added, which were known to the review team but not covered by the principal search strategy[17–19]. Reference data of these 19 articles were obtained, deduplicated and screened for eligibility.

We further cross-checked the extensiveness of our search strategy, by specifically screening the included studies of four reviews[10,16–18] of the backward sources which conducted themselves compliance-focused meta-analyses. Since the planning phase of the present review update, we expected to find a certain overlap in selected studies with these reviews, though the fraction of overlap was expected to be small for various reasons. For instance, Wrzus and Neubauer[18] did not restrict their selection by age and synthesized only a random subset of all eligible studies, obtaining a youth-related subsample of 59 studies, with 29 studies meeting the eligibility criteria of the present review. Hand-screening available review tables and supplementary data was necessary, since bibliographic information of large meta-analytic samples is typically too large for the reference section of the main article. Overall, additional screening of additional sources was based on 1289 references (see PRISMA 2020 diagram [Main article Figure 1]).

References

1. Baltasar-Tello I, Miguelez-Fernandez C, Penuelas-Calvo I, Carballo JJ. Ecological Momentary Assessment and Mood Disorders in Children and Adolescents: a Systematic Review. Curr Psychiatry Rep 08 01;20(8):66. doi: 10.1007/s11920-018-0913-z

2. Bourke M, Hilland TA, Craike M. A systematic review of the within-person association between physical activity and affect in children's and adolescents' daily lives. Psychol Sport Exerc 2021;52:N.PAG-N.PAG. doi: 10.1016/j.psychsport.2020.101825

3. Dubad M, Winsper C, Meyer C, Livanou M, Marwaha S. A systematic review of the psychometric properties, usability and clinical impacts of mobile mood-monitoring applications in young people. Psychol Med 2018 Jan;48(2):208–228. doi: 10.1017/s0033291717001659

4. Duvenage M, Uink BN, Zimmer-Gembeck MJ, Barber BL, Donovan CL, Modecki KL. Ambulatory Assessment of Adolescent Coping: It's a Complicated Process. J Res Adolesc 9AD;29(3):578–594. doi: 10.1111/jora.12468

5. Heron KE, Everhart RS, McHale SM, Smyth JM. Using Mobile-Technology-Based Ecological Momentary Assessment (EMA) Methods With Youth: A Systematic Review and Recommendations. J Pediatr Psychol 11 01;42(10):1087–1107. doi: 10.1093/jpepsy/jsx078

6. Liao Y, Skelton K, Dunton G, Bruening M. A Systematic Review of Methods and Procedures Used in Ecological Momentary Assessments of Diet and Physical Activity Research in Youth: An Adapted STROBE Checklist for Reporting EMA Studies (CREMAS). J Med Internet Res 2016 Jun 21;18(6):e4954. doi: 10.2196/jmir.4954

7. Mason TB, Do B, Wang S, Dunton GF. Ecological momentary assessment of eating and dietary intake behaviors in children and adolescents: A systematic review of the literature. Appetite 01 01;144:104465. doi: 10.1016/j.appet.2019.104465

8. Miguelez-Fernandez C, de Leon SJ, Baltasar-Tello I, Penuelas-Calvo I, Barrigon ML, Capdevila AS, Delgado-Gomez D, Baca-Garcia E, Carballo JJ. Evaluating attention-deficit/hyperactivity disorder using ecological momentary assessment: a systematic review. Atten Deficit Hyperact Disord 2018 Dec;10(4):247–265. doi: 10.1007/s12402-018-0261-1

9. Molsa ME, Lax M, Korhonen J, Gumpel TP, Soderberg P. The Experience Sampling Method in Monitoring Social Interactions Among Children and Adolescents in School: A Systematic Literature Review. Front Psychol 2022;13:844698. doi: 10/gs82tt

10. Ottenstein C, Werner L. Compliance in Ambulatory Assessment Studies: Investigating Study and Sample Characteristics as Predictors. Assessment SAGE Publications Inc; 2022 Dec 1;29(8):1765–1776. doi: 10.1177/10731911211032718

11. Reitsema AM, Jeronimus BF, van Dijk M, de Jonge P. Emotion dynamics in children and adolescents: A meta-analytic and descriptive review. Emotion 2022 Mar;22(2):374–396. doi: 10.1037/emo0000970

12. Romanzini CLP, Romanzini M, Batista MB, Barbosa CCL, Shigaki GB, Dunton G, Mason T, Ronque ERV. Methodology Used in Ecological Momentary Assessment Studies About Sedentary Behavior in Children, Adolescents, and Adults: Systematic Review Using the Checklist for

Reporting Ecological Momentary Assessment Studies. J Med Internet Res 05 15;21(5):e11967. doi: 10.2196/11967

13. Sequeira L, Perrotta S, LaGrassa J, Merikangas K, Kreindler D, Kundur D, Courtney D, Szatmari P, Battaglia M, Strauss J. Mobile and wearable technology for monitoring depressive symptoms in children and adolescents: A scoping review. J Affect Disord 2020 Mar;265:314–324. doi: 10.1016/j.jad.2019.11.156

14. Thunnissen MR, Aan Het Rot M, van den Hoofdakker BJ, Nauta MH. Youth Psychopathology in Daily Life: Systematically Reviewed Characteristics and Potentials of Ecological Momentary Assessment Applications. Child Psychiatry Hum Dev 2021 Jun;01:01. doi: 10.1007/s10578-021-01177-8

15. Urben S, Constanty L, Lepage C, Rosselet Amoussou J, Durussel J, Turri F, Wouters E, Murner-Lavanchy I, Plessen KJ. The added value of a micro-level ecological approach when mapping self-regulatory control processes and externalizing symptoms during adolescence: a systematic review. Eur Child Adolesc Psychiatry 2022 Mar 16;16:16. doi: 10.1007/s00787-022-01972-1

16. Wen CKF, Schneider S, Stone AA, Spruijt-Metz D. Compliance With Mobile Ecological Momentary Assessment Protocols in Children and Adolescents: A Systematic Review and Meta-Analysis. J Med Internet Res 04 26;19(4):e132. doi: 10.2196/jmir.6641

17. Jones A, Remmerswaal D, Verveer I, Robinson E, Franken IHA, Wen CKF, Field M. Compliance with ecological momentary assessment protocols in substance users: a meta-analysis. Addiction 2018/11/22 ed 2019 Apr;114(4):609–619. doi: 10.1111/add.14503

18. Wrzus C, Neubauer AB. Ecological Momentary Assessment: A Meta-Analysis on Designs, Samples, and Compliance Across Research Fields. Assessment 2022/01/13 ed 2022 Jan 11;30(3):825–846. doi: 10.1177/10731911211067538

19. van Dalen M, Snijders A, Dietvorst E, Bracké K, Nijhof SL, Keijsers L, Hillegers MHJ, Legerstee JS. Applications of the experience sampling method (ESM) in paediatric healthcare: a systematic review. Pediatr Res 2023 Dec 7; doi: 10/gs8p7w
