# Supplementary material for: Readdressing the Ongoing Challenge of Missing Data in Youth Ecological Momentary Assessment Studies: Meta-Analysis Update
Source: J Med Internet Res. 2025 Apr 30;27:e65710. doi: 10.2196/65710 (PMC12079076; doi:10.2196/65710)
Supplement: Multimedia Appendix 3 [file jmir_v27i1e65710_app3.pdf]

This is a Multimedia Appendix to the article **Readdressing the Ongoing Challenge of Missing Data in Youth Ecological Momentary Assessment Studies: Meta-Analysis Update** published in the Journal of Medical Internet Research. For full copyright and citation information see http://dx.doi.org/10.2196/jmir.65710

Drexl K[a], Ralisa V[a] Rosselet-Amoussou J[b], Wen CK[c1], Urben S[a], Plessen KJ[a], Glaus J[a]

[a]Division of Child and Adolescent Psychiatry, Department of Psychiatry, **Lausanne University Hospital and University of Lausanne**, Lausanne, Switzerland

[b]Medical Library-Cery, **Lausanne University Hospital and University of Lausanne**, Switzerland

[c]Dornsife Center for Self-Report Science, **University of Southern California**, Los Angeles, CA, USA

Detailed Extraction Methods and Statistical Analyses

Table of contents and figures

Introductory Note 1
Data Extraction 2
Sample Identification 2
Extraction of Included Variables 3
Harmonization of Reported Compliance Rates 7
Statistical Analyses 8
Power Analyses for Meta-Regressions 8
Figure S1. Power curves for meta-regressions 9
Imputation of Missing Sampling Variances of Compliance Rates 11
Figure S2. Imputation of sampling variances for response compliance 13
Arcsine transformation 14
References 15

Introductory Note

While no protocol has been published for the present meta-analysis update, we provide the following details to complement basic information from the method secrtion of the main article.

Data Extraction

Sample Identification

Multiple publications of unique studies can lead to different forms of overlap. When multiple reports described the same data collection, the most comprehensive method description was used for full data extraction, with secondary reports providing complementary information. The primary record identifier for each study (e.g., "Achterhof_2022") was used to label all corresponding reports of the same study (e.g., the "SIGMA" study). In cases of overlapping data collection waves that could not be combined, the largest fraction of data including the start of the EMA protocol was prioritized to ensure sample naivety to the EMA procedures. Multiple groups were considered clustered in the same study if data collections coincided in time and methods. Conversely, reports combining distinct data collections were coded as nested.

Extraction of Included Variables

Data extraction was structured using predefined forms to capture diverse formats of reported information. Preprocessing involved harmonizing different formats and recoding categorical variables to ensure at least 40 non-missing values per variable and at least 10 values per category cell. Below, we describe procedures for selected variables used in the final analyses, with additional details available in the data dictionary (https://osf.io/8nkeu/).

Age

Information on sample age was accepted in any format. If available in quantiles (e.g., Median and Inter-Quartile Range), it was used for maximum-likelihood estimation of the arithmetic mean [1]. For samples with only school year data, plausible age information was drawn from online resources of the respective school system.

Clinical Status

Clinical groups were identified by professional diagnosis, either within the research project according to standard diagnostic systems [2,3]. or by routine clinical diagnosis. At-risk states required objective operationalization, such as clinical scales or familial risk. Healthy nonclinical samples were noted when relevant diseases or disorders were excluded through formal diagnosis or routine clinical evaluation. Convenience samples were either explicitly described as such or coded if none of the prior criteria applied.

Treatment Setting

Data entry initially differentiated between EMA implemented for youth (1) waiting for treatment (pre-treatment), (2) receiving treatment (peri-treatment), and (3) post-treatment. These categories were regrouped into one treatment related category to ensure at least 10 samples per category.

Sampling Scheme

Signal-contingent sampling was indicated when participants received triggering signals that are planned irrespective of the participants' momentary context. Participants are instructed to initiate an EMA survey response upon such triggers. By default, signal-contingent

sampling can co-occur with event-contingent sampling as well as interval contingent sampling. Event-contingent sampling is planned to trigger EMA surveys when specific context factors occur along the assessment period. These can either be detected by the participants themselves according to instructions (e.g., upon every meal or snack) or by an algorithmic decision rule implemented in the EMA platform (e.g., rise in heart-rate in absence of physical activity). Of note, we did not exclude event-based sampling protocols, since under certain setups, response compliance can be operationalized when the event-contingent sampling is clearly defined and quantifiable. For instance, when participants are instructed to self-report medication adherence which, additionally, is monitored via passive sensors [4].

Prompt Frequency

The mean number of planned surveys per day was calculated across all EMA days, accounting for variations such as more prompts on weekends or fewer prompts on school days.

Number of Assessment Days

The total number of assessment days did not include breaks within the EMA protocol.

Number of EMA Items

The extraction of the total number of EMA items required an explicit description of the volume of the complete EMA catalogue. Given the common practice publishing analyses on subsets of administered items, the full EMA catalogue is often not described in single published articles. Consequently, counting only the reported number of *analyzed* items would underestimate the volume of *administered* items. Therefore, we screened all available publications and technical appendices of a given study to obtain the number of items.

Response Duration

We extracted information on the average amount of time participants needed to respond to a single EMA questionnaire. We also accepted approximate values provided in study reports or used the mid-point of reported ranges, if necessary.

Enhanced Material

We sought reported information on EMA questionnaire design and noted use of visual elements such (e.g., pictograms, emojis, pictures, etc.) as well as elements of gamification (e.g., implemented games, avatars, tokens, leader boards, etc.). Corresponding information was either provided explicitly or depicted in the published article. Published questionnaire screens without such elements were indicative for the absence of visual enhancement or gamification. However, the use of visual elements, as well as the absence of visual elements or gamification was rarely indicated, so that we collapsed both enhancement techniques into one. Reports without any information on visual enhancement or gamification were finally treated as lacking these enhancement techniques.

Monetary Incentives

The maximum value of monetary incentives described for each study was recorded, with reported currencies converted to dollars based on OECD exchange rates from 2022[5].

EMA Training

The presence of EMA training was coded when study reports described procedures to explain the EMA data collection to participants before the assessment periods. Training formats included written manuals, videocalls, and in-person training. Absence of EMA training was coded when no such description was provided.

Participant Care

Measures accompanying data collection that involved contact with participants tp troubleshoot technical issues and maintain compliance were recoded into minimally active forms (i.e., instructing participants to reach out, automated messages) and proactive forms (individual calls, individual messages, in-person meetings).

Acceptance Rates

Acceptance rates were only extracted when directly reported or when the number of enrolled (i.e., providing informed consent) and the number of invited people was available (e.g., from the participant flow chart). Exclusion of participants from the EMA protocol after enrollment was not counted against the acceptance rate.

Retention Rates

Participant retention was quantified as the ratio of participants who did not explicitly withdraw from the study after enrollment, even when the EMA protocol had not started for the respective participants. Other reasons for dropout (e.g., clinical stopping rules, technical problems) were not counted against the retention rate. Likewise, post-hoc exclusions of participants from analysis due to specific criteria (e.g., having reported episodes of drug use, meeting a compliance threshold) was also not counted against the retention rate. If the analytical sample size matched the sample size reported for enrollment, minus the beforementioned external reasons for exclusion, we inferred full retention (i.e. 100%) for a given sample.

Compliance Rates

Response compliance was defined as the ratio of entered responses over the number of expected responses according to the EMA protocol and instructions. With the intention of an inclusive working definition for the synthesized compliance rate, we considered a given survey response to be compliant when at least one item of the survey was responded. We also set no restrictions regarding latency between prompt and initiating the response and duration of responding to EMA surveys. Likewise, we did not require the number compliant responses to be controlled for forms of careless responses or other study-specific definitions of invalid responses. In cases where study reports presented more restrictive definitions of response compliance, we tried to extract the total number of any response given. Otherwise, we noted the compliance rate as “not reported”. Finally, protocols that allowed participants to continue EMA monitoring beyond the protocolized length of assessment were not included in the synthesis of compliance rates. For study reports that did not detail the underlying criteria for a valid response to an EMA survey or definition of compliance, we assumed the compliance rate to involve any partial or complete response given within the maximal time window for responding an EMA prompt.

Harmonization of Reported Compliance Rates

Our extraction procedure aimed to include diverse formats of reported compliance rates:

(1) The sample's average percentage compliance rate and its standard deviation (SD).

(2) The overall percentage compliance rate, calculated as the total number of responded EMA surveys divided by the total number of planned surveys.

(3) The sample's average total number of responded EMA surveys and their SD.

(4) The sample's average daily number of responded EMA surveys and their SD.

(5) The sample's average number of compliant study days within the entire protocol, according to the study's definition of a "compliant day."

Values reported in formats (3) to (5) were divided by the number of prompts, prompts per day, and number of days, respectively, to obtain the compliance rate corresponding to

$$\bar{p}_i = \left(\sum_{j=1}^{n_i} p_{ij} / n_i \right)$$

Besides further customary formats were adapted for extraction, for instance, by calculating the average across compliance rates presented separately per time of day.

Statistical Analyses

Power Analyses for Meta-Regressions

Power calculations were not specified before the final selection of eligible samples but used to guide variable selection for the resulting meta-analytic dataset. In absence of a gold-standard approach to power for the present case of conducting multiple meta-regressions on participation metrics, we conducted a series of Power simulations adapted from previous work[6]. Of note, calibration of required sample size and alpha is complicated by the various predictor variables of different types, scales, and number of available samples k, which is a major factor for Power in meta-analytic estimation. The aim of our approach was to define an adequate combination of restrictive α-level and a minimum criterion for k while balancing Type-I and Type-II error rate probabilities for effect sizes that we consider realistic in the context of participation metrics. Specifically, we constructed prototypic scenarios for varying k with parameters inspired from previously published meta-regression results[7,8]. Small k scenarios are especially relevant for categorical predictors with low cell-frequencies (e.g., parental involvement). The simulation setup was fixed on a study sample size of 60 participants per study, average sampling variance of 0.001 per study, and a residual heterogeneity of $\tau^2 = 0.015$. We simulated 1000 meta-analytic datasets of varying number of samples k (i.e., 12, 20, 40, followed by multiples of 40 up to 240). Figure C1 presents the result for prototypical power curves for naïve and penalized α-levels of .05 and .005. The underlying code to reproduce the simulation study is available trough the OSF-repository (https://osf.io/8nkeu/).

Targeting a prototypical categorical predictor with 4 levels, with an 20% compliance increase for one out of four categories (ks = 10, b_0 = 60%), 82.1 % of Monte-Carlo simulations correctly indicated significance at a restricted α = of .005. In a binary predictor scenario, 40 samples with 20 samples per category, Power of .79 was reached for a contrast of 15% response compliance. In a prototypical continuous predictor scenario, we found that an increase of 1% per day of EMA assessment (M = 13; SD = 9; cf. [7]) was covered in 93.9% of Monte-Carlo simulations at an restricted α of .005 (see Figure B1) by 40 samples.

Figure S1. Power curves for meta-regressions

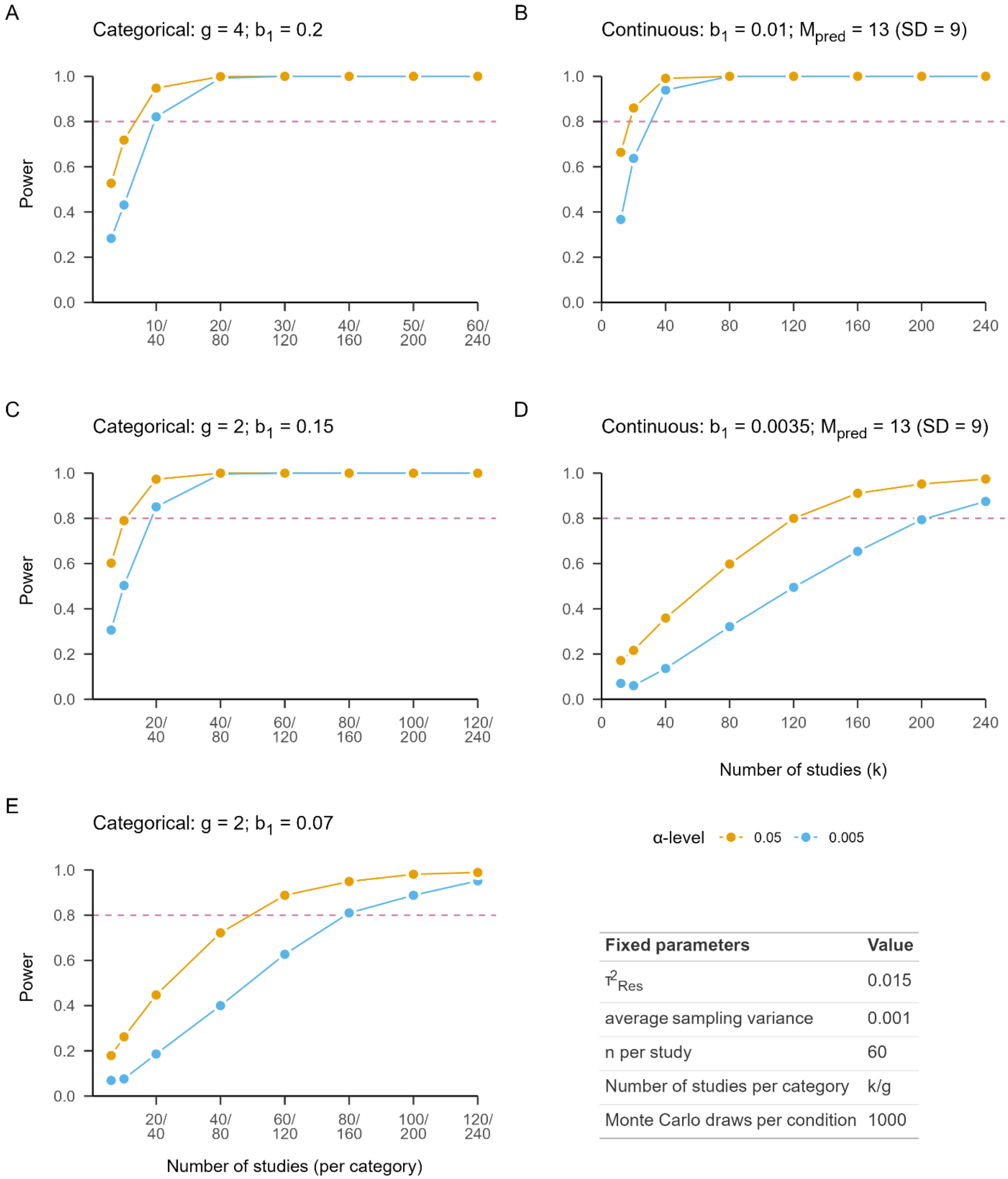

Fixed parameters	Value
τ^2_{Res}	0.015
average sampling variance	0.001
n per study	60
Number of studies per category	k/g
Monte Carlo draws per condition	1000

Power curves for prototypical meta-regression scenarios. Panels **A**, **B**, and **C** show upper-bound effects covered by few studies. Panels show **D** and **E** show lower-bound effects covered by many studies.

Given the poor overall attribution of heterogeneity in participation metrics in published meta-analyses[7–12], we consider the abovementioned effect sizes close to the upper bound of the practically realistic range. Conversely, contrasts above 20% of any participation rate would be surprising with respect to published findings. Hence, the minimum criteria of 40 samples per predictor and 10 per category mark the entry criterion for reliably identifying significant effects in the realistic range. However, high k scenarios allow reliable coverage of regression parameters that are smaller than half of the upper bound. For instance, 200 samples would allow to reach a Power of .794 for a continuous regression parameter of 0.35% per day, and .888 for a binary contrast of 7%.

Imputation of Missing Sampling Variances

To estimate the sampling variance of mean compliance rates $\overline{p_i}$ in each sample, we followed a procedure adapted from a previously published meta-analysis on EMA compliance rates in adults[8]. Let $\overline{p_i}$ represent the mean compliance for the i-th group. The sampling variance $Var[\overline{p_i}]$ is calculated using the formula $Var[\overline{p_i}] = SD_i^2/n_i$, where SD_i denotes the standard deviation of compliance rates among n_i subjects in the i-th group. Consistent with the adult meta-analytic dataset, approximately half of the samples (51.39%) were lacking SD_i values. To address this, we imputed missing SD_i values by leveraging the approximative quadratic relationship between p_i and SD_i. Specifically, SD_i approaches zero when $\overline{p_i}$ is either 0 or 1 and reaches its maximum around $\overline{p_i}$= 0.5.

To perform the imputation, we utilized the following steps:

1. For groups where SD_i was available, we calculated $y_i = \ln[SD_i] + 1/(2(n_i - 1))$, which uses the log-transformed and bias-corrected SD_i value.

2. The approximate sampling variance of y_i is given by $Var[y_i] = 1/(2(n_i - 1))$.

3. We then fitted a random-effects meta-regression model to they_i values, using $\bar{p}_i$ and $\bar{p}_i^2$ as predictors.

The model results are given below:

```
Mixed-Effects Model (k = 105; tau^2 estimator: REML)

tau^2 (estimated amount of residual heterogeneity):     0.3582 (SE = 0.0527)
tau (square root of estimated tau^2 value):             0.5985
I^2 (residual heterogeneity / unaccounted variability): 98.46%
H^2 (unaccounted variability / sampling variability):   65.01
R^2 (amount of heterogeneity accounted for):            8.25%

Test for Residual Heterogeneity:
QE(df = 102) = 7538.5021, p-val < .0001

Test of Moderators (coefficients 2:3):
QM(df = 2) = 11.5070, p-val = 0.0032


Model Results:

              estimate      se     zval    pval    ci.lb    ci.ub
intrcpt        -3.3030  0.9212  -3.5855  0.0003  -5.1085  -1.4975  ***
compl_m         6.2661  2.8840   2.1727  0.0298   0.6136  11.9187    *
I(compl_m^2)   -5.4875  2.1851  -2.5113  0.0120  -9.7703  -1.2047    *
```

Both the linear and the model yielded a significant prediction, though explained variance remained limited (R^2 = 8.25%). Missing y_i values were predicted entering the corresponding p_i values in the model formula. The below figure illustrates both estimation procedures, the quantile-based maximum-likelihood estimation, and the quadratic imputation model for missing SD_i values. The red area shows the 95% Confidence interval of the underlying meta-regression model.

Figure S2. Imputation of sampling variances for response compliance

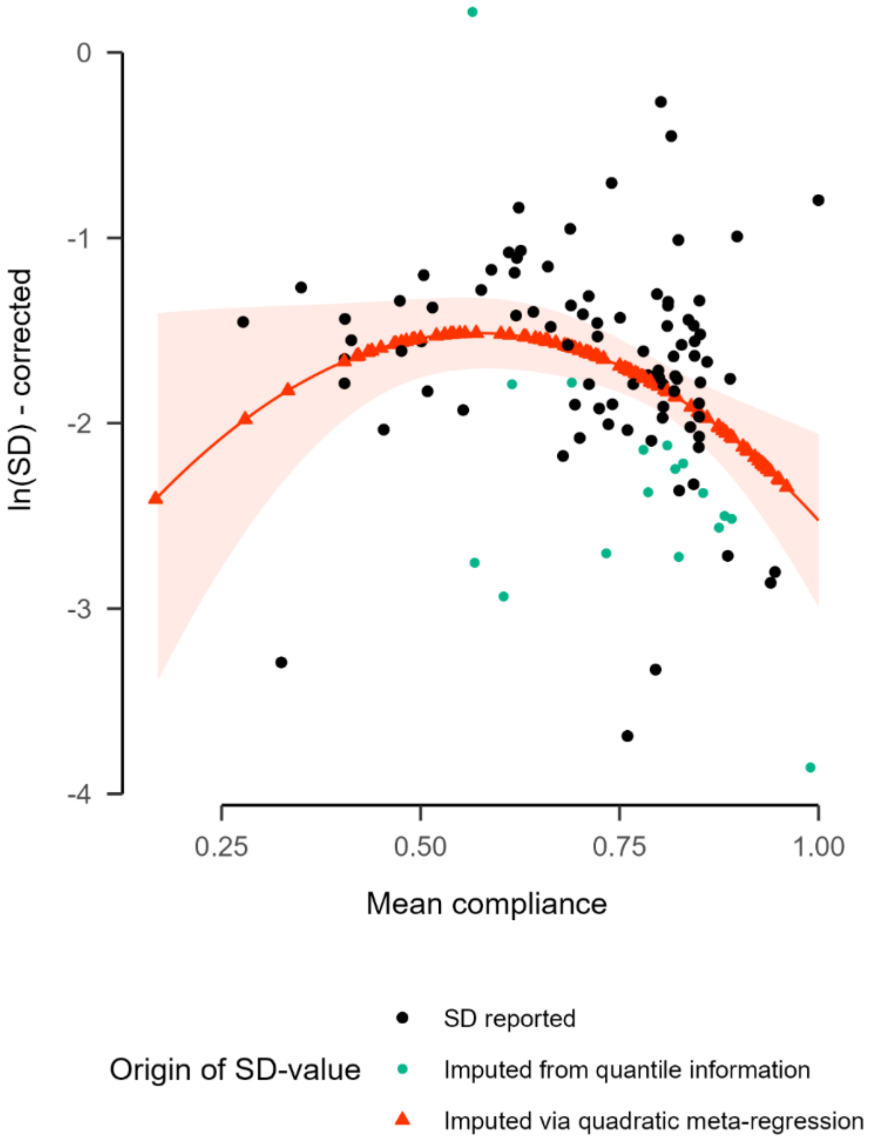

Arcsine Transformation

Retention and compliance rates were arcsine-transformed according to

$$y_i = \arcsin\left(\sqrt{p_i}\right).$$

The corresponding sampling variance were derived from the sample sizes as

$$Var[\bar{y_i}] = 1/(4 * n_i)$$

Notation:

n_i = sample size

y_i = reported acceptance or retention rate

References

1. Cai S, Zhou J, Pan J. Estimating the sample mean and standard deviation from order statistics and sample size in meta-analysis. Stat Methods Med Res SAGE Publications Ltd STM; 2021 Dec 1;30(12):2701–2719. doi: 10.1177/09622802211047348

2. American Psychiatric Association, editor. Diagnostic and statistical manual of mental disorders: DSM-5-TR™. Fifth edition, text revision. Washington, DC: American Psychiatric Association Publishing; 2022. ISBN:978-0-89042-576-3

3. World Health Organization. International Classification of Diseases Eleventh Revision (ICD-11). Geneva, Switzerland: World Health Organization; 2022.

4. Jensen FF, Håkansson KEJ, Overgaard Nielsen B, Weinreich UM, Ulrik CS. Self-reported vs. objectively assessed adherence to inhaled corticosteroids in asthma. Asthma Res Pract 2021 May 31;7(1):7. doi: 10.1186/s40733-021-00072-2

5. OECD. Exchange rates (indicator). doi: 10.1787/037ed317-en

6. Gambarota F, Altoè G. Understanding Meta-Analysis Through Data Simulation With Applications to Power Analysis. Adv Methods Pract Psychol Sci SAGE Publications Inc; 2024 Jan 1;7(1). doi: 10.1177/25152459231209330

7. Wen CKF, Schneider S, Stone AA, Spruijt-Metz D. Compliance With Mobile Ecological Momentary Assessment Protocols in Children and Adolescents: A Systematic Review and Meta-Analysis. J Med Internet Res 04 26;19(4):e132. doi: 10.2196/jmir.6641

8. Vachon H, Viechtbauer W, Rintala A, Myin-Germeys I. Compliance and Retention With the Experience Sampling Method Over the Continuum of Severe Mental Disorders: Meta-Analysis and Recommendations. J Med Internet Res 2019/12/07 ed 2019 Dec 6;21(12):e14475. doi: 10/ggf2m9

9. Ottenstein C, Werner L. Compliance in Ambulatory Assessment Studies: Investigating Study and Sample Characteristics as Predictors. Assessment SAGE Publications Inc; 2022 Dec 1;29(8):1765–1776. doi: 10.1177/10731911211032718

10. Jones A, Remmerswaal D, Verveer I, Robinson E, Franken IHA, Wen CKF, Field M. Compliance with ecological momentary assessment protocols in substance users: a meta-analysis. Addiction 2018/11/22 ed 2019 Apr;114(4):609–619. doi: 10.1111/add.14503

11. Wrzus C, Neubauer AB. Ecological Momentary Assessment: A Meta-Analysis on Designs, Samples, and Compliance Across Research Fields. Assessment 2022/01/13 ed 2022 Jan 11;30(3):825–846. doi: 10.1177/10731911211067538

12. Morren M, van Dulmen S, Ouwerkerk J, Bensing J. Compliance with momentary pain measurement using electronic diaries: A systematic review. Eur J Pain 2009;13(4):354–365. doi: 10.1016/j.ejpain.2008.05.010
