# Supplementary material for: Readdressing the Ongoing Challenge of Missing Data in Youth Ecological Momentary Assessment Studies: Meta-Analysis Update
Source: J Med Internet Res. 2025 Apr 30;27:e65710. doi: 10.2196/65710 (PMC12079076; doi:10.2196/65710)
Supplement: Multimedia Appendix 4 [file jmir_v27i1e65710_app4.pdf]

This is a Multimedia Appendix to the article **Readdressing the Ongoing Challenge of Missing Data in Youth Ecological Momentary Assessment Studies: Meta-Analysis Update** published in the Journal of Medical Internet Research. For full copyright and citation information see http://dx.doi.org/10.2196/jmir.65710

Drexl K[a], Ralisa V[a] Rosselet-Amoussou J[b], Wen CK[c1], Urben S[a], Plessen KJ[a], Glaus J[a]

[a]Division of Child and Adolescent Psychiatry, Department of Psychiatry, **Lausanne University Hospital and University of Lausanne**, Lausanne, Switzerland

[b]Medical Library-Cery, **Lausanne University Hospital and University of Lausanne**, Switzerland

[c]Dornsife Center for Self-Report Science, **University of Southern California**, Los Angeles, CA, USA

Included References

Introductory note

The following reference list comprises all included reports of our meta-analysis update (*k* = 586). We identified multiple publications on 258 identical studies and chose one primary report per study to guide extraction, though all available publications were considered for data extraction. These 258 *primary references* are marked with an asterisk (*). All bibliographic information was directly downloaded from databases with sporadic manual additions (eg, manual retrieval of some DOIs) where required. Machine-readable formats (ie, RIS, bibtex, compressed endnote library), are available via the project's repository on the Open Science Framework with more bibliographic details.

Abadi, M. H., Lipperman-Kreda, S., Shamblen, S. R., Thompson, K., Grube, J. W., Leventhal, A. M., Luseno, W., & Aramburu, C. (2021). The impact of flavored ENDS use among adolescents on daily use occasions and number of puffs, and next day intentions and willingness to vape. Addictive Behaviors, 114, 106773. 10.1016/j.addbeh.2020.106773

Abadi, M. H., Shamblen, S. R., Thompson, K., Lipperman-Kreda, S., Grube, J., Richard, B. O., & Aramburu, C. (2022). Socio-temporal contextual and community factors associated with daily exclusive ENDS use and dual use with tobacco cigarettes among adolescent vapers: an ecological momentary assessment study. BMC Public Health, 22(1), 2289. https://dx.doi.org/10.1186/s12889-022-14787-1

* Aceves, L., Griffin, A. M., Sulkowski, M. L., Martinez, G., Knapp, K. S., Bamaca-Colbert, M. Y., & Cleveland, H. H. (2020). The Affective Lives of Doubled-Up Latinx Youth: Influences of School Experiences, Familism, and Ethnic Identity Exploration. Psychology in the Schools, 57(12), 1878-1895. https://dx.doi.org/10.1002/pits.22391

Achterhof, R., Kirtley, O. J., Schneider, M., Hagemann, N., Hermans, K., Hiekkaranta, A. P., Lecei, A., Decoster, J., Derom, C., De Hert, M., Guloksuz, S., Jacobs, N., Menne-Lothmann, C., Rutten, B. P. F., Thiery, E., van Os, J., van Winkel, R., Wichers, M., & Myin-Germeys, I. (2022). General psychopathology and its social correlates in the daily lives of youth. Journal of Affective Disorders, 309, 428-436. https://dx.doi.org/10.1016/j.jad.2022.04.147

Achterhof, R., Kirtley, O. J., Schneider, M., Hagemann, N., Hermans, K. S. F. M., Hiekkaranta, A. P., Lecei, A., Lafit, G., & Myin-Germeys, I. (2022). Adolescents' real-time social and affective experiences of online and face-to-face interactions. Computers in Human Behavior, 129, N.PAG-N.PAG. 10.1016/j.chb.2021.107159

* Achterhof, R., Kirtley, O. J., Schneider, M., Lafit, G., Hagemann, N., Hermans, K., Hiekkaranta, A. P., Lecei, A., & Myin-Germeys, I. (2021). Daily-Life Social Experiences as a Potential Mediator of the Relationship Between Parenting and Psychopathology in Adolescence. Frontiers in psychiatry Frontiers Research Foundation, 12, 697127. https://dx.doi.org/10.3389/fpsyt.2021.697127

Achterhof, R., Schneider, M., Kirtley, O. J., Wampers, M., Decoster, J., Derom, C., De Hert, M., Guloksuz, S., Jacobs, N., Menne-Lothmann, C., Rutten, B. P. F., Thiery, E., van Os, J., van Winkel, R., Wichers, M., & Myin-Germeys, I. (2022). Be(com)ing social: Daily-life social interactions and parental bonding. Developmental Psychology, 58(4), 792-805. https://dx.doi.org/10.1037/dev0001315

* Akkerman, D. M., Vulperhorst, J. P., & Akkerman, S. F. (2020). A developmental extension to the multidimensional structure of interests. Journal of Educational Psychology, 112(1), 183-203. https://dx.doi.org/10.1037/edu0000361

Akkerman, S. F., & Bakker, A. (2019). Persons pursuing multiple objects of interest in multiple contexts. European Journal of Psychology of Education, 34(1), 1-24. https://dx.doi.org/10.1007/s10212-018-0400-2

* Al Ghriwati, N., Winter, M., Semko, J., Merchant, T. E., & Crabtree, V. M. (2023). The feasibility and acceptability of mobile ecological momentary assessment to evaluate sleep, family functioning, and affect in patients with pediatric craniopharyngioma. Journal of Psychosocial Oncology, 1-16. https://dx.doi.org/10.1080/07347332.2023.2231412

Aldrich, J. T., Lisitsa, E., Chun, S. K., & Mezulis, A. H. (2019). Examining the relationship between daily co-rumination and rumination in response to negative events among adolescents using ecological momentary assessment. Journal of Social and Clinical Psychology, 38(8), 704-719. https://dx.doi.org/10.1521/jscp.2019.38.7.704

* Alfven, G. (2010). SMS pain diary: a method for real-time data capture of recurrent pain in childhood. Acta Paediatrica, 99(7), 1047-1053. https://dx.doi.org/10.1111/j.1651-2227.2010.01735.x

* Algheryafi, R. A., Bevans, K. B., Hiremath, S. V., Lai, J.-S., & Tucker, C. A. (2023). Convergent Validity of the Patient Reported Outcome Measurement Information System-Pediatric Physical Activity Instrument (PROMIS ® -PA) with Wearable Devices in Adolescents. Children, 10(6), 940. 10.3390/children10060940

* Apple, D., & Sequiera, S. (2020). Puberty, parents, and depression: An EMA study in adolescent girls. Psi Chi Journal of Psychological Research, 25(2), 130-141. https://dx.doi.org/10.24839/2325-7342.JN25.2.130

Arbel, R., Mason, T. B., & Dunton, G. F. (2022). Transactional links between children daily emotions and internalizing symptoms: a six-wave ecological momentary assessment study. Journal of Child Psychology and Psychiatry and Allied Disciplines, 63(1), 68-77. https://dx.doi.org/10.1111/jcpp.13432

* Armstrong-Carter, E., Garrett, S. L., Nick, E. A., Prinstein, M. J., & Telzer, E. H. (2023). Momentary links between adolescents' social media use and social experiences and motivations: Individual differences by peer susceptibility. Developmental Psychology, 59(4), 707-719. https://dx.doi.org/10.1037/dev0001503

* Babinski, D. E., & Welkie, J. (2020). Feasibility of Ecological Momentary Assessment of Negative Emotion in Girls With ADHD: A Pilot Study. Psychological Reports, 123(4), 1027-1043. https://dx.doi.org/10.1177/0033294119838757

* Bakshi, N., Smith, M. E., Ross, D., & Krishnamurti, L. (2017). Novel Metrics in the Longitudinal Evaluation of Pain Data in Sickle Cell Disease. Clinical Journal of Pain, 33(6), 517-527. https://dx.doi.org/10.1097/AJP.0000000000000431

* Balkaya-Ince, M., Cheah, C. S. L., Kiang, L., & Tahseen, M. (2020). Exploring daily mediating pathways of religious identity in the associations between maternal religious socialization and Muslim American adolescents' civic engagement. Developmental Psychology, 56(8), 1446-1457. https://dx.doi.org/10.1037/dev0000856

* Balkaya-Ince, M., Tahseen, M., Umarji, O., & Schnitker, S. A. (2023). Does ramadan serve as a naturalistic intervention to promote muslim american adolescents' daily virtues? Evidence from a three wave experience sampling study. The Journal of Positive Psychology, No Pagination Specified. https://dx.doi.org/10.1080/17439760.2023.2169631

Bamps, E., Teixeira, A., Lafit, G., Achterhof, R., Hagemann, N., Hermans, K., Hiekkaranta, A. P., Lecei, A., Kirtley, O. J., & Myin-Germeys, I. (2022). Identifying Clusters of Adolescents Based on Their Daily-Life Social Withdrawal Experience. Journal of Youth & Adolescence, 51(5), 915-926. https://dx.doi.org/10.1007/s10964-021-01558-1

Becker, E. S., Goetz, T., Morger, V., & Ranellucci, J. (2014). The importance of teachers' emotions and instructional behavior for their students' emotions: An experience sampling analysis. Teaching and Teacher Education, 43, 15-26. https://dx.doi.org/10.1016/j.tate.2014.05.002

* Beesdo-Baum, K., Voss, C., Venz, J., Hoyer, J., Berwanger, J., Kische, H., Ollmann, T. M., & Pieper, L. (2020). The Behavior and Mind Health (BeMIND) study: Methods, design and baseline sample characteristics of a cohort study among adolescents and young adults. International Journal of Methods in Psychiatric Research, 29(1), e1804. https://dx.doi.org/10.1002/mpr.1804

Bejarano, C. M., Cushing, C. C., & Crick, C. J. (2019). Does context predict psychological states and activity? An ecological momentary assessment pilot study of adolescents. Psychology of Sport and Exercise, 41, 146-152. 10.1016/j.psychsport.2018.05.008

* Bekman, N. M., Winward, J. L., Lau, L. L., Wagner, C. C., & Brown, S. A. (2013). The impact of adolescent binge drinking and sustained abstinence on affective state. Alcoholism: Clinical & Experimental Research, 37(8), 1432-1439. https://dx.doi.org/10.1111/acer.12096

* Bell, B. M., Alam, R., Mondol, A., Ma, M. Y., Emi, I. A., Preum, S. M., de la Haye, K., Stankovic, J. A., Lach, J., & Spruijt-Metz, D. (2022). Validity and Feasibility of the Monitoring and Modeling Family Eating Dynamics System to Automatically Detect In-field Family Eating Behavior: Observational Study. Jmir Mhealth and Uhealth, 10(2), 23, Article e30211. 10.2196/30211

* Bentley, K. H., Millner, A. J., Bear, A., Follet, L., Fortgang, R. G., Zuromski, K. L., Kleiman, E. M., Coppersmith, D. D. L., Castro-Ramirez, F., Millgram, Y., Haim, A., Bird, S. A., & Nock, M. K. (2023). Intervening on high-risk responses during ecological momentary assessment of suicidal thoughts: Is there an effect on study data? Psychological Assessment, 02, 02. https://dx.doi.org/10.1037/pas0001288

Bernstein, R. A., Smith, A. R., Kitt, E. R., Cardinale, E. M., Harrewijn, A., Abend, R., Michalska, K. J., Pine, D. S., & Kircanski, K. (2023). Threat Appraisal and Pediatric Anxiety: Proof of Concept of a Latent Variable Approach. Clinical Psychological Science, 10. 10.1177/21677026231190349

Beyens, I., Pouwels, J. L., van Driel, II, Keijsers, L., & Valkenburg, P. M. (2021). Social Media Use and Adolescents' Well-Being: Developing a Typology of Person-Specific Effect Patterns. Communication Research, 26, Article 00936502211038196. 10.1177/00936502211038196

Beymer, P. N., Robinson, K. A., Naftzger, N., & Schmidt, J. A. (2022). Program quality, control, value, and emotions in summer stem programs: An examination of control-value theory in an informal learning context. Learning Environments Research, No Pagination Specified. https://dx.doi.org/10.1007/s10984-022-09439-5

* Beymer, P. N., Rosenberg, J. M., Schmidt, J. A., & Naftzger, N. J. (2018). Examining Relationships among Choice, Affect, and Engagement in Summer STEM Programs. Journal of Youth & Adolescence, 47(6), 1178-1191. https://dx.doi.org/10.1007/s10964-018-0814-9

Bickham, D. S., Blood, E. A., Walls, C. E., Shrier, L. A., & Rich, M. (2013). Characteristics of screen media use associated with higher BMI in young adolescents. Pediatrics, 131(5), 935-941. https://dx.doi.org/10.1542/peds.2012-1197

* Bickham, D. S., Hswen, Y., & Rich, M. (2015). Media use and depression: exposure, household rules, and symptoms among young adolescents in the USA. International Journal of Public Health, 60(2), 147-155. https://dx.doi.org/10.1007/s00038-014-0647-6

Bieg, M., Goetz, T., & Hubbard, K. (2013). Can I master it and does it matter? An intraindividual analysis on control-value antecedents of trait and state academic emotions. Learning and Individual Differences, 28, 102-108. https://dx.doi.org/10.1016/j.lindif.2013.09.006

* Bjorling, E. A. (2009). The momentary relationship between stress and headaches in adolescent girls. Headache, 49(8), 1186-1197. https://dx.doi.org/10.1111/j.1526-4610.2009.01406.x

Bjorling, E. A., & Singh, N. (2017). Exploring Temporal Patterns of Stress in Adolescent Girls with Headache. Stress & Health, 33(1), 69-79. https://dx.doi.org/10.1002/smi.2675

Blume, F., Irmer, A., Dirk, J., & Schmiedek, F. (2022). Day-to-day variation in students' academic success: The role of self-regulation, working memory, and achievement goals. Developmental Science, 25(6), e13301. https://dx.doi.org/10.1111/desc.13301

* Boerner, K. E., Desai, U., Luu, J., MacLean, K. E., Munzner, T., Foladare, H., Shen, J., Gill, J., & Oberlander, T. F. (2023). Making Data the Drug: A Pragmatic Pilot Feasibility Randomized Crossover Trial of Data Visualization as an Intervention for Pediatric Chronic Pain. Children, 10(8), 07. https://dx.doi.org/10.3390/children10081355

* Bogaert, L., Van der Gucht, K., Kuppens, P., Kock, M., Schreuder, M. J., Kuyken, W., & Raes, F. (2023). The effect of universal school-based mindfulness on anhedonia and emotional distress and its underlying mechanisms: A cluster randomised controlled trial via experience sampling in secondary schools. Behaviour Research and Therapy, 169, 104405. https://dx.doi.org/10.1016/j.brat.2023.104405

Borgogna, N., Lockhart, G., Grenard, J. L., Barrett, T., Shiffman, S., & Reynolds, K. D. (2015). Ecological momentary assessment of urban adolescents' technology use and cravings for unhealthy snacks and drinks: differences by ethnicity and sex. Journal of the Academy of Nutrition and Dietetics, 115(5), 759-766. https://dx.doi.org/10.1016/j.jand.2014.10.015

Bornas, X., Zuzama, N., Fiol-Veny, A., Roman-Juan, J., & Balle, M. (2021). Diminished complexity of heart rate time series in adolescents facing negative events during everyday life. Revista de Psicologia Clinica con Ninos y Adolescentes, 8(3), 18-25. https://dx.doi.org/10.21134/rpcna.2021.08.3.2

* Borus, J. S., Blood, E., Volkening, L. K., Laffel, L., & Shrier, L. A. (2013). Momentary assessment of social context and glucose monitoring adherence in adolescents with type 1 diabetes. Journal of Adolescent Health, 52(5), 578-583. https://dx.doi.org/10.1016/j.jadohealth.2012.10.003

Bourke, M., Hilland, T. A., & Craike, M. (2021). Daily physical activity and satisfaction with life in adolescents: An ecological momentary assessment study exploring direct associations and the mediating role of core affect. Journal of Happiness Studies: An Interdisciplinary Forum on Subjective Well-Being, No Pagination Specified. https://dx.doi.org/10.1007/s10902-021-00431-z

* Bourke, M., Hilland, T. A., & Craike, M. (2021). Domain specific association between physical activity and affect in adolescents' daily lives: an ecological momentary assessment study. Psychology & Health, 1-20. https://dx.doi.org/10.1080/08870446.2021.1965603

Bourke, M., Patten, R. K., Hilland, T. A., & Craike, M. (2022). Within-Person Associations Between Physical and Social Contexts With Movement Behavior Compositions in Adolescents: An Ecological Momentary Assessment Study Using a Compositional Data Analysis Approach. Journal of Physical Activity & Health, 19(9), 615-622. https://dx.doi.org/10.1123/jpah.2022-0233

Bourke, M., & Phillips, S. M. (2023). Associations between type and timing of sedentary behaviour and affect in adolescents: An ecological momentary assessment study. Mental Health and Physical Activity, 25. 10.1016/j.mhpa.2023.100550

* Brannon, E. E., Cushing, C. C., Crick, C. J., & Mitchell, T. B. (2016). The promise of wearable sensors and ecological momentary assessment measures for dynamical systems modeling in adolescents: a feasibility and acceptability study. Translational Behavioral Medicine, 6(4), 558-565.

Bray, P., Bundy, A. C., Ryan, M. M., & North, K. N. (2010). Feasibility of a computerized method to measure quality of 'everyday' life in children with neuromuscular disorders. Physical and Occupational Therapy in Pediatrics, 30(1), 43-53. 10.3109/01942630903294687

* Bray, P., Bundy, A. C., Ryan, M. M., & North, K. N. (2017). Can in-the-moment diary methods measure health-related quality of life in Duchenne muscular dystrophy? Quality of Life Research, 26(5), 1145-1152. https://dx.doi.org/10.1007/s11136-016-1442-z

Breil, S. M., Schweppe, P. C., Geukes, K., Biesanz, J. C., Quintus, M., Wagner, J., Wrzus, C., Nestler, S., & Back, M. D. (2022). The incremental validity of average states: A replication and extension of Finnigan and Vazire (2018). Journal of Personality and Social Psychology, No Pagination Specified. https://dx.doi.org/10.1037/pspp0000408

Brick, L. A., Gajewski-Nemes, J. A., Marraccini, M. E., Brown, S., Armey, M., & Nugent, N. R. (2023). Ecological Momentary Assessment of Cannabis Use and Affect Among

Adolescents Following Psychiatric Discharge. Journal of Studies on Alcohol & Drugs, 84(1), 67-78.

Broda, M. (2017). Using multilevel models to explore predictors of high school students' nonresponse in experience sampling method (ESM) studies. Social Science Computer Review, 35(6), 733-750. https://dx.doi.org/10.1177/0894439316667049

* Bromberg, M. H., Connelly, M., Anthony, K. K., Gil, K. M., & Schanberg, L. E. (2014). Self-reported pain and disease symptoms persist in juvenile idiopathic arthritis despite treatment advances: an electronic diary study. Arthritis & Rheumatology, 66(2), 462-469. https://dx.doi.org/10.1002/art.38223

Bromberg, M. H., Connelly, M., Anthony, K. K., Gil, K. M., & Schanberg, L. E. (2016). Prospective Mediation Models of Sleep, Pain, and Daily Function in Children With Arthritis Using Ecological Momentary Assessment. Clinical Journal of Pain, 32(6), 471-477. https://dx.doi.org/10.1097/AJP.0000000000000298

* Browning, C. R., Ford, J. L., Tarrence, J., Kertes, D. A., Pickler, R. H., Way, B. M., & Calder, C. A. (2023). Everyday perceptions of safety and racial disparities in hair cortisol concentration. Psychoneuroendocrinology, 153, 106088. https://dx.doi.org/10.1016/j.psyneuen.2023.106088

* Buhr, L., Moschko, T., Eppinger Ruiz de Zarate, A., Schwarz, U., Kuhnhausen, J., & Gawrilow, C. (2022). The Association of Self-Reported ADHD Symptoms and Sleep in Daily Life of a General Population Sample of School Children: An Inter- and Intraindividual Perspective. Brain Sciences, 12(4), 25. https://dx.doi.org/10.3390/brainsci12040440

* Bui, A. A. T., Hosseini, A., Rocchio, R., Jacobs, N., Ross, M. K., Okelo, S., Lurmann, F., Eckel, S., Dzubur, E., Dunton, G., Gilliland, F., Sarrafzadeh, M., & Habre, R. (2020). Biomedical REAl-Time Health Evaluation (BREATHE): Toward an mHealth informatics platform. JAMIA Open, 3(2), 190-200. 10.1093/jamiaopen/ooaa011

Bulow, A., van Roekel, E., Boele, S., Denissen, J. J. A., & Keijsers, L. (2022). Parent-adolescent interaction quality and adolescent affect-An experience sampling study on effect heterogeneity. Child Development, 31, 31. https://dx.doi.org/10.1111/cdev.13733

Burnell, K., Andrade, F. C., & Hoyle, R. H. (2023). Longitudinal and daily associations between adolescent self-control and digital technology use. Developmental Psychology, 59(4), 720-732. https://dx.doi.org/10.1037/dev0001444

Burnell, K., George, M. J., Jensen, M., Hoyle, R. H., & Odgers, C. L. (2022). Associations Between Adolescents' Daily Digital Technology Use and Sleep. Journal of Adolescent Health, 70(3), 450-456. https://dx.doi.org/10.1016/j.jadohealth.2021.09.033

Butler, J. M., Whalen, C. K., & Jamner, L. D. (2009). Bummed out now, feeling sick later: weekday versus weekend negative affect and physical symptom reports in high school freshmen. Journal of Adolescent Health, 44(5), 452-457. https://dx.doi.org/10.1016/j.jadohealth.2008.09.005

Byrd, A. L., Vine, V., Beeney, J. E., Scott, L. N., Jennings, J. R., & Stepp, S. D. (2020). RSA reactivity to parent-child conflict as a predictor of dysregulated emotion and behavior in daily life. Psychological Medicine, 1-9. https://dx.doi.org/10.1017/S0033291720002810

* Byrd, A. L., Vine, V., Frigoletto, O. A., Vanwoerden, S., & Stepp, S. D. (2022). A Multi-Method Investigation of Parental Responses to Youth Emotion: Prospective Effects on Emotion Dysregulation and Reactive Aggression in Daily Life. Research on Child and Adolescent Psychopathology, 50(2), 117-131. https://dx.doi.org/10.1007/s10802-020-00754-0

* Byrnes, H. F., Miller, B. A., Morrison, C. N., Wiebe, D. J., Woychik, M., & Wiehe, S. E. (2017). Association of environmental indicators with teen alcohol use and problem behavior: Teens' observations vs. objectively-measured indicators. Health & Place, 43, 151-157. https://dx.doi.org/10.1016/j.healthplace.2016.12.004

* Caon, M., Prinelli, F., Angelini, L., Carrino, S., Mugellini, E., Orte, S., Serrano, J. C. E., Atkinson, S., Martin, A., & Adorni, F. (2022). PEGASO e-Diary: User Engagement and Dietary Behavior Change of a Mobile Food Record for Adolescents. Frontiers in Nutrition, 9, 727480. https://dx.doi.org/10.3389/fnut.2022.727480

* Casperson, S. L., Sieling, J., Moon, J., Johnson, L., Roemmich, J. N., & Whigham, L. (2015). A Mobile Phone Food Record App to Digitally Capture Dietary Intake for Adolescents in a Free-Living Environment: Usability Study. Jmir Mhealth and Uhealth, 3(1), 12, Article e30. 10.2196/mhealth.3324

* Chandler, K. D., Hodge, C. J., McElvaine, K., Olschewski, E. J., Melton, K. K., & Deboeck, P. (2022). Challenges of ecological momentary assessments to study family leisure: Participants' perspectives. Journal of Leisure Research, 53(1), 159-165. 10.1080/00222216.2021.2001398

* Chen, Y. R., Ng, D. Y., Tseng, M. H., Bundy, A., & Cordier, R. (2023). The impact of coping behaviors on perceived competence and social anxiety in the everyday social engagement of autistic adolescents. Autism, 13623613231196773. https://dx.doi.org/10.1177/13623613231196773

* Cheng, C. W., Brown, C. R., Venugopalan, J., & Wang, M. D. (2020). Towards an Effective Patient Health Engagement System Using Cloud-Based Text Messaging Technology. IEEE Journal of Translational Engineering in Health and Medicine, 8, 2700107. https://dx.doi.org/10.1109/JTEHM.2018.2868358

* Chin, T., Rickard, N. S., & Vella-Brodrick, D. A. (2016). Development and feasibility of a mobile experience sampling application for tracking program implementation in youth well-being programs. Psychology of Well-Being, 6, 1.

Christensen, C. G., Bickham, D., Ross, C. S., & Rich, M. (2015). Multitasking With Television Among Adolescents. Journal of Broadcasting & Electronic Media, 59(1), 130-148.

* Chun, S. K., Benjamin, K. S., & Mezulis, A. H. (2022). Investigating Affective Responding to Daily Positive Events Among Adolescents Using Ecological Momentary Assessment. Journal of Early Adolescence, 42(4), 542-564. 10.1177/02724316211058066

* Collins, R. L., Martino, S. C., Kovalchik, S. A., Becker, K. M., Shadel, W. G., & D'Amico, E. J. (2016). Alcohol Advertising Exposure Among Middle School-Age Youth: An Assessment Across All Media and Venues. Journal of Studies on Alcohol & Drugs, 77(3), 384-392.

Collins, R. L., Martino, S. C., Kovalchik, S. A., D'Amico, E. J., Shadel, W. G., Becker, K. M., & Tolpadi, A. (2017). Exposure to alcohol advertising and adolescents' drinking beliefs: Role of message interpretation. Health Psychology, 36(9), 890-897. https://dx.doi.org/10.1037/hea0000521

Colvin, P. J., & Mermelstein, R. J. (2010). Adolescents' smoking outcome expectancies and acute emotional responses following smoking. Nicotine & Tobacco Research, 12(12), 1203-1210. https://dx.doi.org/10.1093/ntr/ntq169

* Comulada, W. S., Lightfoot, M., Swendeman, D., Grella, C., & Wu, N. (2015). Compliance to Cell Phone-Based EMA Among Latino Youth in Outpatient Treatment. Journal of Ethnicity in Substance Abuse, 14(3), 232-250. https://dx.doi.org/10.1080/15332640.2014.986354

* Connelly, M., Anthony, K. K., Sarniak, R., Bromberg, M. H., Gil, K. M., & Schanberg, L. E. (2010). Parent pain responses as predictors of daily activities and mood in children with juvenile idiopathic arthritis: the utility of electronic diaries. Journal of Pain and Symptom Management, 39(3), 579-590. https://dx.doi.org/10.1016/j.jpainsymman.2009.07.013

Connelly, M., & Bickel, J. (2011). An electronic daily diary process study of stress and health behavior triggers of primary headaches in children. Journal of Pediatric Psychology, 36(8), 852-862. https://dx.doi.org/10.1093/jpepsy/jsr017

Connelly, M., Bromberg, M. H., Anthony, K. K., Gil, K. M., Franks, L., & Schanberg, L. E. (2012). Emotion regulation predicts pain and functioning in children with juvenile idiopathic arthritis: an electronic diary study. Journal of Pediatric Psychology, 37(1), 43-52. https://dx.doi.org/10.1093/jpepsy/jsr088

* Connelly, M., Miller, T., Gerry, G., & Bickel, J. (2010). Electronic momentary assessment of weather changes as a trigger of headaches in children. Headache, 50(5), 779-789. https://dx.doi.org/10.1111/j.1526-4610.2009.01586.x

* Connelly, M. A., & Boorigie, M. E. (2021). Feasibility of using 'SMARTER' methodology for monitoring precipitating conditions of pediatric migraine episodes. Headache, 61(3), 500-510. https://dx.doi.org/10.1111/head.14028

* Cordier, R., Brown, N., Chen, Y. W., Wilkes-Gillan, S., & Falkmer, T. (2016). Piloting the use of experience sampling method to investigate the everyday social experiences of children with Asperger syndrome/high functioning autism. Developmental

Neurorehabilitation, 19(2), 103-110. https://dx.doi.org/10.3109/17518423.2014.915244

* Corwin, D. J., Orchinik, J., D'Alonzo, B., Agarwal, A. K., Pettijohn, K. W., Master, C. L., & Wiebe, D. J. (2023). A Randomized Trial of Incentivization to Maximize Retention for Real-Time Symptom and Activity Monitoring Using Ecological Momentary Assessment in Pediatric Concussion. Pediatric Emergency Care, 39(7), 488-494. https://dx.doi.org/10.1097/PEC.0000000000002870

* Crooke, A. H., Reid, S. C., Kauer, S. D., McKenzie, D. P., Hearps, S. J., Khor, A. S., & Forbes, A. B. (2013). Temporal mood changes associated with different levels of adolescent drinking: using mobile phones and experience sampling methods to explore motivations for adolescent alcohol use. Drug & Alcohol Review, 32(3), 262-268. https://dx.doi.org/10.1111/dar.12034

Cursio, J. F., Mermelstein, R. J., & Hedeker, D. (2019). Latent trait shared-parameter mixed models for missing ecological momentary assessment data. Statistics in Medicine, 38(4), 660-673. https://dx.doi.org/10.1002/sim.7989

Cushing, C., Marker, A., Bejarano, C., Crick, C., & Huffhines, L. (2017). Latent Variable Mixture Modeling of Ecological Momentary Assessment Data: Implications for Screening and Adolescent Mood Profiles. Journal of Child & Family Studies, 26(6), 1565-1572. 10.1007/s10826-017-0689-5

Cushing, C. C., Bejarano, C. M., Mitchell, T. B., Noser, A. E., & Crick, C. J. (2018). Individual Differences in Negative Affectivity and Physical Activity in Adolescents: An Ecological Momentary Assessment Study. Journal of Child & Family Studies, 27(9), 2772-2779. 10.1007/s10826-018-1128-y

* Cushing, C. C., Kichline, T., Blossom, J. B., Friesen, C. A., & Schurman, J. V. (2019). Tailoring Individualized Evaluation of Pediatric Abdominal Pain Using Ecological Momentary Assessment (EMA): A Pilot Study Testing Feasibility and Acceptability. Clinical Journal of Pain, 35(11), 859-868. https://dx.doi.org/10.1097/AJP.0000000000000750

Cushing, C. C., Kichline, T., Friesen, C., & Schurman, J. V. (2021). Individual Differences in the Relationship Between Pain Fear, Avoidance, and Pain Severity in a Chronic Abdominal Pain Sample and the Moderating Effect of Child Age. Annals of Behavioral Medicine, 55(6), 571-579. https://dx.doi.org/10.1093/abm/kaaa096

* Cushing, C. C., Mitchell, T. B., Bejarano, C. M., Walters, R. W., Crick, C. J., & Noser, A. E. (2017). Bidirectional Associations Between Psychological States and Physical Activity in Adolescents: A mHealth Pilot Study. Journal of Pediatric Psychology, 42(5), 559-568. https://dx.doi.org/10.1093/jpepsy/jsw099

* Czyz, E. K., Koo, H. J., Al-Dajani, N., Kentopp, S. D., Jiang, A., & King, C. A. (2022). Temporal profiles of suicidal thoughts in daily life: Results from two mobile-based monitoring

studies with high-risk adolescents. Journal of Psychiatric Research, 153, 56-63. https://dx.doi.org/10.1016/j.jpsychires.2022.06.050

* D'Adamo, L., Sonnenblick, R. M., Juarascio, A. S., & Manasse, S. M. (2023). Relations between forms of dietary restraint, restriction, and loss-of-control eating among adolescents seeking weight control: An ecological momentary assessment study. Eating Behaviors, 50, 101791. https://dx.doi.org/10.1016/j.eatbeh.2023.101791

* Dallman, A. R., Bailliard, A., & Harrop, C. (2022). Identifying Predictors of Momentary Negative Affect and Depression Severity in Adolescents with Autism: An Exploratory Ecological Momentary Assessment Study. Journal of Autism and Developmental Disorders, 52(1), 291-303. https://dx.doi.org/10.1007/s10803-021-04935-8

D'Amico, E. J., Martino, S. C., Collins, R. L., Shadel, W. G., Tolpadi, A., Kovalchik, S., & Becker, K. M. (2017). Factors associated with younger adolescents' exposure to online alcohol advertising. Psychology of Addictive Behaviors, 31(2), 212-219. https://dx.doi.org/10.1037/adb0000224

* Davison, B. K., Quigg, R., & Skidmore, P. M. L. (2018). Pilot Testing a Photo-Based Food Diary in Nine- to Twelve- Year Old- Children from Dunedin, New Zealand. Nutrients, 10(2), 20. https://dx.doi.org/10.3390/nu10020240

* De Baere, S., Lefevre, J., De Martelaer, K., Philippaerts, R., & Seghers, J. (2015). Temporal patterns of physical activity and sedentary behavior in 10-14 year-old children on weekdays. BMC Public Health, 15, 791. https://dx.doi.org/10.1186/s12889-015-2093-7

De Baere, S., Philippaerts, R., De Martelaer, K., & Lefevre, J. (2016). Associations Between Objectively Assessed Components of Physical Activity and Health-Related Fitness in 10- to 14-Year-Old Children. Journal of Physical Activity & Health, 13(9), 993-1001. https://dx.doi.org/10.1123/jpah.2015-0596

De Baere, S., Seghers, J., Philippaerts, R., De Martelaer, K., & Lefevre, J. (2015). Intensity- and Domain-Specific Levels of Physical Activity and Sedentary Behavior in 10- to 14-Year-Old Children. Journal of Physical Activity & Health, 12(12), 1543-1550. https://dx.doi.org/10.1123/jpah.2014-0416

De France, K., Hicks, O., & Hollenstein, T. (2022). Understanding the association between reappraisal use and depressive symptoms during adolescence: the moderating influence of regulatory success. Cognition & Emotion, 1-9. https://dx.doi.org/10.1080/02699931.2022.2043245

* De France, K., & Hollenstein, T. (2021). Emotion Regulation Strategy Use and Success During Adolescence: Assessing the Role of Context. Journal of Research on Adolescence, 29, 29. https://dx.doi.org/10.1111/jora.12672

De France, K., & Hollenstein, T. (2021). Implicit theories of emotion and mental health during adolescence: the mediating role of emotion regulation. Cognition & Emotion, 35(2), 367-374. https://dx.doi.org/10.1080/02699931.2020.1817727

De Ridder, J., Pihet, S., Suter, M., & Caldara, R. (2016). Empathy in institutionalized adolescents with callous-unemotional traits: An ecological momentary assessment study of emotion recognition. Criminal Justice and Behavior, 43(5), 653-669. https://dx.doi.org/10.1177/0093854815618431

Demirtas, H., Hedeker, D., & Mermelstein, R. J. (2012). Simulation of massive public health data by power polynomials. Statistics in Medicine, 31(27), 3337-3346. 10.1002/sim.5362

* Dennis, M. L., Scott, C. K., Funk, R. R., & Nicholson, L. (2015). A Pilot Study to Examine the Feasibility and Potential Effectiveness of Using Smartphones to Provide Recovery Support for Adolescents. Substance Abuse, 36(4), 486-492. https://dx.doi.org/10.1080/08897077.2014.970323

* Di Giunta, L., Lunetti, C., Gliozzo, G., Rothenberg, W. A., Lansford, J. E., Eisenberg, N., Pastorelli, C., Basili, E., Fiasconaro, I., Thartori, E., Favini, A., & Virzi, A. T. (2022). Negative Parenting, Adolescents' Emotion Regulation, Self-Efficacy in Emotion Regulation, and Psychological Adjustment. International Journal of Environmental Research & Public Health [Electronic Resource], 19(4), 16. https://dx.doi.org/10.3390/ijerph19042251

* Dietvorst, E., Aukes, M. A., Legerstee, J. S., Vreeker, A., Hrehovcsik, M. M., Keijsers, L., & Hillegers, M. H. J. (2022). A Smartphone Serious Game for Adolescents (Grow It! App): Development, Feasibility, and Acceptance Study. JMIR Formative Research, 6(3), e29832. https://dx.doi.org/10.2196/29832

* Dietvorst, E., Hiemstra, M., Maciejewski, D., van Roekel, E., Bogt, T. T., Hillegers, M., & Keijsers, L. (2021). Grumpy or depressed? Disentangling typically developing adolescent mood from prodromal depression using experience sampling methods. Journal of Adolescence, 88, 25-35. https://dx.doi.org/10.1016/j.adolescence.2021.01.009

Dirk, J., & Schmiedek, F. (2017). Variability in children's working memory is coupled with perceived disturbance: An ambulatory assessment study in the school and out-of-school context. Research in Human Development, 14(3), 200-218. https://dx.doi.org/10.1080/15427609.2017.1340051

Do, B., Mason, T. B., Yi, L., Yang, C. H., & Dunton, G. F. (2021). Momentary associations between stress and physical activity among children using ecological momentary assessment. Psychology of Sport and Exercise, 55. https://dx.doi.org/10.1016/j.psychsport.2021.101935

Do, B., Yang, C. H., Lopez, N. V., Mason, T. B., Margolin, G., & Dunton, G. F. (2020). Investigating the momentary association between maternal support and children's fruit and vegetable consumption using ecological momentary assessment. Appetite, 150, 104667. https://dx.doi.org/10.1016/j.appet.2020.104667

Do, Q. B., McKone, K. M. P., Hamilton, J. L., Stone, L. B., Ladouceur, C. D., & Silk, J. S. (2023). The link between adolescent girls' interpersonal emotion regulation with parents and peers and depressive symptoms: A real-time investigation. Development and Psychopathology, 1-15. https://dx.doi.org/10.1017/S0954579423001359

Doan, S. N., Xie, B., Zhou, Y., Lei, X., & Reynolds, K. D. (2022). Loneliness and cravings for sugar-sweetened beverages among adolescents. Pediatric Obesity, 17(1), e12834. https://dx.doi.org/10.1111/ijpo.12834

* Domoff, S. E., Banga, C. A., Borgen, A. L., Foley, R. P., Robinson, C., Avery, K., & Gentile, D. A. (2021). Use of passive sensing to quantify adolescent mobile device usage: Feasibility, acceptability, and preliminary validation of the emoodie application. Human Behavior and Emerging Technologies, No Pagination Specified. https://dx.doi.org/10.1002/hbe2.247

Dougherty, E. N., Goldschmidt, A. B., Johnson, N. K., Badillo, K., Engel, S. G., & Haedt-Matt, A. A. (2022). Gender differences in the relation between interpersonal stress and momentary shape and weight concerns in youth with overweight/obesity. Body Image, 40, 249-255. https://dx.doi.org/10.1016/j.bodyim.2022.01.006

Dougherty, E. N., Randall, I., Haedt-Matt, A. A., Pila, E., Smith, K., Wang, S., Yang, C. H., Engel, S. G., & Goldschmidt, A. B. (2023). Accelerometer-Based Physical Activity and Shape and Weight Concerns Among Youth With Overweight and Obesity: A Pilot Exploratory Ecological Momentary Assessment Study. Childhood Obesity, 26, 26. https://dx.doi.org/10.1089/chi.2022.0236

* Doyle, C., Werner, E., Feng, T., Lee, S., Altemus, M., Isler, J. R., & Monk, C. (2015). Pregnancy distress gets under fetal skin: Maternal ambulatory assessment & sex differences in prenatal development. Developmental Psychobiology, 57(5), 607-625. https://dx.doi.org/10.1002/dev.21317

* Dunton, G., Dzubur, E., Li, M., Huh, J., Intille, S., & McConnell, R. (2016). Momentary Assessment of Psychosocial Stressors, Context, and Asthma Symptoms in Hispanic Adolescents. Behavior Modification, 40(1-2), 257-280. https://dx.doi.org/10.1177/0145445515608145

Dunton, G. F., Dzubur, E., Huh, J., Belcher, B. R., Maher, J. P., O'Connor, S., & Margolin, G. (2017). Daily Associations of Stress and Eating in Mother-Child Dyads. Health Education and Behavior, 44(3), 365-369. https://dx.doi.org/10.1177/1090198116663132

* Dunton, G. F., Dzubur, E., & Intille, S. (2016). Feasibility and Performance Test of a Real-Time Sensor-Informed Context-Sensitive Ecological Momentary Assessment to Capture Physical Activity. Journal of Medical Internet Research, 18(6), e106. https://dx.doi.org/10.2196/jmir.5398

Dunton, G. F., Huh, J., Leventhal, A. M., Riggs, N., Hedeker, D., Spruijt-Metz, D., & Pentz, M. A. (2014). Momentary assessment of affect, physical feeling states, and physical activity

in children. Health Psychology, 33(3), 255-263. https://dx.doi.org/10.1037/a0032640

Dunton, G. F., Intille, S. S., Wolch, J., & Pentz, M. A. (2012). Children's perceptions of physical activity environments captured through ecological momentary assessment: a validation study. Preventive Medicine, 55(2), 119-121. https://dx.doi.org/10.1016/j.ypmed.2012.05.015

Dunton, G. F., Intille, S. S., Wolch, J., & Pentz, M. A. (2012). Investigating the impact of a smart growth community on the contexts of children's physical activity using Ecological Momentary Assessment. Health & Place, 18(1), 76-84. https://dx.doi.org/10.1016/j.healthplace.2011.07.007

* Dunton, G. F., Kawabata, K., Intille, S., Wolch, J., & Pentz, M. A. (2012). Assessing the social and physical contexts of children's leisure-time physical activity: an ecological momentary assessment study. American Journal of Health Promotion, 26(3), 135-142. https://dx.doi.org/10.4278/ajhp.100211-QUAN-43

* Dunton, G. F., Liao, Y., Dzubur, E., Leventhal, A. M., Huh, J., Gruenewald, T., Margolin, G., Koprowski, C., Tate, E., & Intille, S. (2015). Investigating within-day and longitudinal effects of maternal stress on children's physical activity, dietary intake, and body composition: Protocol for the MATCH study. Contemporary Clinical Trials, 43, 142-154. https://dx.doi.org/10.1016/j.cct.2015.05.007

Dunton, G. F., Liao, Y., Intille, S., Wolch, J., & Pentz, M. A. (2011). Physical and social contextual influences on children's leisure-time physical activity: an ecological momentary assessment study. Journal of Physical Activity & Health, 8 Suppl 1, S103-108.

Dunton, G. F., Liao, Y., Intille, S. S., Spruijt-Metz, D., & Pentz, M. (2011). Investigating children's physical activity and sedentary behavior using ecological momentary assessment with mobile phones. Obesity, 19(6), 1205-1212. https://dx.doi.org/10.1038/oby.2010.302

Dunton, G. F., Wang, W. L., Intille, S. S., Dzubur, E., Ponnada, A., & Hedeker, D. (2022). How acute affect dynamics impact longitudinal changes in physical activity among children. Journal of Behavioral Medicine, 28, 28. https://dx.doi.org/10.1007/s10865-022-00282-w

* Dunton, G. F., Whalen, C. K., Jamner, L. D., & Floro, J. N. (2007). Mapping the social and physical contexts of physical activity across adolescence using ecological momentary assessment. Annals of Behavioral Medicine, 34(2), 144-153.

Dunton, G. F., Whalen, C. K., Jamner, L. D., Henker, B., & Floro, J. N. (2005). Using ecologic momentary assessment to measure physical activity during adolescence. American Journal of Preventive Medicine, 29(4), 281-287.

* Duvenage, M., Correia, H., Uink, B., Barber, B. L., Donovan, C. L., & Modecki, K. L. (2020). Technology can sting when reality bites: Adolescents' frequent online coping is

ineffective with momentary stress. Computers in Human Behavior, 102, 248-259. 10.1016/j.chb.2019.08.024

Dvorak, R. D., Waters, A. J., MacIntyre, J. M., & Gwaltney, C. J. (2018). Affect, craving, and cognition: An EMA study of ad libitum adolescent smoking. Psychology of Addictive Behaviors, 32(6), 583-594. https://dx.doi.org/10.1037/adb0000392

* Dzubur, E., Huh, J., Maher, J. P., Intille, S. S., & Dunton, G. F. (2018). Response patterns and intra-dyadic factors related to compliance with ecological momentary assessment among mothers and children. Translational Behavioral Medicine, 8(2), 233-242. https://dx.doi.org/10.1093/tbm/ibx002

Ebrahimian, S., Zink, J., Yang, C. H., Yu, Q., Imm, K., Nicolo, M., Dunton, G. F., & Belcher, B. R. (2022). Momentary intentions and perceived behavioral control are within-person predictors of sedentary leisure time: preliminary findings from an ecological momentary assessment study in adolescents. Journal of Behavioral Medicine, 01, 01. https://dx.doi.org/10.1007/s10865-022-00309-2

Egbert, A. H., Haedt-Matt, A., Smith, K. E., Culbert, K., Engel, S., & Goldschmidt, A. B. (2020). Momentary associations between positive affect dimensions and dysregulated eating during puberty in a diverse sample of youth with overweight/obesity. International Journal of Eating Disorders, 53(10), 1667-1677. https://dx.doi.org/10.1002/eat.23342

* El Dahr, Y., Perquier, F., Moloney, M., Woo, G., Dobrin-De Grace, R., Carvalho, D., Addario, N., Cameron, E. E., Roos, L. E., Szatmari, P., & Aitken, M. (2023). Feasibility of Using Research Electronic Data Capture (REDCap) to Collect Daily Experiences of Parent-Child Dyads: Ecological Momentary Assessment Study. JMIR Formative Research, 7, e42916. https://dx.doi.org/10.2196/42916

* Elovainio, M., Kuula, L., Halonen, R., & Pesonen, A. K. (2020). Dynamic fluctuations of emotional states in adolescents with delayed sleep phase-A longitudinal network modeling approach. Journal of Affective Disorders, 276, 467-475. https://dx.doi.org/10.1016/j.jad.2020.07.050

Ernst, J., Ruckert, F., Ollmann, T. M., Voss, C., Kische, H., Knappe, S., & Beesdo-Baum, K. (2023). Social Interactions in Everyday Life of Socially Anxious Adolescents: Effects on Mental State, Anxiety, and Depression. Research on Child and Adolescent Psychopathology, 28, 28. https://dx.doi.org/10.1007/s10802-023-01121-5

Esposito, E. C., Duan, A. M., Kearns, J. C., Kleiman, E. M., Conwell, Y., & Glenn, C. R. (2022). Measuring Adolescents' Self-injurious Thoughts and Behaviors: Comparing Ecological Momentary Assessment to a Traditional Interview. Research on Child and Adolescent Psychopathology, 07, 07. https://dx.doi.org/10.1007/s10802-022-00907-3

Evans, S. C., Hamilton, J. L., Boyd, S. I., Karlovich, A. R., Ladouceur, C. D., Silk, J. S., & Bylsma, L. M. (2023). Daily Associations Between Sleep and Affect in Youth at Risk for

Psychopathology: The Moderating Role of Externalizing Symptoms. Research on Child and Adolescent Psychopathology, 05, 05. https://dx.doi.org/10.1007/s10802-023-01087-4

* Ewing, E. L., Xia, M., & Gunn, H. E. (2023). Affiliative Parent-Adolescent Bedtime and Waketime Interactions are Associated with Adolescent Sleep. Behavioral Sleep Medicine, 1-11. https://dx.doi.org/10.1080/15402002.2023.2217970

* Ferguson, K., Gunthert, K., Kaidbey, J. H., Parr, M., Visek, A. J., Sacheck, J. M., & Sylvetsky, A. C. (2023). Behavioral Patterns of Sugary Drink Consumption among African American Adolescents: A Pilot and Feasibility Study Using Ecological Momentary Assessment. Nutrients, 15(9), 02. https://dx.doi.org/10.3390/nu15092171

* Fiedler, J., Seiferth, C., Eckert, T., Woll, A., & Wunsch, K. (2022). Sleep quality, valence, energetic arousal, and calmness as predictors of device-based measured physical activity during a three-week mHealth intervention An ecological momentary assessment study within the SMARTFAMILY trial. German Journal of Exercise and Sport Research, 11. 10.1007/s12662-022-00809-y

Finan, L. J., & Lipperman-Kreda, S. (2020). Changes in Drinking Contexts over the Night Course: Concurrent and Lagged Associations with Adolescents' Nightly Alcohol Use. Alcoholism: Clinical & Experimental Research, 44(12), 2611-2617. https://dx.doi.org/10.1111/acer.14486

* Floro, J. N., Dunton, G. E., & Delfino, R. J. (2009). Assessing physical activity in children with asthma: convergent validity between accelerometer and electronic diary data. Research Quarterly for Exercise and Sport, 80(2), 153-163.

* Fortgang, R. G., Wang, S. B., Millner, A. J., Reid-Russell, A., Beukenhorst, A. L., Kleiman, E. M., Bentley, K. H., Zuromski, K. L., Al-Suwaidi, M., Bird, S. A., Buonopane, R., DeMarco, D., Haim, A., Joyce, V. W., Kastman, E. K., Kilbury, E., Lee, H.-I. S., Mair, P., Nash, C. C., Onnela, J.-P., Smoller, J. W., & Nock, M. K. (2021). Increase in suicidal thinking during COVID-19. Clinical Psychological Science, 9(3), 482-488. https://dx.doi.org/10.1177/2167702621993857

* Fortier, M. A., Chung, W. W., Martinez, A., Gago-Masague, S., & Sender, L. (2016). Pain buddy: A novel use of m-health in the management of children's cancer pain. Computers in Biology and Medicine, 76, 202-214. 10.1016/j.compbiomed.2016.07.012

* Fuller-Tyszkiewicz, M., McCabe, M., Skouteris, H., Richardson, B., Nihill, K., Watson, B., & Solomon, D. (2015). Does body satisfaction influence self-esteem in adolescents' daily lives? An experience sampling study. Journal of Adolescence, 45, 11-19. https://dx.doi.org/10.1016/j.adolescence.2015.08.009

Fürtjes, S., Seidel, M., Diestel, S., Wolff, M., King, J. A., Hellerhoff, I., Bernadoni, F., Gramatke, K., Goschke, T., Roessner, V., & Ehrlich, S. (2022). Real-life self-control conflicts in

anorexia nervosa: An ecological momentary assessment investigation. European Psychiatry, 65(1). 10.1192/j.eurpsy.2022.29

Furtjes, S., Seidel, M., King, J. A., Biemann, R., Roessner, V., & Ehrlich, S. (2018). Rumination in anorexia nervosa: Cognitive-affective and neuroendocrinological aspects. Behaviour Research and Therapy, 111, 92-98. https://dx.doi.org/10.1016/j.brat.2018.10.001

Gajos, J. M., Russell, M. A., Odgers, C. L., Hoyle, R. H., & Copeland, W. E. (2022). Pubertal timing moderates the same-day coupling between family hassles and negative affect in girls and boys. Development and Psychopathology, 1-14. https://dx.doi.org/10.1017/S0954579422000591

Galeano-Keiner, E. M., Neubauer, A. B., Irmer, A., & Schmiedek, F. (2022). Daily fluctuations in children's working memory accuracy and precision: Variability at multiple time scales and links to daily sleep behavior and fluid intelligence. Cognitive Development, 64, 20, Article 101260. 10.1016/j.cogdev.2022.101260

* Galvan, A., & McGlennen, K. M. (2012). Daily stress increases risky decision-making in adolescents: A preliminary study. Developmental Psychobiology, 54(4), 433-440. https://dx.doi.org/10.1002/dev.20602

* Garcia, C., Hardeman, R. R., Kwon, G., Lando-King, E., Zhang, L., Genis, T., Brady, S. S., & Kinder, E. (2014). Teenagers and texting: use of a youth ecological momentary assessment system in trajectory health research with latina adolescents. JMIR MHealth and UHealth, 2(1), e3. https://dx.doi.org/10.2196/mhealth.2576

Garcia, C., Zhang, L., Holt, K., Hardeman, R., & Peterson, B. (2014). Latina adolescent sleep and mood: an ecological momentary assessment pilot study. Journal of Child and Adolescent Psychiatric Nursing, 27(3), 132-141. https://dx.doi.org/10.1111/jcap.12082

Garrett, S. L., Burnell, K., Armstrong-Carter, E. L., Prinstein, M. J., & Telzer, E. H. (2023). Linking video chatting, phone calling, text messaging, and social media with peers to adolescent connectedness. Journal of Research on Adolescence, 29, 29. https://dx.doi.org/10.1111/jora.12871

Gaultney, A. C., Bromberg, M. H., Connelly, M., Spears, T., & Schanberg, L. E. (2017). Parent and Child Report of Pain and Fatigue in JIA: Does Disagreement between Parent and Child Predict Functional Outcomes? Children-Basel, 4(2), 10, Article 11. 10.3390/children4020011

Geiser, C., Bishop, J., Lockhart, G., Shiffman, S., & Grenard, J. L. (2013). Analyzing latent state-trait and multiple-indicator latent growth curve models as multilevel structural equation models. Frontiers in Psychology, 4, 975. https://dx.doi.org/10.3389/fpsyg.2013.00975

George, M. J., Rivenbark, J. G., Russell, M. A., Ng'eno, L., Hoyle, R. H., & Odgers, C. L. (2019). Evaluating the Use of Commercially Available Wearable Wristbands to Capture

Adolescents' Daily Sleep Duration. Journal of Research on Adolescence, 29(3), 613-626. https://dx.doi.org/10.1111/jora.12467

George, M. J., Russell, M. A., Piontak, J. R., & Odgers, C. L. (2018). Concurrent and Subsequent Associations Between Daily Digital Technology Use and High-Risk Adolescents' Mental Health Symptoms. Child Development, 89(1), 78-88. https://dx.doi.org/10.1111/cdev.12819

* Gibson, F., Aldiss, S., Taylor, R. M., Maguire, R., McCann, L., Sage, M., & Kearney, N. (2010). Utilization of the Medical Research Council evaluation framework in the development of technology for symptom management: the ASyMS-YG Study. Cancer Nursing, 33(5), 343-352. https://dx.doi.org/10.1097/NCC.0b013e3181cb4bad

Glenn, C. R., Kleiman, E. M., Kandlur, R., Esposito, E. C., & Liu, R. T. (2021). Thwarted Belongingness Mediates Interpersonal Stress and Suicidal Thoughts: An Intensive Longitudinal Study with High-risk Adolescents. Journal of Clinical Child and Adolescent Psychology, 1-17. https://dx.doi.org/10.1080/15374416.2021.1969654

Glenn, C. R., Kleiman, E. M., Kearns, J. C., Boatman, A. E., Conwell, Y., Alpert-Gillis, L. J., & Pigeon, W. (2021). Sleep problems predict next-day suicidal thinking among adolescents: A multimodal real-time monitoring study following discharge from acute psychiatric care. Development and Psychopathology, 33(5), 1701-1721, Article Pii s0954579421000699. 10.1017/s0954579421000699

* Glenn, C. R., Kleiman, E. M., Kearns, J. C., Santee, A. C., Esposito, E. C., Conwell, Y., & Alpert-Gillis, L. J. (2022). Feasibility and Acceptability of Ecological Momentary Assessment with High-Risk Suicidal Adolescents Following Acute Psychiatric Care. Journal of Clinical Child and Adolescent Psychology, 51(1), 32-48. https://dx.doi.org/10.1080/15374416.2020.1741377

* Glista, D., O'Hagan, R., Van Eeckhoutte, M., Lai, Y., & Scollie, S. (2021). The use of ecological momentary assessment to evaluate real-world aided outcomes with children. International Journal of Audiology, 60(sup1), S68-S78. https://dx.doi.org/10.1080/14992027.2021.1881629

Goetz, T., Bieg, M., Ludtke, O., Pekrun, R., & Hall, N. C. (2013). Do girls really experience more anxiety in mathematics? Psychological Science, 24(10), 2079-2087. https://dx.doi.org/10.1177/0956797613486989

Goetz, T., Frenzel, A. C., Hall, N. C., Nett, U. E., Pekrun, R., & Lipnevich, A. A. (2014). Types of boredom: An experience sampling approach. Motivation and Emotion, 38(3), 401-419. https://dx.doi.org/10.1007/s11031-013-9385-y

* Goetz, T., Keller, M. M., Ludtke, O., Nett, U. E., & Lipnevich, A. A. (2020). The dynamics of real-time classroom emotions: Appraisals mediate the relation between students' perceptions of teaching and their emotions. Journal of Educational Psychology, 112(6), 1243-1260. https://dx.doi.org/10.1037/edu0000415

Goetz, T., Ludtke, O., Nett, U. E., Keller, M. M., & Lipnevich, A. A. (2013). Characteristics of teaching and students' emotions in the classroom: Investigating differences across domains. Contemporary Educational Psychology, 38(4), 383-394. https://dx.doi.org/10.1016/j.cedpsych.2013.08.001

Goetz, T., Sticca, F., Pekrun, R., Murayama, K., & Elliot, A. J. (2016). Intraindividual relations between achievement goals and discrete achievement emotions: An experience sampling approach. Learning and Instruction, 41, 115-125. https://dx.doi.org/10.1016/j.learninstruc.2015.10.007

* Gold, B. D., Pilmer, B., Kierkus, J., Hunt, B., Perez, M. C., & Gremse, D. (2017). Dexlansoprazole for Heartburn Relief in Adolescents with Symptomatic, Nonerosive Gastro-esophageal Reflux Disease. Digestive Diseases and Sciences, 62(11), 3059-3068. https://dx.doi.org/10.1007/s10620-017-4743-3

Goldschmidt, A. B., Evans, E. W., Saletin, J. M., O'Sullivan, K., Koren, D., Engel, S. G., & Haedt-Matt, A. (2020). Naturalistic, multimethod exploratory study of sleep duration and quality as predictors of dysregulated eating in youth with overweight and obesity. Appetite, 146, 104521. https://dx.doi.org/10.1016/j.appet.2019.104521

* Goldschmidt, A. B., Goldstein, S. P., Schmiedek, F., Stalvey, E., Irizarry, B., & Thomas, J. G. (2023). State-level working memory and dysregulated eating in children and adolescents: An exploratory ecological momentary assessment study. International Journal of Eating Disorders, 27, 27. https://dx.doi.org/10.1002/eat.24087

Goldschmidt, A. B., Mason, T. B., Smith, K. E., Egbert, A. H., Engel, S. G., & Haedt-Matt, A. (2022). Typology of eating episodes in children and adolescents with overweight/obesity. Eating Behaviors, 44, 101596. https://dx.doi.org/10.1016/j.eatbeh.2022.101596

* Goldschmidt, A. B., Smith, K. E., Crosby, R. D., Boyd, H. K., Dougherty, E., Engel, S. G., & Haedt-Matt, A. (2018). Ecological momentary assessment of maladaptive eating in children and adolescents with overweight or obesity. International Journal of Eating Disorders, 51(6), 549-557. https://dx.doi.org/10.1002/eat.22864

Goldschmidt, A. B., Smith, K. E., Lavender, J. M., Engel, S. G., & Haedt-Matt, A. (2019). Trait-level facets of impulsivity and momentary, naturalistic eating behavior in children and adolescents with overweight/obesity. Journal of Psychiatric Research, 110, 24-30. https://dx.doi.org/10.1016/j.jpsychires.2018.12.018

* Grenard, J. L., Stacy, A. W., Shiffman, S., Baraldi, A. N., MacKinnon, D. P., Lockhart, G., Kisbu-Sakarya, Y., Boyle, S., Beleva, Y., Koprowski, C., Ames, S. L., & Reynolds, K. D. (2013). Sweetened drink and snacking cues in adolescents: a study using ecological momentary assessment. Appetite, 67, 61-73. https://dx.doi.org/10.1016/j.appet.2013.03.016

Griffith, J. M., Farrell-Rosen, T. S., & Hankin, B. L. (2023). Daily life positive affect regulation in early adolescence: Associations with symptoms of depression. Emotion, 23(3), 664-677. https://dx.doi.org/10.1037/emo0001129

* Griffith, J. M., & Hankin, B. L. (2021). Affective Benefits of Parental Engagement with Adolescent Positive Daily Life Experiences. Journal of Youth & Adolescence, 50(10), 2036-2051. https://dx.doi.org/10.1007/s10964-021-01484-2

* Gwaltney, C., Ivanescu, C., Karlsson, L., Warholic, N., Kjems, L., & Horn, P. (2022). Validation of the PRUCISION Instruments in Pediatric Patients with Progressive Familial Intrahepatic Cholestasis. Advances in Therapy, 39(11), 5105-5125. https://dx.doi.org/10.1007/s12325-022-02262-7

Gwaltney, C. J., Bartolomei, R., Colby, S. M., & Kahler, C. W. (2008). Ecological momentary assessment of adolescent smoking cessation: a feasibility study. Nicotine & Tobacco Research, 10(7), 1185-1190. https://dx.doi.org/10.1080/14622200802163118

Ha, T., van Roekel, E., Iida, M., Kornienko, O., Engels, R., & Kuntsche, E. (2019). Depressive Symptoms Amplify Emotional Reactivity to Daily Perceptions of Peer Rejection in Adolescence. Journal of Youth & Adolescence, 48(11), 2152-2164. https://dx.doi.org/10.1007/s10964-019-01146-4

Hagemann, N., Kirtley, O. J., Lafit, G., Vancampfort, D., Wampers, M., Decoster, J., Derom, C., Guloksuz, S., De Hert, M., Jacobs, N., Menne-Lothmann, C., Rutten, B. P. F., Thiery, E., van Os, J., van Winkel, R., Wichers, M., & Myin-Germeys, I. (2023). Coping and sleep quality in youth: An Experience Sampling study. Journal of Adolescence, 95(3), 566-583. https://dx.doi.org/10.1002/jad.12137

* Hamilton, J. L., Chand, S., Reinhardt, L., Ladouceur, C. D., Silk, J. S., Moreno, M., Franzen, P. L., & Bylsma, L. M. (2020). Social media use predicts later sleep timing and greater sleep variability: An ecological momentary assessment study of youth at high and low familial risk for depression. Journal of Adolescence, 83, 122-130. https://dx.doi.org/10.1016/j.adolescence.2020.07.009

* Hamilton, J. L., Do, Q. B., Choukas-Bradley, S., Ladouceur, C. D., & Silk, J. S. (2021). Where it Hurts the Most: Peer Interactions on Social Media and in Person are Differentially Associated with Emotional Reactivity and Sustained Affect Among Adolescent Girls. Research on Child and Adolescent Psychopathology, 49(2), 155-167. https://dx.doi.org/10.1007/s10802-020-00725-5

* Hank, P., & Baltes-Gotz, B. (2019). The stability of self-esteem variability: A real-time assessment. Journal of Research in Personality, 79, 143-150. https://dx.doi.org/10.1016/j.jrp.2019.03.004

* Hao, H., Eckel, S. P., Hosseini, A., Van Vliet, E. D. S., Dzubur, E., Dunton, G., Chang, S. Y., Craig, K., Rocchio, R., Bastain, T., Gilliland, F., Okelo, S., Ross, M. K., Sarrafzadeh, M., Bui, A. A. T., & Habre, R. (2022). Daily Associations of Air Pollution and Pediatric Asthma Risk Using the Biomedical REAI-Time Health Evaluation (BREATHE) Kit. International

Journal of Environmental Research and Public Health, 19(6). 10.3390/ijerph19063578

Hasmi, L., Drukker, M., Guloksuz, S., Menne-Lothmann, C., Decoster, J., van Winkel, R., Collip, D., Delespaul, P., De Hert, M., Derom, C., Thiery, E., Jacobs, N., Rutten, B. P. F., Wichers, M., & van Os, J. (2017). Network Approach to Understanding Emotion Dynamics in Relation to Childhood Trauma and Genetic Liability to Psychopathology: Replication of a Prospective Experience Sampling Analysis. Frontiers in Psychology, 8, 1908. https://dx.doi.org/10.3389/fpsyg.2017.01908

Hausen, J. E., Moller, J., Greiff, S., & Niepel, C. (2022). Students' personality and state academic self-concept: Predicting differences in mean level and within-person variability in everyday school life. Journal of Educational Psychology, 114(6), 1394-1411. https://dx.doi.org/10.1037/edu0000760

* Heathcote, L. C., Cunningham, S. J., Webster, S. N., Tanna, V., Mattke, E., Loecher, N., Spunt, S. L., Simon, P., Dahl, G., Walentynowicz, M., Murnane, E., Tutelman, P. R., Schapira, L., Simons, L. E., & Mueller, C. (2022). Smartphone-based Ecological Momentary Assessment to study 'scanxiety' among Adolescent and Young Adult survivors of childhood cancer: A feasibility study. Psycho Oncology, 11, 11. https://dx.doi.org/10.1002/pon.5935

Hedeker, D., Demirtas, H., & Mermelstein, R. J. (2009). A mixed ordinal location scale model for analysis of Ecological Momentary Assessment (EMA) data. Statistics & its Interface, 2(4), 391-401.

Hedeker, D., Mermelstein, R. J., Berbaum, M. L., & Campbell, R. T. (2009). Modeling mood variation associated with smoking: an application of a heterogeneous mixed-effects model for analysis of ecological momentary assessment (EMA) data. Addiction, 104(2), 297-307. https://dx.doi.org/10.1111/j.1360-0443.2008.02435.x

Hedeker, D., Mermelstein, R. J., & Demirtas, H. (2008). An application of a mixed-effects location scale model for analysis of Ecological Momentary Assessment (EMA) data. Biometrics, 64(2), 627-634.

Hedeker, D., Mermelstein, R. J., & Demirtas, H. (2012). Modeling between-subject and within-subject variances in ecological momentary assessment data using mixed-effects location scale models. Statistics in Medicine, 31(27), 3328-3336. https://dx.doi.org/10.1002/sim.5338

* Helgeson, V. S., Lopez, L. C., & Kamarck, T. (2009). Peer relationships and diabetes: retrospective and ecological momentary assessment approaches. Health Psychology, 28(3), 273-282. https://dx.doi.org/10.1037/a0013784

Henker, B., Whalen, C. K., Jamner, L. D., & Delfino, R. J. (2002). Anxiety, affect, and activity in teenagers: monitoring daily life with electronic diaries. Journal of the American Academy of Child and Adolescent Psychiatry, 41(6), 660-670.

* Hennig, T., Krkovic, K., & Lincoln, T. M. (2017). What predicts inattention in adolescents? An experience-sampling study comparing chronotype, subjective, and objective sleep parameters. Sleep Medicine, 38, 58-63. https://dx.doi.org/10.1016/j.sleep.2017.07.009

Hennig, T., & Lincoln, T. M. (2018). Sleeping Paranoia Away? An Actigraphy and Experience-Sampling Study with Adolescents. Child Psychiatry and Human Development, 49(1), 63-72. https://dx.doi.org/10.1007/s10578-017-0729-9

Henry, S. L., Jamner, L. D., & Whalen, C. K. (2012). I (should) Need a Cigarette: Adolescent Social Anxiety and Cigarette Smoking. Annals of Behavioral Medicine, 43(3), 383-393. 10.1007/s12160-011-9340-7

Hermans, K., Kirtley, O. J., Kasanova, Z., Achterhof, R., Hagemann, N., Hiekkaranta, A. P., Lecei, A., Zapata-Fonseca, L., Lafit, G., Fossion, R., Froese, T., & Myin-Germeys, I. (2022). Ecological and Convergent Validity of Experimentally and Dynamically Assessed Capacity for Social Contingency Detection Using the Perceptual Crossing Experiment in Adolescence. Assessment, 10731911221083613. https://dx.doi.org/10.1177/10731911221083613

Heshmati, S., DavyRomano, E., Chow, C., Doan, S. N., & Reynolds, K. D. (2023). Negative emodiversity is associated with emotional eating in adolescents: An examination of emotion dynamics in daily life. Journal of Adolescence, 95(1), 115-130. https://dx.doi.org/10.1002/jad.12103

Hiekkaranta, A. P., Kirtley, O. J., Lafit, G., Decoster, J., Derom, C., de Hert, M., Guloksuz, S., Jacobs, N., Menne-Lothmann, C., Rutten, B. P. F., Thiery, E., van Os, J., van Winkel, R., Wichers, M., & Myin-Germeys, I. (2021). Emotion regulation in response to daily negative and positive events in youth: The role of event intensity and psychopathology. Behaviour Research and Therapy, 144, 103916. https://dx.doi.org/10.1016/j.brat.2021.103916

* Hoeppner, B. B., Kahler, C. W., & Gwaltney, C. J. (2014). Relationship between momentary affect states and self-efficacy in adolescent smokers. Health Psychology, 33(12), 1507-1517. https://dx.doi.org/10.1037/hea0000075

* Holton, N. S. (2017). Eudaimonia and engagement in the classroom: Using experience sampling in an exploratory study of well-being in high school students. Dissertation Abstracts International Section A: Humanities and Social Sciences, 77(9-A(E)). https://doi.org/doi:10.25335/b1ea-sg35

* Hoy, W. S., Antoun, J. S., Lin, W., Chandler, N., Merriman, T., & Farella, M. (2018). Ecological momentary assessment of pain in adolescents undergoing orthodontic treatment using a smartphone app. Seminars in Orthodontics, 24(2), 209-216. 10.1053/j.sodo.2018.04.002

* Hunter, J. F., Acevedo, A. M., Gago-Masague, S., Kain, A., Yun, C., Torno, L., Jenkins, B. N., & Fortier, M. A. (2020). A pilot study of the preliminary efficacy of Pain Buddy: A novel

intervention for the management of children's cancer-related pain. Pediatric Blood & Cancer, 67(10), e28278. https://dx.doi.org/10.1002/pbc.28278

Inkinen, J., Klager, C., Juuti, K., Schneider, B., Salmela-Aro, K., Krajcik, J., & Lavonen, J. (2020). High school students' situational engagement associated with scientific practices in designed science learning situations. Science Education, 104(4), 667-692. https://dx.doi.org/10.1002/sce.21570

* Jacob, E., Duran, J., Stinson, J., Lewis, M. A., & Zeltzer, L. (2013). Remote monitoring of pain and symptoms using wireless technology in children and adolescents with sickle cell disease. Journal of the American Association of Nurse Practitioners, 25(1), 42-54. https://dx.doi.org/10.1111/j.1745-7599.2012.00754.x

* Janna, I., Christopher, K., Barbara, S., Kalle, J., Joseph, K., Jari, L., & Katariina, S.-A. (2019). Science classroom activities and student situational engagement. International Journal of Science Education, 41(3), 316-329. https://dx.doi.org/10.1080/09500693.2018.1549372

Janssen, L. H. C., Elzinga, B. M., Verkuil, B., Hillegers, M. H. J., & Keijsers, L. (2021). The Link between Parental Support and Adolescent Negative Mood in Daily Life: between-Person Heterogeneity in within-Person Processes. Journal of Youth & Adolescence, 50(2), 271-285. https://dx.doi.org/10.1007/s10964-020-01323-w

Janssen, L. H. C., Kullberg, M. J., Verkuil, B., van Zwieten, N., Wever, M. C. M., van Houtum, L., Wentholt, W. G. M., & Elzinga, B. M. (2020). Does the COVID-19 pandemic impact parents' and adolescents' well-being? An EMA-study on daily affect and parenting. PLoS ONE [Electronic Resource], 15(10), e0240962. https://dx.doi.org/10.1371/journal.pone.0240962

Janssen, L. H. C., Sloan, C. J., Verkuil, B., Van Houtum, L., Wever, M. C. M., Fosco, G. M., & Elzinga, B. M. (2023). Adolescents' and parents' affect in relation to discrepant perceptions of parental warmth in daily life. Journal of Research on Adolescence, 09, 09. https://dx.doi.org/10.1111/jora.12879

* Janssen, L. H. C., Verkuil, B., van Houtum, L., Wever, M. C. M., & Elzinga, B. M. (2021). Perceptions of Parenting in Daily Life: Adolescent-Parent Differences and Associations with Adolescent Affect. Journal of Youth & Adolescence, 50(12), 2427-2443. https://dx.doi.org/10.1007/s10964-021-01489-x

Janssens, J. J., Achterhof, R., Lafit, G., Bamps, E., Hagemann, N., Hiekkaranta, A. P., Hermans, K., Lecei, A., Myin-Germeys, I., & Kirtley, O. J. (2021). The Impact of COVID-19 on Adolescents' Daily Lives: The Role of Parent-Child Relationship Quality. Journal of Research on Adolescence, 31(3), 623-644. https://dx.doi.org/10.1111/jora.12657

Janssens, J. J., Myin-Germeys, I., Lafit, G., Achterhof, R., Hagemann, N., Hermans, K., Hiekkaranta, A. P., Lecei, A., & Kirtley, O. J. (2023). Lifetime and Current Self-Harm Thoughts and Behaviors and Their Relationship to Parent and Peer Attachment.

Crisis: Journal of Crisis Intervention & Suicide, 44(5), 424-432. https://dx.doi.org/10.1027/0227-5910/a000878

Jarvinen, J., Ketonen, E. E., Hietajarvi, L., & Salmela-Aro, K. (2022). From high peaks to deep valleys: Using a situation- and person-oriented approach to assess within- and between-student variation in momentary engagement and disengagement. Learning and Instruction, 82, 1-11. https://dx.doi.org/10.1016/j.learninstruc.2022.101685

Jennen, L., Mazereel, V., Vansteelandt, K., Menne-Lothmann, C., Decoster, J., Derom, C., Thiery, E., Rutten, B. P. F., Jacobs, N., van Os, J., Wichers, M., De Hert, M., Vancampfort, D., & van Winkel, R. (2023). The within-person bidirectional association between physical activity and loneliness in the daily lives of adolescents and young adults. Mental Health and Physical Activity, 24. 10.1016/j.mhpa.2022.100499

* Jensen, M., George, M., Russell, M., & Odgers, C. (2019). Young Adolescents' Digital Technology Use and Mental Health Symptoms: Little Evidence of Longitudinal or Daily Linkages. Clinical Psychological Science, 7(6), 1416-1433. https://dx.doi.org/10.1177/2167702619859336

Jensen, M., George, M. J., Russell, M. A., Lippold, M. A., & Odgers, C. L. (2021). Daily Parent-Adolescent Digital Exchanges. Research on Child and Adolescent Psychopathology, 49(9), 1125-1138. https://dx.doi.org/10.1007/s10802-020-00765-x

Jensen, M., George, M. J., Russell, M. A., Lippold, M. A., & Odgers, C. L. (2021). Does Adolescent Digital Technology Use Detract from the Parent-Adolescent Relationship? Journal of Research on Adolescence, 31(2), 469-481. https://dx.doi.org/10.1111/jora.12618

* Jessup, G., Bundy, A. C., Broom, A., & Hancock, N. (2017). The Social Experiences of High School Students with Visual Impairments. Journal of Visual Impairment & Blindness, 111(1), 5-19. 10.1177/0145482X1711100102

Jibb, L. (2016). User-Centered Design Approach to the Development and Pilot Testing of a Smartphone App to Support Real-Time Pain Management for Adolescents with Cancer. User-Centered Design Approach to the Development & Pilot Testing of a Smartphone App to Support Real-Time Pain Management for Adolescents with Cancer, 1-1.

* Jibb, L. A., Stevens, B. J., Nathan, P. C., Seto, E., Cafazzo, J. A., Johnston, D. L., Hum, V., & Stinson, J. N. (2017). Implementation and preliminary effectiveness of a real-time pain management smartphone app for adolescents with cancer: A multicenter pilot clinical study. Pediatric Blood & Cancer, 64(10). https://dx.doi.org/10.1002/pbc.26554

* Johnson, E. I., & Swendsen, J. D. (2015). Perceived social status and early adolescents' responses to negative daily events. Journal of Child and Family Studies, 24(6), 1593-1604. https://dx.doi.org/10.1007/s10826-014-9963-y

* Juuti, K., Loukomies, A., & Lavonen, J. (2020). Interest in Dialogic and Non-Dialogic Teacher Talk Situations in Middle School Science Classroom. International Journal of Science and Mathematics Education, 18(8), 1531-1546. 10.1007/s10763-019-10031-2

Kapur, K., Li, X., Blood, E. A., & Hedeker, D. (2015). Bayesian mixed-effects location and scale models for multivariate longitudinal outcomes: an application to ecological momentary assessment data. Statistics in Medicine, 34(4), 630-651. https://dx.doi.org/10.1002/sim.6345

* Karsay, K., Matthes, J., Schmuck, D., & Ecklebe, S. (2023). Messaging, Posting, and Browsing: A Mobile Experience Sampling Study Investigating Youth's Social Media Use, Affective Well-Being, and Loneliness. Social Science Computer Review, 21, Article 08944393211058308. 10.1177/08944393211058308

* Kauer, S., Reid, S., Crooke, A. H. D., Khor, A., Hearps, S., Jorm, A. F., Sanci, L., & Patton, G. C. (2012). Self-monitoring Using Mobile Phones in the Early Stages of Adolescent Depression: Randomized Controlled Trial. Journal of Medical Internet Research, 14(3), e67-NA. 10.2196/jmir.1858

Kauer, S., Reid, S., Sanci, L., & Patton, G. (2006). Adolescent alcohol use and mobile phone experience sampling in a clinical setting: an innovative, youth friendly approach to research. Acta Neuropsychiatrica, 18(6), 255-256. https://dx.doi.org/10.1017/S0924270800030404

Kauer, S. D., Reid, S. C., Sanci, L., & Patton, G. C. (2009). Investigating the utility of mobile phones for collecting data about adolescent alcohol use and related mood, stress and coping behaviours: lessons and recommendations. Drug & Alcohol Review, 28(1), 25-30. https://dx.doi.org/10.1111/j.1465-3362.2008.00002.x

Kaurin, A., Do, Q. B., Ladouceur, C. D., Silk, J. S., & Wright, A. G. C. (2023). Daily manifestations of caregiver- and self-reported maladaptive personality traits in adolescent girls. Personality Disorders: Theory, Research, & Treatment, 14(5), 490-500. https://dx.doi.org/10.1037/per0000625

Kaurin, A., Sequeira, S. L., Ladouceur, C. D., McKone, K. M. P., Rosen, D., Jones, N., Wright, A. G. C., & Silk, J. S. (2022). Modeling sensitivity to social threat in adolescent girls: A psychoneurometric approach. Journal of Psychopathology and Clinical Science, 131(6), 641-652. https://dx.doi.org/10.1037/abn0000532

* Kennedy, T. M., Molina, B. S. G., & Pedersen, S. L. (2022). Change in Adolescents' Perceived ADHD Symptoms Across 17 Days of Ecological Momentary Assessment. Journal of Clinical Child and Adolescent Psychology, 1-16. https://dx.doi.org/10.1080/15374416.2022.2096043

* Ketonen, E. E., Salonen, V., Lonka, K., & Salmela-Aro, K. (2023). Can you feel the excitement? Physiological correlates of students' self-reported emotions. British

Journal of Educational Psychology, 93 Suppl 1, 113-129. https://dx.doi.org/10.1111/bjep.12534

* Khor, A. S., Gray, K. M., Reid, S. C., & Melvin, G. A. (2014). Feasibility and validity of ecological momentary assessment in adolescents with high-functioning autism and Asperger's disorder. Journal of Adolescence, 37(1), 37-46. https://dx.doi.org/10.1016/j.adolescence.2013.10.005

Khor, A. S., Melvin, G. A., Reid, S. C., & Gray, K. M. (2014). Coping, daily hassles and behavior and emotional problems in adolescents with high-functioning autism/Asperger's Disorder. Journal of Autism and Developmental Disorders, 44(3), 593-608. https://dx.doi.org/10.1007/s10803-013-1912-x

Kichline, T., Cushing, C. C., Connelly, M., Black, W. R., Simons, L. E., Friesen, C., & Schurman, J. V. (2022). Microtemporal Relationships in the Fear Avoidance Model: An Ecological Momentary Assessment Study. Clinical Journal of Pain, 38(9), 562-567. https://dx.doi.org/10.1097/AJP.0000000000001058

* Kichline, T., Cushing, C. C., Ortega, A., Friesen, C., & Schurman, J. V. (2019). Associations between Physical Activity and Chronic Pain Severity in Youth with Chronic Abdominal Pain. Clinical Journal of Pain, 35(7), 618-624. 10.1097/AJP.0000000000000716

* Kim, B. H., Ranzenhofer, L., Stadterman, J., Karvay, Y. G., & Burke, N. L. (2021). Food Insecurity and Eating Pathology in Adolescents. International Journal of Environmental Research & Public Health [Electronic Resource], 18(17), 30. https://dx.doi.org/10.3390/ijerph18179155

* Kim, J., Nakamura, T., Kikuchi, H., Sasaki, T., & Yamamoto, Y. (2013). Co-variation of depressive mood and locomotor dynamics evaluated by ecological momentary assessment in healthy humans. PLoS ONE [Electronic Resource], 8(9), e74979. https://dx.doi.org/10.1371/journal.pone.0074979

* King, K. M., Feil, M. C., & Halvorson, M. A. (2018). Negative urgency is correlated with the use of reflexive and disengagement emotion regulation strategies. Clinical Psychological Science, 6(6), 822-834. https://dx.doi.org/10.1177/2167702618785619

King, K. M., Halvorson, M. A., Kuehn, K. S., Feil, M. C., & Lengua, L. J. (2022). Cross-Study, Cross-Method Associations Between Negative Urgency and Internalizing Symptoms. Assessment, 29(3), 572-582. https://dx.doi.org/10.1177/1073191120983889

Kirchner, T., Magallon-Neri, E., Ortiz, M. S., Planellas, I., Forns, M., & Calderon, C. (2017). Adolescents' Daily Perception of Internalizing Emotional States by Means of Smartphone-based Ecological Momentary Assessment. Spanish Journal of Psychology, 20, E71. https://dx.doi.org/10.1017/sjp.2017.70

* Kirshenbaum, J. S., Coury, S. M., Colich, N. L., Manber, R., & Gotlib, I. H. (2023). Objective and subjective sleep health in adolescence: Associations with puberty and affect. Journal of Sleep Research, 32(3), e13805. https://dx.doi.org/10.1111/jsr.13805

Kirtley, O. J., Lafit, G., Vaessen, T., Decoster, J., Derom, C., Guloksuz, S., De Hert, M., Jacobs, N., Menne-Lothmann, C., Rutten, B. P. F., Thiery, E., van Os, J., van Winkel, R., Wichers, M., & Myin-Germeys, I. (2022). The relationship between daily positive future thinking and past-week suicidal ideation in youth: An experience sampling study. Frontiers in psychiatry Frontiers Research Foundation, 13, 915007. https://dx.doi.org/10.3389/fpsyt.2022.915007

Klaver, S. J., Dvorak, R. D., De Leon, A. N., Burr, E. K., Leary, A. V., Hayden, E. R., Peterson, R., Allen, Q., & Gwaltney, C. J. (2023). Support for incentive-sensitization theory in adolescent ad libitum smokers using ecological momentary assessment. Experimental and Clinical Psychopharmacology, 29, 29. https://dx.doi.org/10.1037/pha0000669

* Klipker, K., Wrzus, C., Rauers, A., Boker, S. M., & Riediger, M. (2017). Within-person changes in salivary testosterone and physical characteristics of puberty predict boys' daily affect. Hormones and Behavior, 95, 22-32. https://dx.doi.org/10.1016/j.yhbeh.2017.07.012

Klipker, K., Wrzus, C., Rauers, A., & Riediger, M. (2017). Hedonic orientation moderates the association between cognitive control and affect reactivity to daily hassles in adolescent boys. Emotion, 17(3), 497-508. https://dx.doi.org/10.1037/emo0000241

Koch, E. D., Tost, H., Braun, U., Gan, G., Giurgiu, M., Reinhard, I., Zipf, A., Meyer-Lindenberg, A., Ebner-Priemer, U. W., & Reichert, M. (2018). Mood Dimensions Show Distinct Within-Subject Associations With Non-exercise Activity in Adolescents: An Ambulatory Assessment Study. Frontiers in Psychology, 9, 268. https://dx.doi.org/10.3389/fpsyg.2018.00268

Koch, E. D., Tost, H., Braun, U., Gan, G., Giurgiu, M., Reinhard, I., Zipf, A., Meyer-Lindenberg, A., Ebner-Priemer, U. W., & Reichert, M. (2020). Relationships between incidental physical activity, exercise, and sports with subsequent mood in adolescents. Scandinavian Journal of Medicine and Science in Sports, 30(11), 2234-2250. https://dx.doi.org/10.1111/sms.13774

* Koechlin, H., Beeckman, M., Meier, A. H., Locher, C., Goubert, L., Kossowsky, J., & Simons, L. E. (2021). Association of parental and adolescent emotion-related factors with adolescent chronic pain behaviors. Pain, 12, 12. https://dx.doi.org/10.1097/j.pain.0000000000002508

* Koenig, J., Klier, J., Parzer, P., Santangelo, P., Resch, F., Ebner-Priemer, U., & Kaess, M. (2021). High-frequency ecological momentary assessment of emotional and interpersonal states preceding and following self-injury in female adolescents. European Child and Adolescent Psychiatry, 30(8), 1299-1308. https://dx.doi.org/10.1007/s00787-020-01626-0

* Kolar, D. R., Hammerle, F., Jenetzky, E., Huss, M., & Burger, A. (2016). Aversive tension in female adolescents with Anorexia Nervosa: a controlled ecological momentary assessment using smartphones. BMC Psychiatry, 16, 97. https://dx.doi.org/10.1186/s12888-016-0807-8

Kolar, D. R., Huss, M., Preuss, H. M., Jenetzky, E., Haynos, A. F., Burger, A., & Hammerle, F. (2017). Momentary emotion identification in female adolescents with and without anorexia nervosa. Psychiatry Research, 255, 394-398. https://dx.doi.org/10.1016/j.psychres.2017.06.075

Kolar, D. R., Neumayr, C., Roth, M., Voderholzer, U., Perthes, K., & Schlegl, S. (2020). Testing an emotion regulation model of physical activity in adolescents with anorexia nervosa: A pilot ecological momentary assessment. European Eating Disorders Review, 28(2), 170-183. https://dx.doi.org/10.1002/erv.2706

Konen, T., Dirk, J., Leonhardt, A., & Schmiedek, F. (2016). The interplay between sleep behavior and affect in elementary school children's daily life. Journal of Experimental Child Psychology, 150, 1-15. https://dx.doi.org/10.1016/j.jecp.2016.04.003

Konen, T., Dirk, J., & Schmiedek, F. (2015). Cognitive benefits of last night's sleep: daily variations in children's sleep behavior are related to working memory fluctuations. Journal of Child Psychology and Psychiatry and Allied Disciplines, 56(2), 171-182. https://dx.doi.org/10.1111/jcpp.12296

* Kovac, M., Mosner, M., Miller, S., Hanna, E. K., & Dichter, G. S. (2016). Experience Sampling of Positive Affect in Adolescents with Autism: Feasibility and Preliminary Findings. Research in Autism Spectrum Disorders, 29-30, 57-65. https://dx.doi.org/10.1016/j.rasd.2016.06.003

Kovalchik, S. A., Martino, S. C., Collins, R. L., Shadel, W. G., D'Amico, E. J., & Becker, K. (2018). Scaled inverse probability weighting: A method to assess potential bias due to event nonreporting in ecological momentary assessment studies. Journal of Educational and Behavioral Statistics, 43(3), 354-381. https://dx.doi.org/10.3102/1076998617738241

* Kracht, C. L., Beyl, R. A., Maher, J. P., Katzmarzyk, P. T., & Staiano, A. E. (2021). Adolescents' sedentary time, affect, and contextual factors: An ecological momentary assessment study. International Journal of Behavioral Nutrition & Physical Activity, 18(1), 53. https://dx.doi.org/10.1186/s12966-021-01121-y

Kramer, A. C., Neubauer, A. B., Leonhardt, A., Brose, A., Dirk, J., & Schmiedek, F. (2021). Ambulatory assessment of rumination and worry: Capturing perseverative cognitions in children's daily life. Psychological Assessment, 33(9), 827-842. https://dx.doi.org/10.1037/pas0001020

* Kubiak, T., Vogele, C., Siering, M., Schiel, R., & Weber, H. (2008). Daily hassles and emotional eating in obese adolescents under restricted dietary conditions--the role

of ruminative thinking. Appetite, 51(1), 206-209. https://dx.doi.org/10.1016/j.appet.2008.01.008

* Kudinova, A., Brick, L. A., Barthelemy, C., MacPherson, H. A., Jenkins, G., DeYoung, L., Gilbert, A., Radoeva, P., Kim, K., Armey, M., & Dickstein, D. (2022). Sex and age moderate the trajectory of guilt among children and adolescents with and without recent suicidal ideation. Cognition & Emotion, 1-15. https://dx.doi.org/10.1080/02699931.2022.2029359

Kudinova, A. Y., Brick, L. A., Armey, M., & Nugent, N. R. (2023). Micro-sequences of anger and shame and non-suicidal self-injury in youth: an ecological momentary assessment study. Journal of Child Psychology and Psychiatry and Allied Disciplines, 31, 31. https://dx.doi.org/10.1111/jcpp.13869

Kuhnhausen, J., Leonhardt, A., Dirk, J., & Schmiedek, F. (2013). Physical activity and affect in elementary school children's daily lives. Frontiers in Psychology, 4, 8, Article 456. 10.3389/fpsyg.2013.00456

Kuranova, A., Wigman, J. T. W., Menne-Lothmann, C., Decoster, J., van Winkel, R., Delespaul, P., Drukker, M., de Hert, M., Derom, C., Thiery, E., Rutten, B. P. F., Jacobs, N., van Os, J., Oldehinkel, A. J., Booij, S. H., & Wichers, M. (2021). Network dynamics of momentary affect states and future course of psychopathology in adolescents. PLoS ONE [Electronic Resource], 16(3), e0247458. https://dx.doi.org/10.1371/journal.pone.0247458

* Laakso, M., Fagerlund, Å., Pesonen, A.-K., Figueiredo, R. A. O., & Eriksson, J. G. (2023). The Impact of the Positive Education Program Flourishing Students on Early Adolescents' Daily Positive and Negative Emotions Using the Experience Sampling Method. Journal of Early Adolescence, 43(4), 385-417. 10.1177/02724316221105582

* Lavonen, J., Avalos, B., Upadyaya, K., Araneda, S., Juuti, K., Cumsille, P., Inkinen, J., & Salmela-Aro, K. (2021). Upper secondary students' situational interest in physics learning in Finland and Chile. International Journal of Science Education, 43(16), 2577-2596. 10.1080/09500693.2021.1978011

Lee, A. Y., Reynolds, K. D., Stacy, A., Niu, Z., & Xie, B. (2019). Family functioning, moods, and binge eating among urban adolescents. Journal of Behavioral Medicine, 42(3), 511-521. https://dx.doi.org/10.1007/s10865-018-9994-8

* Lee, M., & Bong, M. (2021). Patterns of motivation and learning during test-preparation of korean adolescents: An experience sampling approach. Educational Psychology, No Pagination Specified. https://dx.doi.org/10.1080/01443410.2021.2016634

* Lekkas, D., Gyorda, J. A., & Jacobson, N. C. (2023). A machine learning investigation into the temporal dynamics of physical activity-mediated emotional regulation in adolescents with anorexia nervosa and healthy controls. European Eating Disorders Review, 31(1), 147-165. https://dx.doi.org/10.1002/erv.2949

Lennarz, H. K., Hollenstein, T., Lichtwarck-Aschoff, A., Kuntsche, E., & Granic, I. (2019). Emotion regulation in action: Use, selection, and success of emotion regulation in adolescents' daily lives. International Journal of Behavioral Development, 43(1), 1-11. https://dx.doi.org/10.1177/0165025418755540

* Lennarz, H. K., Lichtwarck-Aschoff, A., Finkenauer, C., & Granic, I. (2017). Jealousy in adolescents' daily lives: How does it relate to interpersonal context and well-being? Journal of Adolescence, 54, 18-31. https://dx.doi.org/10.1016/j.adolescence.2016.09.008

Lennarz, H. K., Lichtwarck-Aschoff, A., Timmerman, M. E., & Granic, I. (2018). Emotion differentiation and its relation with emotional well-being in adolescents. Cognition & Emotion, 32(3), 651-657. https://dx.doi.org/10.1080/02699931.2017.1338177

Lennarz, H. K., van Roekel, E., Kuntsche, E., Lichtwarck-Aschoff, A., Hollenstein, T., Engels, R. C., & Granic, I. (2016). Associations between interpersonal relationships and negative affect in adolescents: An experience sampling study on the role of trait coping. Swiss Journal of Psychology, 75(2), 71-79. https://dx.doi.org/10.1024/1421-0185/a000172

* Letkiewicz, A. M., Funkhouser, C. J., Umemoto, A., Trivedi, E., Sritharan, A., Zhang, E., Buchanan, S. N., Helgren, F., Allison, G. O., Kayser, J., Shankman, S. A., & Auerbach, R. P. (2023). Neurophysiological responses to emotional faces predict dynamic fluctuations in affect in adolescents. Psychophysiology, e14476. https://dx.doi.org/10.1111/psyp.14476

* Lewandowski, L., Rieger, B., Smyth, J., Perry, L., & Gathje, R. (2009). Measuring post-concussion symptoms in adolescents: feasibility of ecological momentary assessment. Archives of Clinical Neuropsychology, 24(8), 791-796. https://dx.doi.org/10.1093/arclin/acp087

Li, X., & Hedeker, D. (2012). A three-level mixed-effects location scale model with an application to ecological momentary assessment data. Statistics in Medicine, 31(26), 3192-3210. https://dx.doi.org/10.1002/sim.5393

Liao, Y., Intille, S., Wolch, J., Pentz, M. A., & Dunton, G. F. (2014). Understanding the physical and social contexts of children's nonschool sedentary behavior: an ecological momentary assessment study. Journal of Physical Activity & Health, 11(3), 588-595. https://dx.doi.org/10.1123/jpah.2011-0363

Lim, V. H. T., Chen, Y. R., Tseng, M. H., Bundy, A., & Cordier, R. (2021). The impact of caregiver stigma on real-life social experience of Taiwanese adolescents with autism spectrum disorder. Autism, 25(7), 1859-1871. https://dx.doi.org/10.1177/13623613211004329

* Limberger, M. F., Schmiedek, F., Santangelo, P. S., Reichert, M., Wieland, L. M., Berhe, O., Meyer-Lindenberg, A., Tost, H., & Ebner-Priemer, U. W. (2023). Assessing affect in adolescents with e-diaries: multilevel confirmatory factor analyses of different factor

models. Frontiers in Psychology, 14, 1061229. https://dx.doi.org/10.3389/fpsyg.2023.1061229

Lin, X., Mermelstein, R., & Hedeker, D. (2018). A shared parameter location scale mixed effect model for EMA data subject to informative missing. Health Services and Outcomes Research Methodology, 18(4), 227-243. 10.1007/s10742-018-0184-5

Lin, X., Mermelstein, R., & Hedeker, D. (2020). Mixed location scale hidden Markov model for the analysis of intensive longitudinal data. Health Services and Outcomes Research Methodology, 20(4), 222-236. 10.1007/s10742-020-00217-5

Lin, X., Mermelstein, R. J., & Hedeker, D. (2018). A 3-level Bayesian mixed effects location scale model with an application to ecological momentary assessment data. Statistics in Medicine, 37(13), 2108-2119. https://dx.doi.org/10.1002/sim.7627

Lin, X., & Xun, X. (2021). Multivariate Shared-Parameter Mixed-Effects Location Scale Model for Analysis of Intensive Longitudinal Data. Statistics in Biopharmaceutical Research, 13(2), 230-238. 10.1080/19466315.2020.1828160

* Lionarons, J. M., Delespaul, P., Hellebrekers, D. M. J., Broen, M. P. G., Klinkenberg, S., Faber, C. G., Hendriksen, J. G. M., & Vles, J. S. H. (2023). Use of the experience sampling method in adolescents with Duchenne muscular dystrophy: a feasibility study. European Child and Adolescent Psychiatry, 01, 01. https://dx.doi.org/10.1007/s00787-023-02317-2

Lipperman-Kreda, S., Finan, L. J., & Grube, J. W. (2018). Social and situational characteristics associated with adolescents' drinking at party and non-party events. Addictive Behaviors, 83, 148-153. https://dx.doi.org/10.1016/j.addbeh.2017.12.001

Lipperman-Kreda, S., Gruenewald, P. J., Grube, J. W., & Bersamin, M. (2017). Adolescents, alcohol, and marijuana: Context characteristics and problems associated with simultaneous use. Drug and Alcohol Dependence, 179, 55-60. https://dx.doi.org/10.1016/j.drugalcdep.2017.06.023

* Lipperman-Kreda, S., Mair, C., & Gruenewald, P. J. (2021). Ecological momentary assessments of night-time drinking among California adolescents: bases for informing night-time preventive interventions. Addiction, 116(12), 3408-3421. https://dx.doi.org/10.1111/add.15623

Lopez, N. V., Lai, M. H., Yang, C. H., Dunton, G. F., & Belcher, B. R. (2022). Associations of Maternal and Paternal Parenting Practices With Children's Fruit and Vegetable Intake and Physical Activity: Preliminary Findings From an Ecological Momentary Study. JMIR Formative Research, 6(8), e38326. https://dx.doi.org/10.2196/38326

* Loukomies, A., Juuti, K., & Lavonen, J. (2015). Investigating situational interest in primary science lessons. International Journal of Science Education, 37(18), 3015-3037. https://dx.doi.org/10.1080/09500693.2015.1119909

Luijk, M., Bülow, A., Boele, S., de Haan, A., van der Kaap-deeder, J., & Keijsers, L. (2023). Overparenting in adolescents' everyday life: Development and validation of the momentary overparenting scale. Journal of Social and Personal Relationships, 19. 10.1177/02654075231192382

Magallon-Neri, E., Kirchner, T., Forns, M., Calderon, C., & Planellas, I. (2018). Ecological momentary assessment of contextual variables, satisfaction, and emotional and behavioral states of adolescents by level of victimization. Child Abuse and Neglect, 80, 268-276. https://dx.doi.org/10.1016/j.chiabu.2018.03.030

* Magallon-Neri, E., Kirchner-Nebot, T., Forns-Santacana, M., Calderon, C., & Planellas, I. (2016). Ecological Momentary Assessment with smartphones for measuring mental health problems in adolescents. World Journal of Psychiatry, 6(3), 303-310. https://dx.doi.org/10.5498/wjp.v6.i3.303

Maher, J. P., Dzubur, E., Nordgren, R., Huh, J., Chou, C. P., Hedeker, D., & Dunton, G. F. (2019). Do fluctuations in positive affective and physical feeling states predict physical activity and sedentary time? Psychology of Sport and Exercise, 41, 153-161. https://dx.doi.org/10.1016/j.psychsport.2018.01.011

* Malmberg, L. E., Walls, T. A., Martin, A. J., Little, T. D., & Lim, W. H. T. (2013). Primary school students' learning experiences of, and self-beliefs about competence, effort, and difficulty: Random effects models. Learning and Individual Differences, 28, 54-65. 10.1016/j.lindif.2013.09.007

Manasse, S. M., Haedt-Matt, A. A., Smith, K. E., Egbert, A. H., O'Sullivan, K., Koren, D., Engel, S., & Goldschmidt, A. B. (2022). The moderating role of sleep duration on momentary relations between negative affect and loss-of-control eating in children and adolescents. European Eating Disorders Review, 26, 26. https://dx.doi.org/10.1002/erv.2908

Manasse, S. M., Haedt-Matt, A. A., Smith, K. E., Egbert, A. H., O'Sullivan, K., Koren, D., Engel, S., & Goldschmidt, A. B. (2022). The moderating role of sleep duration on momentary relations between negative affect and loss-of-control eating in children and adolescents. European Eating Disorders Review, 30(6), 815-822. 10.1002/erv.2908

* Martin, A. J., Mansour, M., & Malmberg, L.-E. (2020). What factors influence students' real-time motivation and engagement? An experience sampling study of high school students using mobile technology. Educational Psychology, 40(9), 1113-1135. https://dx.doi.org/10.1080/01443410.2018.1545997

* Martin, A. J., Papworth, B., Ginns, P., Malmberg, L.-E., Collie, R. J., & Calvo, R. A. (2015). Real-time motivation and engagement during a month at school: Every moment of every day for every student matters. Learning and Individual Differences, 38, 26-35. https://dx.doi.org/10.1016/j.lindif.2015.01.014

Martino, S. C., Kovalchik, S. A., Collins, R. L., Becker, K. M., Shadel, W. G., & D'Amico, E. J. (2016). Ecological Momentary Assessment of the Association Between Exposure to

Alcohol Advertising and Early Adolescents' Beliefs About Alcohol. Journal of Adolescent Health, 58(1), 85-91. https://dx.doi.org/10.1016/j.jadohealth.2015.08.010

Martino, S. C., Setodji, C. M., Collins, R. L., D'Amico, E. J., Shadel, W. G., Tolpadi, A., & Becker, K. M. (2018). Persistence of Shifts in Beliefs Associated With Exposure to Alcohol Advertising Among Adolescents. Journal of Studies on Alcohol and Drugs, 79(3), 399-407. 10.15288/jsad.2018.79.399

* Maskevich, S., Shen, L., Drummond, S. P. A., & Bei, B. (2021). What time do you plan to sleep tonight? An intense longitudinal study of adolescent daily sleep self-regulation via planning and its associations with sleep opportunity. Journal of Child Psychology and Psychiatry and Allied Disciplines, 22, 22. https://dx.doi.org/10.1111/jcpp.13540

Maskevich, S., Shen, L., Drummond, S. P. A., & Bei, B. (2022). What time do you plan to sleep tonight? An intense longitudinal study of adolescent daily sleep self-regulation via planning and its associations with sleep opportunity. Journal of Child Psychology & Psychiatry, 63(8), 900-911. 10.1111/jcpp.13540

* Mason, M., Mennis, J., Way, T., & Floyd Campbell, L. (2015). Real-Time Readiness to Quit and Peer Smoking within a Text Message Intervention for Adolescent Smokers: Modeling Mechanisms of Change. Journal of Substance Abuse Treatment, 59, 67-73. https://dx.doi.org/10.1016/j.jsat.2015.07.009

Mason, M., Mennis, J., Way, T., Lanza, S., Russell, M., & Zaharakis, N. (2015). Time-varying effects of a text-based smoking cessation intervention for urban adolescents. Drug and Alcohol Dependence, 157, 99-105. https://dx.doi.org/10.1016/j.drugalcdep.2015.10.016

Mason, M. J., Mennis, J., Zaharakis, N. M., & Way, T. (2016). The Dynamic Role of Urban Neighborhood Effects in a Text-Messaging Adolescent Smoking Intervention. Nicotine & Tobacco Research, 18(5), 1039-1045. https://dx.doi.org/10.1093/ntr/ntv254

Mason, T. B., Naya, C. H., Schembre, S. M., Smith, K. E., & Dunton, G. F. (2020). Internalizing symptoms modulate real-world affective response to sweet food and drinks in children. Behaviour Research and Therapy, 135, 103753. https://dx.doi.org/10.1016/j.brat.2020.103753

Mason, T. B., O'Connor, S. G., Schembre, S. M., Huh, J., Chu, D., & Dunton, G. F. (2019). Momentary affect, stress coping, and food intake in mother-child dyads. Health Psychology, 38(3), 238-247. https://dx.doi.org/10.1037/hea0000714

Mason, T. B., Smith, K. E., & Dunton, G. F. (2020). Maternal parenting styles and ecological momentary assessment of maternal feeding practices and child food intake across middle childhood to early adolescence. Pediatric Obesity, 15(10), e12683. https://dx.doi.org/10.1111/ijpo.12683

* Mayne, S. L., DiFiore, G., Hannan, C., Nwokeji, U., Tam, V., Filograna, C., Martin, T., South, E., Mitchell, J. A., Glanz, K., & Fiks, A. G. (2023). Feasibility and acceptability of mobile methods to assess home and neighborhood environments related to adolescent sleep. Sleep Health, 9(3), 331-338. https://dx.doi.org/10.1016/j.sleh.2023.01.014

* Mazereel, V., Vansteelandt, K., Menne-Lothmann, C., Decoster, J., Derom, C., Thiery, E., Rutten, B. P. F., Jacobs, N., van Os, J., Wichers, M., De Hert, M., Vancampfort, D., & van Winkel, R. (2021). The complex and dynamic interplay between self-esteem, belongingness and physical activity in daily life: An experience sampling study in adolescence and young adulthood. Mental Health and Physical Activity, 21. 10.1016/j.mhpa.2021.100413

Mazereel, V., Vansteelandt, K., Menne-Lothmann, C., Decoster, J., Derom, C., Thiery, E., Rutten, B. P. F., Jacobs, N., van Os, J., Wichers, M., De Hert, M., Vancampfort, D., & van Winkel, R. (2023). Associations between childhood adversity, psychiatric symptoms, and self-esteem outcomes in adolescents and young adults: An experience sampling study. Journal of Clinical Psychology, 06, 06. https://dx.doi.org/10.1002/jclp.23599

McKee, K., Russell, M., Mennis, J., Mason, M., & Neale, M. (2020). Emotion regulation dynamics predict substance use in high-risk adolescents. Addictive Behaviors Vol 106 2020, ArtID 106374, 106. https://dx.doi.org/10.1016/j.addbeh.2020.106374

McKone, K. M. P., Edershile, E. A., Ladouceur, C. D., & Silk, J. S. (2022). Real-world flexibility in adolescent girls' emotion regulation strategy selection: An investigation of strategy switching. Development and Psychopathology, 1-15. https://dx.doi.org/10.1017/S0954579422001079

Meier, A., Beyens, I., Siebers, T., Pouwels, J. L., & Valkenburg, P. M. (2023). Habitual social media and smartphone use are linked to task delay for some, but not all, adolescents. Journal of Computer-Mediated Communication, 28(3), 12, Article zmad008. 10.1093/jcmc/zmad008

* Mennis, J., Mason, M., & Ambrus, A. (2018). Urban Greenspace is Associated with Reduced Psychological Stress among Adolescents: A Geographic Ecological Momentary Assessment (GEMA) Analysis of Activity Space. Landscape & Urban Planning, 174, 1-9. https://dx.doi.org/10.1016/j.landurbplan.2018.02.008

Mennis, J., Mason, M., Ambrus, A., Way, T., & Henry, K. (2017). The spatial accuracy of geographic ecological momentary assessment (GEMA): Error and bias due to subject and environmental characteristics. Drug and Alcohol Dependence, 178, 188-193. https://dx.doi.org/10.1016/j.drugalcdep.2017.05.019

Mennis, J., Mason, M., Coffman, D. L., & Henry, K. (2018). Geographic Imputation of Missing Activity Space Data from Ecological Momentary Assessment (EMA) GPS Positions. International Journal of Environmental Research & Public Health [Electronic Resource], 15(12), 04. https://dx.doi.org/10.3390/ijerph15122740

Mennis, J., Mason, M., Light, J., Rusby, J., Westling, E., Way, T., Zahakaris, N., & Flay, B. (2016). Does substance use moderate the association of neighborhood disadvantage with perceived stress and safety in the activity spaces of urban youth? Drug and Alcohol Dependence, 165, 288-292. https://dx.doi.org/10.1016/j.drugalcdep.2016.06.019

Mens, M. M. J., Keijsers, L., Dietvorst, E., Koval, S., Legerstee, J. S., & Hillegers, M. H. J. (2022). Promoting Daily Well-being in Adolescents using mHealth. Journal of Youth & Adolescence, 51(11), 2173-2189. https://dx.doi.org/10.1007/s10964-022-01656-8

* Mereish, E. H., Treloar Padovano, H., Parlette, B., & Miranda, R., Jr. (2022). Momentary Associations Among Minority Stress, Craving, Affect, and Nicotine Use Among Sexual Minority Youth. Journal of Clinical Child and Adolescent Psychology, 51(6), 877-891. https://dx.doi.org/10.1080/15374416.2022.2093208

* Messer, L. H., Vigers, T., Pyle, L., Fivekiller, E., Wadwa, R. P., Hernandez, T. L., & Cook, P. F. (2022). Novel predictors of daily fluctuations in glycemia and self-management in adolescents and young adults with type 1 diabetes. Diabetic Medicine, 39(9). 10.1111/dme.14910

* Michel, C., Lerch, S., Buetiger, J. R., Fluckiger, R., Cavelti, M., Koenig, J., Kaess, M., & Kindler, J. (2023). An ecological momentary assessment study of age effects on perceptive and non-perceptive clinical high-risk symptoms of psychosis. European Child and Adolescent Psychiatry, 32(10), 1841-1852. https://dx.doi.org/10.1007/s00787-022-02003-9

* Mikalsen, I. B., Nassehi, D., & Oymar, K. (2019). Vortex Whistle and Smart Phone Application for Peak Flow Recordings in Asthmatic Children: A Feasibility Study. Telemedicine Journal and e-Health, 25(11), 1077-1082. https://dx.doi.org/10.1089/tmj.2018.0270

* Milojevich, H. M., Stickel, D., Swingler, M. M., Zhang, X., Terrell, J., Sheridan, M. A., & Tan, X. (2023). Building an ecological momentary assessment smartphone app for 4- to 10-year-old children: A pilot study. PLoS ONE [Electronic Resource], 18(8), e0290148. https://dx.doi.org/10.1371/journal.pone.0290148

* Minich, M., Zhao, Q., Eickhoff, J., & Moreno, M. A. (2023). In the Mood for Music: Listening to Music and Other Smartphone Uses Improve Adolescent Mood. Cyberpsychology, behavior and social networking, 10, 10. https://dx.doi.org/10.1089/cyber.2022.0344

Modecki, K. L., Duvenage, M., Uink, B., Barber, B. L., & Donovan, C. L. (2021). Adolescents' Online Coping: When Less Is More but None Is Worse. Clinical Psychological Science, 15, Article 21677026211028983. 10.1177/21677026211028983

Moeller, J., Brackett, M. A., Ivcevic, Z., & White, A. E. (2020). High school students' feelings: Discoveries from a large national survey and an experience sampling study. Learning and Instruction, 66, 15, Article 101301. 10.1016/j.learninstruc.2019.101301

* Moeller, J., Ivcevic, Z., Brackett, M. A., & White, A. E. (2018). Mixed emotions: Network analyses of intra-individual co-occurrences within and across situations. Emotion, 18(8), 1106-1121. https://dx.doi.org/10.1037/emo0000419

Moschko, T., Stadler, G., & Gawrilow, C. (2023). Fluctuations in children's self-regulation and parent-child interaction in everyday life: An ambulatory assessment study. Journal of Social & Personal Relationships, 40(1), 254-276. 10.1177/02654075221116788

* Muller, L. R. F., Gossmann, K., Schmid, R. F., Rosner, R., & Unterhitzenberger, J. (2021). A pilot study on ecological momentary assessment in asylum-seeking children and adolescents resettled to Germany: Investigating compliance, post-migration factors, and the relation between daily mood, sleep patterns, and mental health. PLoS ONE [Electronic Resource], 16(2), e0246069. https://dx.doi.org/10.1371/journal.pone.0246069

* Mulvaney, S. A., Rothman, R. L., Dietrich, M. S., Wallston, K. A., Grove, E., Elasy, T. A., & Johnson, K. B. (2012). Using mobile phones to measure adolescent diabetes adherence. Health Psychology, 31(1), 43-50. https://dx.doi.org/10.1037/a0025543

* Mulvaney, S. A., Vaala, S., Hood, K. K., Lybarger, C., Carroll, R., Williams, L., Schmidt, D. C., Johnson, K., Dietrich, M. S., & Laffel, L. (2018). Mobile Momentary Assessment and Biobehavioral Feedback for Adolescents with Type 1 Diabetes: Feasibility and Engagement Patterns. Diabetes Technology & Therapeutics, 20(7), 465-474. https://dx.doi.org/10.1089/dia.2018.0064

Mulvaney, S. A., Vaala, S. E., Carroll, R. B., Williams, L. K., Lybarger, C. K., Schmidt, D. C., Dietrich, M. S., Laffel, L. M., & Hood, K. K. (2019). A mobile app identifies momentary psychosocial and contextual factors related to mealtime self-management in adolescents with type 1 diabetes. Journal of the American Medical Informatics Association, 26(12), 1627-1631. https://dx.doi.org/10.1093/jamia/ocz147

* Murray, D. W., Ridenour, T. A., Swingler, M. M., Morgan, A., & Hegarty-Craver, M. (2023). Feasibility of combining biosensor and ecological momentary assessment to measure stress experiences among economically disadvantaged adolescents. Stress & Health, 39(3), 684-689. https://dx.doi.org/10.1002/smi.3211

Murray, L., Israel, E. S., Balkind, E. G., Pastro, B., Lovell-Smith, N., Lukas, S. E., Forbes, E. E., Pizzagalli, D. A., & Webb, C. A. (2023). Multi-modal assessment of reward functioning in adolescent anhedonia. Psychological Medicine, 53(10), 4424-4433. https://dx.doi.org/10.1017/S0033291722001222

Naim, R., Shaughnessy, S., Smith, A., Karalunas, S. L., Kircanski, K., & Brotman, M. A. (2022). Real-time assessment of positive and negative affective fluctuations and mood lability in a transdiagnostic sample of youth. Depression and Anxiety, 39(12), 870-880. https://dx.doi.org/10.1002/da.23293

* Naim, R., Smith, A., Chue, A., Grassie, H., Linke, J., Dombek, K., Shaughnessy, S., McNeil, C., Cardinale, E., Agorsor, C., Cardenas, S., Brooks, J., Subar, A. R., Jones, E. L., Do, Q. B.,

Pine, D. S., Leibenluft, E., Brotman, M. A., & Kircanski, K. (2021). Using ecological momentary assessment to enhance irritability phenotyping in a transdiagnostic sample of youth. Development and Psychopathology, 33(5), 1734-1746, Article Pii s0954579421000717. 10.1017/s0954579421000717

* Nap-van der Vlist, M. M., Houtveen, J., Dalmeijer, G. W., Grootenhuis, M. A., van der Ent, C. K., van Grotel, M., Swart, J. F., van Montfrans, J. M., van de Putte, E. M., & Nijhof, S. L. (2021). Internet and smartphone-based ecological momentary assessment and personalized advice (PROfeel) in adolescents with chronic conditions: A feasibility study. Internet Interventions, 25, 100395. https://dx.doi.org/10.1016/j.invent.2021.100395

Naya, C. H., Chu, D., Wang, W. L., Nicolo, M., Dunton, G. F., & Mason, T. B. (2022). Children's Daily Negative Affect Patterns and Food Consumption on Weekends: An Ecological Momentary Assessment Study. Journal of Nutrition Education and Behavior, 54(7), 600-609. https://dx.doi.org/10.1016/j.jneb.2022.02.007

Naya, C. H., Zink, J., Huh, J., Dunton, G. F., & Belcher, B. R. (2021). Examining the same-day relationship between morning cortisol after awakening, perceived stress in the morning, and physical activity in youth. Stress, 24(3), 338-347. https://dx.doi.org/10.1080/10253890.2020.1804852

* Nereim, C., Bickham, D., & Rich, M. (2022). Exploring Use Patterns and Racial and Ethnic Differences in Real Time Affective States During Social Media Use Among a Clinical Sample of Adolescents With Depression: Prospective Cohort Study. JMIR Formative Research, 6(5), e30900. https://dx.doi.org/10.2196/30900

Neubauer, A. B., Dirk, J., & Schmiedek, F. (2019). Momentary working memory performance is coupled with different dimensions of affect for different children: A mixture model analysis of ambulatory assessment data. Developmental Psychology, 55(4), 754-766. https://dx.doi.org/10.1037/dev0000668

* Neubauer, A. B., Kramer, A. C., Schmidt, A., Konen, T., Dirk, J., & Schmiedek, F. (2021). Reciprocal relations of subjective sleep quality and affective well-being in late childhood. Developmental Psychology, 57(8), 1372-1386. https://dx.doi.org/10.1037/dev0001209

Neubauer, A. B., Kramer, A. C., & Schmiedek, F. (2022). Assessing domain-general need fulfillment in children and adults: Introducing the General Need Satisfaction and Frustration scale. Psychological Assessment, 34(11), 1022-1035. https://dx.doi.org/10.1037/pas0001169

Neubauer, A. B., Schmidt, A., Schmiedek, F., & Dirk, J. (2022). Dynamic reciprocal relations of achievement goals with daily experiences of academic success and failure: An ambulatory assessment study*. Learning and Instruction, 81, 9, Article 101617. 10.1016/j.learninstruc.2022.101617

* Niestroj, S. C., Steden, S., Boecker, M., Brodkin, E. S., & Konrad, K. (2023). The Development and Validation of the First German Open Scale of Social Information Processing. Psychopathology, 56(1-2), 52-63. https://dx.doi.org/10.1159/000525950

* Nock, M. K., Prinstein, M. J., & Sterba, S. K. (2009). Revealing the form and function of self-injurious thoughts and behaviors: A real-time ecological assessment study among adolescents and young adults. Journal of Abnormal Psychology, 118(4), 816-827. https://dx.doi.org/10.1037/a0016948

* Nock, M. K., Prinstein, M. J., & Sterba, S. K. (2010). Revealing the form and function of self-injurious thoughts and behaviors: A real-time ecological assessment study among adolescents and young adults. Psychology of Violence, 1(S), 36-52. https://dx.doi.org/10.1037/2152-0828.1.S.36

* Noel, C., Armiento, C., Pefoyo, A. K., Klein, R., Bedard, M., & Scharf, D. (2021). Adolescent exposure to cannabis marketing following recreational cannabis legalization in Canada: A pilot study using ecological momentary assessment. Addictive Behaviors Reports, 14, 100383. https://dx.doi.org/10.1016/j.abrep.2021.100383

* Nollen, N. L., Mayo, M. S., Carlson, S. E., Rapoff, M. A., Goggin, K. J., & Ellerbeck, E. F. (2014). Mobile technology for obesity prevention: a randomized pilot study in racial- and ethnic-minority girls. American Journal of Preventive Medicine, 46(4), 404-408. https://dx.doi.org/10.1016/j.amepre.2013.12.011

Nook, E. C., Flournoy, J. C., Rodman, A. M., Mair, P., & McLaughlin, K. A. (2021). High emotion differentiation buffers against internalizing symptoms following exposure to stressful life events in adolescence: An intensive longitudinal study. Clinical Psychological Science, 9(4), 699-718. https://dx.doi.org/10.1177/2167702620979786

* Nugent, N. R., Armey, M., Boker, S., Brick, L., Knopik, V., McGeary, J. E., Spirito, A., & Mehl, M. R. (2022). Adolescents hospitalised for suicidality: biomarkers, social and affective predictors: a cohort study. BMJ Open, 12(10), e056063. https://dx.doi.org/10.1136/bmjopen-2021-056063

O'Connor, S. G., Ke, W., Dzubur, E., Schembre, S., & Dunton, G. F. (2018). Concordance and predictors of concordance of children's dietary intake as reported via ecological momentary assessment and 24 h recall. Public Health Nutrition, 21(6), 1019-1027. https://dx.doi.org/10.1017/S1368980017003780

O'Connor, S. G., Koprowski, C., Dzubur, E., Leventhal, A. M., Huh, J., & Dunton, G. F. (2017). Differences in Mothers' and Children's Dietary Intake during Physical and Sedentary Activities: An Ecological Momentary Assessment Study. Journal of the Academy of Nutrition and Dietetics, 117(8), 1265-1271. https://dx.doi.org/10.1016/j.jand.2017.02.012

Odgers, C. L., & Russell, M. A. (2017). Violence exposure is associated with adolescents' same- and next-day mental health symptoms. Journal of Child Psychology and

Psychiatry and Allied Disciplines, 58(12), 1310-1318. https://dx.doi.org/10.1111/jcpp.12763

O'Reilly, G. A., Huh, J., Schembre, S. M., Tate, E. B., Pentz, M. A., & Dunton, G. (2015). Association of usual self-reported dietary intake with ecological momentary measures of affective and physical feeling states in children. Appetite, 92, 314-321. https://dx.doi.org/10.1016/j.appet.2015.05.032

* Ortega-Williams, A., Booth, J. M., Fussell-Ware, D. J., Lawrence, Y. J., Pearl, D., Chapman, N., Allen, W., Reid-Moore, A., & Overby, Z. (2022). Using Ecological Momentary Assessments to Understand Black Youths' Experiences of Racism, Stress, and Safety. Journal of Research on Adolescence, 32(1), 270-289. https://dx.doi.org/10.1111/jora.12733

* Owens, J., Sangal, R. B., Sutton, V. K., Bakken, R., Allen, A. J., & Kelsey, D. (2009). Subjective and objective measures of sleep in children with attention-deficit/hyperactivity disorder. Sleep Medicine, 10(4), 446-456. https://dx.doi.org/10.1016/j.sleep.2008.03.013

Parker, M. N., LeMay-Russell, S., Schvey, N. A., Crosby, R. D., Ramirez, E., Kelly, N. R., Shank, L. M., Byrne, M. E., Engel, S. G., Swanson, T. N., Djan, K. G., Kwarteng, E. A., Faulkner, L. M., Zenno, A., Brady, S. M., Yanovski, S. Z., Tanofsky-Kraff, M., & Yanovski, J. A. (2022). Associations of sleep with food cravings and loss-of-control eating in youth: An ecological momentary assessment study. Pediatric Obesity, 17(2), e12851. https://dx.doi.org/10.1111/ijpo.12851

* Parker, M. N., Tanofsky-Kraff, M., Crosby, R. D., Byrne, M. E., LeMay-Russell, S., Swanson, T. N., Ramirez, E., Shank, L. M., Djan, K. G., Kwarteng, E. A., Faulkner, L. M., Yang, S. B., Zenno, A., Chivukula, K. K., Engel, S. G., Brady, S. M., Yanovski, S. Z., & Yanovski, J. A. (2021). Food cravings and loss-of-control eating in youth: Associations with gonadal hormone concentrations. International Journal of Eating Disorders, 54(8), 1426-1437. 10.1002/eat.23530

Parker, M. N., Tanofsky-Kraff, M., Crosby, R. D., Shank, L. M., Kwarteng, E. A., Loch, L. K., Faulkner, L. M., Haynes, H. E., Gupta, S., Fatima, S., Dzombak, J. W. P., Zenno, A., Engel, S. G., Brady, S. M., Yanovski, S. Z., & Yanovski, J. A. (2022). State negative affect in relation to loss-of-control eating among children and adolescents in the natural environment. Appetite, 178, 106166. https://dx.doi.org/10.1016/j.appet.2022.106166

Parnes, J. E., Mereish, E. H., Meisel, S. N., Treloar Padovano, H., & Miranda, R., Jr. (2023). In the Presence of Parents: Parental Heterosexism and Momentary Negative Affect and Substance Craving Among Sexual Minority Youth. Journal of Adolescent Health, 72(2), 230-236. https://dx.doi.org/10.1016/j.jadohealth.2022.09.029

Pauligk, S., Seidel, M., Furtjes, S., King, J. A., Geisler, D., Hellerhoff, I., Roessner, V., Schmidt, U., Goschke, T., Walter, H., Strobel, A., & Ehrlich, S. (2021). The costs of over-control in

anorexia nervosa: evidence from fMRI and ecological momentary assessment. Transl Psychiatry Psychiatry, 11(1), 304. https://dx.doi.org/10.1038/s41398-021-01405-8

Pedersen, S. L., Kennedy, T. M., Joseph, H. M., Riston, S. J., Kipp, H. L., & Molina, B. S. G. (2020). Real-World Changes in Adolescents' ADHD Symptoms within the Day and across School and Non-school Days. Journal of Abnormal Child Psychology, 48(12), 1543-1553. https://dx.doi.org/10.1007/s10802-020-00695-8

Peschel, S. K. V., Furtjes, S., Sigrist, C., Voss, C., Berwanger, J., Ollmann, T. M., Kische, H., Ruckert, F., Koenig, J., Pieper, L., & Beesdo-Baum, K. (2023). Tension and disordered eating behaviors in the daily lives of adolescents and young adults from the general population: Associations and moderating role of trait emotion regulation. Scandinavian Journal of Psychology, 18, 18. https://dx.doi.org/10.1111/sjop.12971

Peschel, S. K. V., Furtjes, S., Voss, C., Sigrist, C., Berwanger, J., Ollmann, T. M., Kische, H., Ruckert, F., Koenig, J., & Beesdo-Baum, K. (2023). Temporal associations between experiential avoidance and disordered eating behaviors in adolescents and young adults: findings from an epidemiological cohort study with ecological momentary assessment. Eating & Weight Disorders: EWD, 28(1), 58. https://dx.doi.org/10.1007/s40519-023-01584-x

Piasecki, T. M., Hedeker, D., Dierker, L. C., & Mermelstein, R. J. (2016). Progression of nicotine dependence, mood level, and mood variability in adolescent smokers. Psychology of Addictive Behaviors, 30(4), 484-493. https://dx.doi.org/10.1037/adb0000165

Piasecki, T. M., Trela, C. J., Hedeker, D., & Mermelstein, R. J. (2014). Smoking antecedents: separating between- and within-person effects of tobacco dependence in a multiwave ecological momentary assessment investigation of adolescent smoking. Nicotine & Tobacco Research, 16 Suppl 2, S119-126. https://dx.doi.org/10.1093/ntr/ntt132

* Pihet, S., De Ridder, J., & Suter, M. (2017). Ecological Momentary Assessment (EMA) goes to jail: Capturing daily antisocial behavior in its context, a feasibility and reliability study in incarcerated juvenile offenders. European Journal of Psychological Assessment, 33(2), 87-96. https://dx.doi.org/10.1027/1015-5759/a000275

Piontak, J. R., Russell, M. A., Danese, A., Copeland, W. E., Hoyle, R. H., & Odgers, C. L. (2017). Violence exposure and adolescents' same-day obesogenic behaviors: New findings and a replication. Social Science and Medicine, 189, 145-151. https://dx.doi.org/10.1016/j.socscimed.2017.07.004

Plessen, K. J., Constanty, L., Ranjbar, S., Turri, F., Miano, G., Lepage, C., & Urben, S. (2022). The role of self-regulatory control processes in understanding aggressive ideations and behaviors: An experience sampling method study. Frontiers in psychiatry Frontiers Research Foundation, 13, 1058814. https://dx.doi.org/10.3389/fpsyt.2022.1058814

Pouwels, J. L., Valkenburg, P. M., Beyens, I., van, D., II, & Keijsers, L. (2021). Social media use and friendship closeness in adolescents' daily lives: An experience sampling study. Developmental Psychology, 57(2), 309-323. https://dx.doi.org/10.1037/dev0001148

Pouwels, J. L., Valkenburg, P. M., Beyens, I., van, D., II, & Keijsers, L. (2021). Some socially poor but also some socially rich adolescents feel closer to their friends after using social media. Scientific Reports, 11(1), 21176. https://dx.doi.org/10.1038/s41598-021-99034-0

* Poysa, S., Vasalampi, K., Muotka, J., Lerkkanen, M. K., Poikkeus, A. M., & Nurmi, J. E. (2018). Variation in situation-specific engagement among lower secondary school students. Learning and Instruction, 53, 64-73. 10.1016/j.leaminstruc.2017.07.007

* Pramana, G., Parmanto, B., Kendall, P. C., & Silk, J. S. (2014). The SmartCAT: an m-health platform for ecological momentary intervention in child anxiety treatment. Telemedicine Journal and e-Health, 20(5), 419-427. https://dx.doi.org/10.1089/tmj.2013.0214

Pries, L. K., Klingenberg, B., Menne-Lothmann, C., Decoster, J., van Winkel, R., Collip, D., Delespaul, P., De Hert, M., Derom, C., Thiery, E., Jacobs, N., Wichers, M., Cinar, O., Lin, B. D., Luykx, J. J., Rutten, B. P. F., van Os, J., & Guloksuz, S. (2020). Polygenic liability for schizophrenia and childhood adversity influences daily-life emotion dysregulation and psychosis proneness. Acta Psychiatrica Scandinavica, 141(5), 465-475. https://dx.doi.org/10.1111/acps.13158

* Psihogios, A. M., Li, Y., Ahmed, A., Huang, J., Kersun, L. S., Schwartz, L. A., & Barakat, L. P. (2021). Daily text message assessments of 6-mercaptopurine adherence and its proximal contexts in adolescents and young adults with leukemia: A pilot study. Pediatric Blood & Cancer, 68(2), e28767. https://dx.doi.org/10.1002/pbc.28767

Pugach, O., Hedeker, D., & Mermelstein, R. (2014). A Bivariate Mixed-Effects Location-Scale Model with application to Ecological Momentary Assessment (EMA) data. Health Services & Outcomes Research Methodology, 14(4), 194-212.

Pugach, O., Hedeker, D., Richmond, M. J., Sokolovsky, A., & Mermelstein, R. (2014). Modeling mood variation and covariation among adolescent smokers: application of a bivariate location-scale mixed-effects model. Nicotine & Tobacco Research, 16 Suppl 2, S151-158. https://dx.doi.org/10.1093/ntr/ntt143

* Rabbitts, J. A., Holley, A. L., Karlson, C. W., & Palermo, T. M. (2014). Bidirectional associations between pain and physical activity in adolescents. Clinical Journal of Pain, 30(3), 251-258. https://dx.doi.org/10.1097/AJP.0b013e31829550c6

* Rabbitts, J. A., Zhou, C., Narayanan, A., & Palermo, T. M. (2017). Longitudinal and Temporal Associations Between Daily Pain and Sleep Patterns After Major Pediatric Surgery. Journal of Pain, 18(6), 656-663. https://dx.doi.org/10.1016/j.jpain.2017.01.004

* Rah, M. J., Walline, J. J., Lynn Mitchell, G., & Zadnik, K. (2006). Comparison of the experience sampling method and questionnaires to assess visual activities in pre-teen and adolescent children. Ophthalmic and Physiological Optics, 26(5), 483-489.

* Rahdar, A., & Galván, A. (2014). The cognitive and neurobiological effects of daily stress in adolescents. Neuroimage, 92, 267-273. 10.1016/j.neuroimage.2014.02.007

Ramirez, J., & Miranda, R., Jr. (2014). Alcohol craving in adolescents: bridging the laboratory and natural environment. Psychopharmacology, 231(8), 1841-1851. https://dx.doi.org/10.1007/s00213-013-3372-6

Randall, W. M., Baltazar, M., & Saarikallio, S. (2022). Success in reaching affect self-regulation goals through everyday music listening. Journal of New Music Research, 51(2-3), 243-258. 10.1080/09298215.2023.2187310

Ranzenhofer, L. M., Engel, S. G., Crosby, R. D., Anderson, M., Vannucci, A., Cohen, L. A., Cassidy, O., & Tanofsky-Kraff, M. (2014). Using ecological momentary assessment to examine interpersonal and affective predictors of loss of control eating in adolescent girls. International Journal of Eating Disorders, 47(7), 748-757. https://dx.doi.org/10.1002/eat.22333

* Ranzenhofer, L. M., Engel, S. G., Crosby, R. D., Haigney, M., & Tanofsky-Kraff, M. (2016). A pilot study of attachment style and emotional experience in adolescent girls with loss of control eating. Advances in Eating Disorders, 4(3), 250-263. https://dx.doi.org/10.1080/21662630.2016.1227273

Ranzenhofer, L. M., Engel, S. G., Crosby, R. D., Haigney, M. C., Anderson, M., McCaffery, J. M., & Tanofsky-Kraff, M. (2015). Real-time assessment of heart rate variability and loss of control eating in adolescent girls: A pilot study. The International journal of eating disorders, 49(2), 197-201. 10.1002/eat.22464

Ranzenhofer, L. M., Solhjoo, S., Crosby, R. D., Kim, B. H., Korn, R., Koorathota, S., Lloyd, E. C., Walsh, B. T., & Haigney, M. C. (2023). Autonomic indices and loss-of-control eating in adolescents: an ecological momentary assessment study. Psychological Medicine, 53(10), 4742-4750. https://dx.doi.org/10.1017/S0033291722001684

Rauschenberg, C., Schulte-Strathaus, J. C. C., van Os, J., Goedhart, M., Schieveld, J. N. M., & Reininghaus, U. (2022). Negative life events and stress sensitivity in youth's daily life: an ecological momentary assessment study. Social Psychiatry and Psychiatric Epidemiology, 25, 25. https://dx.doi.org/10.1007/s00127-022-02276-0

* Rauschenberg, C., van Os, J., Cremers, D., Goedhart, M., Schieveld, J. N. M., & Reininghaus, U. (2017). Stress sensitivity as a putative mechanism linking childhood trauma and psychopathology in youth's daily life. Acta Psychiatrica Scandinavica, 136(4), 373-388. https://dx.doi.org/10.1111/acps.12775

Rauschenberg, C., van Os, J., Goedhart, M., Schieveld, J. N. M., & Reininghaus, U. (2021). Bullying victimization and stress sensitivity in help-seeking youth: findings from an

experience sampling study. European Child and Adolescent Psychiatry, 30(4), 591-605. https://dx.doi.org/10.1007/s00787-020-01540-5

Reid, S., Kauer, S., Hearps, S., Crooke, A. H. D., Khor, A. S., Sanci, L., & Patton, G. C. (2011). A mobile phone application for the assessment and management of youth mental health problems in primary care: a randomised controlled trial. BMC Family Practice, 12(1), 131-131. 10.1186/1471-2296-12-131

Reid, S., Kauer, S., Hearps, S., Crooke, A. H. D., Khor, A. S., Sanci, L., & Patton, G. C. (2013). A mobile phone application for the assessment and management of youth mental health problems in primary care: health service outcomes from a randomised controlled trial of mobiletype. BMC Family Practice, 14(1), 84-84. 10.1186/1471-2296-14-84

* Reid, S. C., Kauer, S. D., Dudgeon, P., Sanci, L. A., Shrier, L. A., & Patton, G. C. (2009). A mobile phone program to track young people's experiences of mood, stress and coping. Development and testing of the mobiletype program. Social Psychiatry and Psychiatric Epidemiology, 44(6), 501-507. https://dx.doi.org/10.1007/s00127-008-0455-5

Reitsema, A. M., Jeronimus, B. F., van Dijk, M., Ceulemans, E., van Roekel, E., Kuppens, P., & de Jonge, P. (2022). Distinguishing Dimensions of Emotion Dynamics Across 12 Emotions in Adolescents' Daily Lives. Emotion. 10.1037/emo0001173

Ren, B., Balkind, E. G., Pastro, B., Israel, E. S., Pizzagalli, D. A., Rahimi-Eichi, H., Baker, J. T., & Webb, C. A. (2023). Predicting states of elevated negative affect in adolescents from smartphone sensors: a novel personalized machine learning approach. Psychological Medicine, 53(11), 5146-5154. https://dx.doi.org/10.1017/S0033291722002161

* Rende, R., Slomkowski, C., Floro, J., & Jamner, L. (2009). Capturing rule breaking behavior between siblings in real time and everyday settings. European Journal of Developmental Science, 3(2), 150-160.

* Rhee, H., Belyea, M. J., Sterling, M., & Bocko, M. F. (2015). Evaluating the Validity of an Automated Device for Asthma Monitoring for Adolescents: Correlational Design. Journal of Medical Internet Research, 17(10), e234. https://dx.doi.org/10.2196/jmir.4975

Rich, M., Bickham, D. S., & Shrier, L. A. (2015). Measuring youth media exposure: A multimodal method for investigating the influence of media on digital natives. American Behavioral Scientist, 59(14), 1736-1754. https://dx.doi.org/10.1177/0002764215596558

Richmond, M. J., Mermelstein, R. J., & Wakschlag, L. S. (2013). Direct observations of parenting and real-time negative affect among adolescent smokers and nonsmokers. Journal of Clinical Child and Adolescent Psychology, 42(5), 617-628. https://dx.doi.org/10.1080/15374416.2012.738452

* Riddell, M. C., Gal, R. L., Bergford, S., Patton, S. R., Clements, M. A., Calhoun, P., Beaulieu, L. C., & Sherr, J. L. (2023). The Acute Effects of Real-World Physical Activity on Glycemia in Adolescents With Type 1 Diabetes: The Type 1 Diabetes Exercise Initiative Pediatric (T1DEXIP) Study. Diabetes Care, 03, 03. https://dx.doi.org/10.2337/dc23-1548

Rivenbark, J. G., Copeland, W. E., Davisson, E. K., Gassman-Pines, A., Hoyle, R. H., Piontak, J. R., Russell, M. A., Skinner, A. T., & Odgers, C. L. (2019). Perceived social status and mental health among young adolescents: Evidence from census data to cellphones. Developmental Psychology, 55(3), 574-585. https://dx.doi.org/10.1037/dev0000551

Roberts, M. E., Bidwell, L. C., Colby, S. M., & Gwaltney, C. J. (2015). With others or alone? Adolescent individual differences in the context of smoking lapses. Health Psychology, 34(11), 1066-1075. https://dx.doi.org/10.1037/hea0000211

Roberts, M. E., Keller-Hamilton, B., & Ferketich, A. K. (2022). Testing if attitudes mediate the association between advertising exposure and adolescent tobacco use. Addictive Behaviors, 134, 107415. https://dx.doi.org/10.1016/j.addbeh.2022.107415

* Roberts, M. E., Keller-Hamilton, B., Hinton, A., Browning, C. R., Slater, M. D., Xi, W., & Ferketich, A. K. (2019). The magnitude and impact of tobacco marketing exposure in adolescents' day-to-day lives: An ecological momentary assessment (EMA) study. Addictive Behaviors, 88, 144-149. https://dx.doi.org/10.1016/j.addbeh.2018.08.035

* Rodman, A. M., Vidal Bustamante, C. M., Dennison, M. J., Flournoy, J. C., Coppersmith, D. D., Nook, E. C., Worthington, S., Mair, P., & McLaughlin, K. A. (2021). A year in the social life of a teenager: Within-persons fluctuations in stress, phone communication, and anxiety and depression. Clinical Psychological Science, 9(5), 791-809. https://dx.doi.org/10.1177/2167702621991804

* Ronka, A., Sevon, E., Raikkonen, E., & Hintikka, T. (2017). Manuscript: You have a Message from Illi! The Mobile Diary in Researching Children's Daily Experiences. Child Indicators Research, 10(2), 505-523. 10.1007/s12187-016-9386-y

* Rosen, P. J., Epstein, J. N., & Van Orden, G. (2013). I know it when I quantify it: ecological momentary assessment and recurrence quantification analysis of emotion dysregulation in children with ADHD. Attention Deficit and Hyperactivity Disorders, 5(3), 283-294. https://dx.doi.org/10.1007/s12402-013-0101-2

* Rosen, P. J., & Factor, P. I. (2015). Emotional Impulsivity and Emotional and Behavioral Difficulties Among Children With ADHD: An Ecological Momentary Assessment Study. Journal of Attention Disorders, 19(9), 779-793. https://dx.doi.org/10.1177/1087054712463064

* Ross, K., Martin, T., Chen, E., & Miller, G. E. (2011). Social encounters in daily life and 2-year changes in metabolic risk factors in young women. Development and Psychopathology, 23(3), 897-906. https://dx.doi.org/10.1017/S0954579411000381

Rothenberg, W. A., Di Giunta, L., Lansford, J. E., Lunetti, C., Fiasconaro, I., Basili, E., Thartori, E., Favini, A., Pastorelli, C., Eisenberg, N., Amico, F., Rosa, M., & Cirimele, F. (2019). Daily Associations between Emotions and Aggressive and Depressive Symptoms in Adolescence: The Mediating and Moderating Role of Emotion Dysregulation. Journal of Youth & Adolescence, 48(11), 2207-2221. https://dx.doi.org/10.1007/s10964-019-01071-6

* Rumbold, J., Fletcher, D., & Daniels, K. (2020). An experience sampling study of organizational stress processes and future playing time in professional sport. Journal of Sports Sciences, 38(5), 559-567. https://dx.doi.org/10.1080/02640414.2020.1717302

* Rusby, J. C., Westling, E., Crowley, R., & Light, J. M. (2013). Concurrent and predictive associations between early adolescent perceptions of peer affiliates and mood states collected in real time via ecological momentary assessment methodology. Psychological Assessment, 25(1), 47-60. https://dx.doi.org/10.1037/a0030393

Rusby, J. C., Westling, E., Crowley, R., & Light, J. M. (2014). Psychosocial correlates of physical and sedentary activities of early adolescent youth. Health Education and Behavior, 41(1), 42-51. https://dx.doi.org/10.1177/1090198113485753

* Rusby, J. C., Westling, E., Crowley, R., Mills, K. L., & Light, J. M. (2019). Associations between marijuana use and anxious mood lability during adolescence. Addictive Behaviors, 92, 89-94. https://dx.doi.org/10.1016/j.addbeh.2018.12.029

Russell, M. A. (2015). A within-person approach to understanding daily experiences and well-being among adolescents in socioeconomic disadvantage: Vulnerability and opportunity in vivo. Dissertation Abstracts International: Section B: The Sciences and Engineering, 75(11-B(E)), No Pagination Specified.

Russell, M. A., & Odgers, C. L. (2020). Adolescents' Subjective Social Status Predicts Day-to-Day Mental Health and Future Substance Use. Journal of Research on Adolescence, 30 Suppl 2, 532-544. https://dx.doi.org/10.1111/jora.12496

* Russell, M. A., Wang, L., & Odgers, C. L. (2016). Witnessing substance use increases same-day antisocial behavior among at-risk adolescents: Gene-environment interaction in a 30-day ecological momentary assessment study. Development and Psychopathology, 28(4pt2), 1441-1456.

* Saarikallio, S. H., Randall, W. M., & Baltazar, M. (2019). Music Listening for Supporting Adolescents' Sense of Agency in Daily Life. Frontiers in Psychology, 10, 2911. https://dx.doi.org/10.3389/fpsyg.2019.02911

Salamon, R., Grondin, O., & Swendsen, J. (2009). Investigation of adolescent behavioral and emotional problems: Feasibility and validity of the ESM. Journal de Therapie Comportementale et Cognitive, 19(1), 41-46. https://dx.doi.org/10.1016/j.jtcc.2009.04.004

* Salamon, R., Husky, M. M., & Swendsen, J. D. (2013). Self-esteem and emotional well-being of students in academic difficulty: A study in daily life. Journal de Therapie Comportementale et Cognitive, 23(1), 24-30. https://dx.doi.org/10.1016/j.jtcc.2012.11.002

Salamon, R., Johnson, E. I., & Swendsen, J. (2011). Daily life mechanisms of stress spillover among early adolescents experiencing academic difficulty. European Journal of Psychology of Education, 26(4), 453-463. 10.1007/s10212-011-0056-7

Salamon, R., Swendsen, J. D., & Husky, M. M. (2014). Extracurricular behavior and activities: A daily life study of academic failure and success. Annales Medico-Psychologiques, 172(4), 268-272. 10.1016/j.amp.2013.07.003

* Salmela-Aro, K., Moeller, J., Schneider, B., Spicer, J., & Lavonen, J. (2016). Integrating the light and dark sides of student engagement using person-oriented and situation-specific approaches. Learning and Instruction, 43, 61-70. https://dx.doi.org/10.1016/j.learninstruc.2016.01.001

Salmela-Aro, K., Upadyaya, K., Cumsille, P., Lavonen, J., Avalos, B., & Eccles, J. (2021). Momentary task-values and expectations predict engagement in science among Finnish and Chilean secondary school students. International Journal of Psychology, 56(3), 415-424. https://dx.doi.org/10.1002/ijop.12719

* Salvy, S. J., Bowker, J. W., Roemmich, J. N., Romero, N., Kieffer, E., Paluch, R., & Epstein, L. H. (2008). Peer influence on children's physical activity: an experience sampling study. Journal of Pediatric Psychology, 33(1), 39-49.

Salvy, S. J., Feda, D. M., Epstein, L. H., & Roemmich, J. N. (2017). Friends and social contexts as unshared environments: a discordant sibling analysis of obesity- and health-related behaviors in young adolescents. International Journal of Obesity, 41(4), 569-575. https://dx.doi.org/10.1038/ijo.2016.213

Salvy, S. J., Feda, D. M., Epstein, L. H., & Roemmich, J. N. (2017). The social context moderates the relationship between neighborhood safety and adolescents' activities. Preventive Medicine Reports, 6, 355-360. https://dx.doi.org/10.1016/j.pmedr.2017.04.009

Santangelo, P. S., Koenig, J., Funke, V., Parzer, P., Resch, F., Ebner-Priemer, U. W., & Kaess, M. (2017). Ecological Momentary Assessment of Affective and Interpersonal Instability in Adolescent Non-Suicidal Self-Injury. Journal of Abnormal Child Psychology, 45(7), 1429-1438. https://dx.doi.org/10.1007/s10802-016-0249-2

* Schappo, C., Garanhani, R. R., Cordeiro, M. E. W., Oppitz, L. R., Schneider, N. A., Tanaka, O. M., Arantes, A. C. M., Ignacio, S. A., Alanis, L. R. A., Stuginski-Barbosa, J., & Camargo, E. S. (2023). Assessment of awake bruxism and oral mucosa indentation in adolescents. Journal of Oral Rehabilitation, 50(8), 671-678. https://dx.doi.org/10.1111/joor.13473

* Scharf, D. M., Martino, S. C., Setodji, C. M., Staplefoote, B. L., & Shadel, W. G. (2013). Middle and high school students' exposure to alcohol- and smoking-related media: a pilot

study using ecological momentary assessment. Psychology of Addictive Behaviors, 27(4), 1201-1206. https://dx.doi.org/10.1037/a0032555

Schmidt, A., Dirk, J., Neubauer, A. B., & Schmiedek, F. (2021). Evaluating sociometer theory in children's everyday lives: Inclusion, but not exclusion by peers at school is related to within-day change in self-esteem. European Journal of Personality, 35(5), 736-753. 10.1177/0890207020962328

* Schmidt, A., Dirk, J., & Schmiedek, F. (2019). The importance of peer relatedness at school for affective well-being in children: Between- and within-person associations. Social Development, 28(4), 873-892. https://dx.doi.org/10.1111/sode.12379

* Schmidt, A., Neubauer, A. B., Dirk, J., & Schmiedek, F. (2020). The bright and the dark side of peer relationships: Differential effects of relatedness satisfaction and frustration at school on affective well-being in children's daily lives. Developmental Psychology, 56(8), 1532-1546. https://dx.doi.org/10.1037/dev0000997

Schmidt, J. A., Beymer, P. N., Rosenberg, J. M., Naftzger, N. N., & Shumow, L. (2020). Experiences, activities, and personal characteristics as predictors of engagement in STEM-focused summer programs. Journal of Research in Science Teaching, 57(8), 1281-1309. https://dx.doi.org/10.1002/tea.21630

Schmidt, P., Jendryczko, D., Zurbriggen, C. L. A., & Nussbeck, F. W. (2023). Recall bias of students' affective experiences in adolescence: The role of personality and internalizing behavior. Journal of Adolescence, 95(5), 893-906. https://dx.doi.org/10.1002/jad.12162

* Schmutz, C., Burgler, A., Ashta, N., Soenksen, J., Bou Karim, Y., Shen, C., Smith, R. B., Jenkins, R. H., Mireku, M. O., Mutz, J., Maes, M. J. A., Hirst, R., Chang, I., Fleming, C., Mussa, A., Kesary, D., Addison, D., Maslanyj, M., Toledano, M. B., Roosli, M., & Eeftens, M. (2022). Personal radiofrequency electromagnetic field exposure of adolescents in the Greater London area in the SCAMP cohort and the association with restrictions on permitted use of mobile communication technologies at school and at home. Environmental Research, 212(Pt B), 113252. https://dx.doi.org/10.1016/j.envres.2022.113252

* Schneider, B., Chen, I. C., Bradford, L., & Bartz, K. (2022). Intervention initiatives to raise young people's interest and participation in STEM. Frontiers in Psychology, 13, 960327. https://dx.doi.org/10.3389/fpsyg.2022.960327

Schneider, B., Krajcik, J., Lavonen, J., Salmela-Aro, K., Broda, M., Spicer, J., Bruner, J., Moeller, J., Linnansaari, J., Juuti, K., & Viljaranta, J. (2016). Investigating optimal learning moments in U.S. and Finnish science classes. Journal of Research in Science Teaching, 53(3), 400-421. https://dx.doi.org/10.1002/tea.21306

* Schneider-Worthington, C. R., Smith, K. E., Roemmich, J. N., & Salvy, S. J. (2022). External food cue responsiveness and emotional eating in adolescents: A multimethod study. Appetite, 168, 105789. https://dx.doi.org/10.1016/j.appet.2021.105789

Schreuder, M. J., Hartman, C. A., George, S. V., Menne-Lothmann, C., Decoster, J., van Winkel, R., Delespaul, P., De Hert, M., Derom, C., Thiery, E., Rutten, B. P. F., Jacobs, N., van Os, J., Wigman, J. T. W., & Wichers, M. (2020). Early warning signals in psychopathology: what do they tell? BMC Medicine, 18(1), 269. https://dx.doi.org/10.1186/s12916-020-01742-3

* Schurman, J. V., & Friesen, C. A. (2015). Identifying potential pediatric chronic abdominal pain triggers using ecological momentary assessment. Clinical Practice in Pediatric Psychology, 3(2), 131-141. https://dx.doi.org/10.1037/cpp0000095

* Schwartz-Mette, R. A., Duell, N., Lawrence, H. R., & Balkind, E. G. (2022). COVID-19 Distress Impacts Adolescents' Depressive Symptoms, NSSI, and Suicide Risk in the Rural, Northeast US. Journal of Clinical Child and Adolescent Psychology, 1-14. https://dx.doi.org/10.1080/15374416.2022.2042697

* Segovia-Siapco, G., & Sabate, J. (2016). Using Personal Mobile Phones to Assess Dietary Intake in Free-Living Adolescents: Comparison of Face-to-Face Versus Telephone Training. JMIR MHealth and UHealth, 4(3), e91. https://dx.doi.org/10.2196/mhealth.5418

Seidel, M., King, J. A., Fürtjes, S., Labitzke, N., Wronski, M. L., Boehm, I., Hennig, J., Gramatke, K., Roessner, V., & Ehrlich, S. (2022). Increased Habit Frequency in the Daily Lives of Patients with Acute Anorexia Nervosa. Nutrients, 14(19). 10.3390/nu14193905

Seidel, M., King, J. A., Ritschel, F., Boehm, I., Geisler, D., Bernardoni, F., Holzapfel, L., Diestel, S., Diers, K., Strobel, A., Goschke, T., Walter, H., Roessner, V., & Ehrlich, S. (2018). The real-life costs of emotion regulation in anorexia nervosa: a combined ecological momentary assessment and fMRI study. Transl Psychiatry Psychiatry, 8(1), 28. https://dx.doi.org/10.1038/s41398-017-0004-7

Seidel, M., Petermann, J., Diestel, S., Ritschel, F., Boehm, I., King, J. A., Geisler, D., Bernardoni, F., Roessner, V., Goschke, T., & Ehrlich, S. (2016). A naturalistic examination of negative affect and disorder-related rumination in anorexia nervosa. European Child and Adolescent Psychiatry, 25(11), 1207-1216.

Seidl, E., Venz, J., Ollmann, T. M., Voss, C., Hoyer, J., Pieper, L., & Beesdo-Baum, K. (2021). How current and past anxiety disorders affect daily life in adolescents and young adults from the general population-An epidemiological study with ecological momentary assessment. Depression and Anxiety, 38(3), 272-285. https://dx.doi.org/10.1002/da.23133

Seidl, E., Venz, J., Ollmann, T. M., Voss, C., Hoyer, J., Pieper, L., & Beesdo-Baum, K. (2023). Dynamics of affect, cognition and behavior in a general population sample of adolescents and young adults with current and remitted anxiety disorders: An Ecological Momentary Assessment study. Journal of Anxiety Disorders, 93, 102646. https://dx.doi.org/10.1016/j.janxdis.2022.102646

Seiffert, N., Cavelti, M., Koenig, J., Santangelo, P., Lerch, S., Resch, F., Ebner-Priemer, U., & Kaess, M. (2022). Object constancy in adolescents with borderline personality disorder pathology. Personality Disorders: Theory, Research, & Treatment, 31, 31. https://dx.doi.org/10.1037/per0000558

Selby, E. A., Nock, M. K., & Kranzler, A. (2014). How does self-injury feel? Examining automatic positive reinforcement in adolescent self-injurers with experience sampling. Psychiatry Research, 215(2), 417-423. https://dx.doi.org/10.1016/j.psychres.2013.12.005

Selya, A. S., Updegrove, N., Rose, J. S., Dierker, L., Tan, X., Hedeker, D., Li, R., & Mermelstein, R. J. (2015). Nicotine-dependence-varying effects of smoking events on momentary mood changes among adolescents. Addictive Behaviors, 41, 65-71. https://dx.doi.org/10.1016/j.addbeh.2014.09.028

Sequeira, S. L., Silk, J. S., Edershile, E. A., Jones, N. P., Hanson, J. L., Forbes, E. E., & Ladouceur, C. D. (2021). From scanners to cell phones: neural and real-world responses to social evaluation in adolescent girls. Social Cognitive and Affective Neuroscience, 16(7), 657-669. https://dx.doi.org/10.1093/scan/nsab038

* Shamblen, S. R., Abadi, M. H., Thompson, K. T., Garcia-Ramirez, G., & Richard, B. O. (2022). Changes in the Patterns and Characteristics of Youth ENDS Use over Time. International Journal of Environmental Research & Public Health [Electronic Resource], 19(13), 01. https://dx.doi.org/10.3390/ijerph19138120

Shamblen, S. R., Abadi, M. H., Thompson, K. T., Lipperman-Kreda, S., Grube, J. W., & Richard, B. O. (2022). Daily variation in the patterns and characteristics of adolescent ENDS use. Psychology of Addictive Behaviors, 13, 13. https://dx.doi.org/10.1037/adb0000810

Shapira, A., Volkening, L. K., Borus, J. S., & Laffel, L. M. (2021). Ecological Momentary Assessment (EMA) of Positive and Negative Affect and Associations with Blood Glucose (BG) in Teens with Type 1 Diabetes (T1D). Journal of Diabetes Science and Technology, 19322968211035451. https://dx.doi.org/10.1177/19322968211035451

* Shelton, N., Douglass, S., Garcia, R. L., Yip, T., & Trail, T. E. (2014). Feeling (mis)understood and intergroup friendships in interracial interactions. Personality and Social Psychology Bulletin, 40(9), 1193-1204. https://dx.doi.org/10.1177/0146167214538459

Shen, L., Wiley, J. F., & Bei, B. (2021). Perceived daily sleep need and sleep debt in adolescents: associations with daily affect over school and vacation periods. Sleep, 44(12), 10. https://dx.doi.org/10.1093/sleep/zsab190

Shen, L., Wiley, J. F., & Bei, B. (2022). Sleep and affect in adolescents: Bidirectional daily associations over 28-day ecological momentary assessment. Journal of Sleep Research, 31(2), e13491. https://dx.doi.org/10.1111/jsr.13491

Shingleton, R. M., Eddy, K. T., Keshaviah, A., Franko, D. L., Swanson, S. A., Yu, J. S., Krishna, M., Nock, M. K., & Herzog, D. B. (2013). Binge/purge thoughts in nonsuicidal self-injurious adolescents: an ecological momentary analysis. International Journal of Eating Disorders, 46(7), 684-689. https://dx.doi.org/10.1002/eat.22142

* Shiyko, M. P., Perkins, S., & Caldwell, L. (2017). Feasibility and adherence paradigm to ecological momentary assessments in urban minority youth. Psychological Assessment, 29(7), 926-934. https://dx.doi.org/10.1037/pas0000386

Siebers, T., Beyens, I., Pouwels, J., & Valkenburg, P. M. (2021). Social media and distraction: An experience sampling study among adolescents. Media Psychology, No Pagination Specified. https://dx.doi.org/10.1080/15213269.2021.1959350

* Siebers, T., Beyens, I., Pouwels, J. L., & Valkenburg, P. M. (2022). Explaining variation in adolescents' social media-related distraction: The role of social connectivity and disconnectivity factors. Current Psychology, 1-14. https://dx.doi.org/10.1007/s12144-022-03844-y

Siebers, T., Beyens, I., & Valkenburg, P. M. (2023). The effects of fragmented and sticky smartphone use on distraction and task delay. Mobile Media & Communication, 26. 10.1177/20501579231193941

* Skimina, E., Karas, D., Topolewska-Siedzik, E., Klym-Guba, M., Ponikiewska, K., Rogoza, R., Davidov, E., & Cieciuch, J. (2020). A categorization of behaviors reported in experience sampling studies. Social Psychological Bulletin, 15(2), 1-33. https://dx.doi.org/10.32872/spb.3029

Slot, E., Akkerman, S., & Wubbels, T. (2019). Adolescents' interest experience in daily life in and across family and peer contexts. European Journal of Psychology of Education, 34(1), 25-43. https://dx.doi.org/10.1007/s10212-018-0372-2

Slot, E. M., Bronkhorst, H., Wubbels, T., & Akkerman, S. F. (2020). The role of school in adolescents' interest in daily life. International Journal of Educational Research, 104, 12, Article 101643. 10.1016/j.ijer.2020.101643

* Slot, E. M., Vulperhorst, J. P., Bronkhorst, L. H., van Der Rijst, R. M., Wubbels, T., & Akkerman, S. F. (2020). Mechanisms of interest sustainment. Learning Culture and Social Interaction, 24, 16, Article 100356. 10.1016/j.lcsi.2019.100356

* Smelror, R. E., Bless, J. J., Hugdahl, K., & Agartz, I. (2019). Feasibility and Acceptability of Using a Mobile Phone App for Characterizing Auditory Verbal Hallucinations in Adolescents With Early-Onset Psychosis: Exploratory Study. JMIR Formative Research, 3(2), e13882. https://dx.doi.org/10.2196/13882

* Smith, A. R., Jones, E. L., Subar, A. R., Do, Q. B., Kircanski, K., Leibenluft, E., Brotman, M. A., Pine, D. S., & Silk, J. S. (2022). The role of anxiety and gender in anticipation and avoidance of naturalistic anxiety-provoking experiences during adolescence: An ecological momentary assessment study. Jcpp Advances, 2(3), e12084. https://dx.doi.org/10.1002/jcv2.12084

Smith, A. R., Kircanski, K., Brotman, M. A., Do, Q. B., Subar, A. R., Silk, J. S., Engel, S., Crosby, R. D., Harrewijn, A., White, L. K., Haller, S. P., Cardinale, E. M., Buzzell, G. A., Barker, T., Leibenluft, E., & Pine, D. S. (2019). Advancing clinical neuroscience through enhanced tools: Pediatric social anxiety as an example. Depression and Anxiety, 36(8), 701-711. https://dx.doi.org/10.1002/da.22937

Smith, K. E., Haedt-Matt, A., Dougherty, E. N., Ivins-Lukse, M., & Goldschmidt, A. B. (2020). The interactive effects of parental self-efficacy and child eating styles in relation to naturalistically-assessed craving, overeating, and loss of control eating. International Journal of Eating Disorders, 53(9), 1450-1459. https://dx.doi.org/10.1002/eat.23296

Smith, K. E., Haedt-Matt, A., Mason, T. B., Wang, S., Yang, C. H., Unick, J. L., Bond, D., & Goldschmidt, A. B. (2020). Associations between naturalistically assessed physical activity patterns, affect, and eating in youth with overweight and obesity. Journal of Behavioral Medicine, 43(6), 916-931. https://dx.doi.org/10.1007/s10865-020-00152-3

Smith, K. E., Mason, T. B., O'Connor, S. M., Wang, S., Dzubur, E., Crosby, R. D., Wonderlich, S. A., Salvy, S. J., Feda, D. M., & Roemmich, J. N. (2021). Bi-Directional Associations Between Real-Time Affect and Physical Activity in Weight-Discordant Siblings. Journal of Pediatric Psychology, 46(4), 443-453. https://dx.doi.org/10.1093/jpepsy/jsaa121

Smith, K. E., O'Connor, S. M., Mason, T. B., Wang, S., Dzubur, E., Crosby, R. D., Wonderlich, S. A., Salvy, S. J., Feda, D. M., & Roemmich, J. N. (2021). Associations between objective physical activity and emotional eating among adiposity-discordant siblings using ecological momentary assessment and accelerometers. Pediatric Obesity, 16(3), e12720. https://dx.doi.org/10.1111/ijpo.12720

Smith, M. R., Seldin, K., Galtieri, L. R., Alawadhi, Y. T., Lengua, L. J., & King, K. M. (2023). Specific emotion and momentary emotion regulation in adolescence and early adulthood. Emotion, 23(4), 1011-1027. https://dx.doi.org/10.1037/emo0001127

* Sokolovsky, A. W., Mermelstein, R. J., & Hedeker, D. (2014). Factors predicting compliance to ecological momentary assessment among adolescent smokers. Nicotine & Tobacco Research, 16(3), 351-358. https://dx.doi.org/10.1093/ntr/ntt154

Spicer, J., Giesbrecht, G. F., Aboelela, S., Lee, S., Liu, G., & Monk, C. (2019). Ambulatory Blood Pressure Trajectory and Perceived Stress in Relation to Birth Outcomes in Healthy Pregnant Adolescents. Psychosomatic Medicine, 81(5), 464-476. https://dx.doi.org/10.1097/PSY.0000000000000698

Spicer, J., Werner, E., Zhao, Y., Choi, C. W., Lopez-Pintado, S., Feng, T., Altemus, M., Gyamfi, C., & Monk, C. (2013). Ambulatory assessments of psychological and peripheral stress-markers predict birth outcomes in teen pregnancy. Journal of Psychosomatic Research, 75(4), 305-313. https://dx.doi.org/10.1016/j.jpsychores.2013.07.001

* Starr, L. R., Hershenberg, R., Shaw, Z. A., Li, Y. I., & Santee, A. C. (2020). The Perils of Murky Emotions: Emotion Differentiation Moderates the Prospective Relationship Between Naturalistic Stress Exposure and Adolescent Depression. Emotion, 20(6), 927-938. 10.1037/emo0000630

Starr, L. R., Santee, A. C., Chang, K. K., & DeLap, G. A. L. (2023). Everyday emotion, naturalistic life stress, and the prospective prediction of adolescent depression. Anxiety, Stress, & Coping, 1-14. https://dx.doi.org/10.1080/10615806.2023.2267466

Starr, L. R., Shaw, Z. A., Li, Y. I., Santee, A. C., & Hershenberg, R. (2020). Negative Emotion Differentiation through a Developmental Lens: Associations with Parental Factors and Age in Adolescence. Personality and Individual Differences, 152, 01. https://dx.doi.org/10.1016/j.paid.2019.109597

Stinson, J. N., Jibb, L. A., Lalloo, C., Feldman, B. M., McGrath, P. J., Petroz, G. C., Streiner, D., Dupuis, A., Gill, N., & Stevens, B. J. (2014). Comparison of average weekly pain using recalled paper and momentary assessment electronic diary reports in children with arthritis. Clinical Journal of Pain, 30(12), 1044-1050. https://dx.doi.org/10.1097/AJP.0000000000000072

* Stinson, J. N., Jibb, L. A., Nguyen, C., Nathan, P. C., Maloney, A. M., Dupuis, L. L., Gerstle, J. T., Alman, B., Hopyan, S., Strahlendorf, C., Portwine, C., Johnston, D. L., & Orr, M. (2013). Development and testing of a multidimensional iPhone pain assessment application for adolescents with cancer. Journal of Medical Internet Research, 15(3), e51. https://dx.doi.org/10.2196/jmir.2350

* Stinson, J. N., Jibb, L. A., Nguyen, C., Nathan, P. C., Maloney, A. M., Dupuis, L. L., Gerstle, J. T., Hopyan, S., Alman, B. A., Strahlendorf, C., Portwine, C., & Johnston, D. L. (2015). Construct validity and reliability of a real-time multidimensional smartphone app to assess pain in children and adolescents with cancer. Pain, 156(12), 2607-2615. https://dx.doi.org/10.1097/j.pain.0000000000000385

Stinson, J. N., Petroz, G. C., Stevens, B. J., Feldman, B. M., Streiner, D., McGrath, P. J., & Gill, N. (2008). Working out the kinks: testing the feasibility of an electronic pain diary for adolescents with arthritis. Pain Research & Management, 13(5), 375-382.

* Stinson, J. N., Stevens, B. J., Feldman, B. M., Streiner, D., McGrath, P. J., Dupuis, A., Gill, N., & Petroz, G. C. (2008). Construct validity of a multidimensional electronic pain diary for adolescents with arthritis. Pain, 136(3), 281-292. https://dx.doi.org/10.1016/j.pain.2007.07.002

Stinson, J. N., Stevens, B. J., Feldman, B. M., Streiner, D. L., McGrath, P. J., Dupuis, A., Gill, N., & Petroz, G. C. (2011). Using an electronic pain diary to better understand pain in children and adolescents with arthritis. Pain Management, 1(2), 127-137. https://dx.doi.org/10.2217/pmt.11.2

* Streb, J., Kammer, T., Spitzer, M., & Hille, K. (2015). Extremely reduced motion in front of screens: investigating real-world physical activity of adolescents by accelerometry and electronic diary. PLoS ONE [Electronic Resource], 10(5), e0126722. https://dx.doi.org/10.1371/journal.pone.0126722

* Streb, J., Keis, O., Lau, M., Hille, K., Spitzer, M., & Sosic-Vasic, Z. (2015). Emotional engagement in kindergarten and school children: A self-determination theory perspective. Trends in Neuroscience and Education, 4(4), 102-107. 10.1016/j.tine.2015.11.001

* Sufrinko, A. M., Howie, E. K., Charek, D. B., Elbin, R. J., Collins, M. W., & Kontos, A. P. (2019). Mobile Ecological Momentary Assessment of Postconcussion Symptoms and Recovery Outcomes. Journal of Head Trauma Rehabilitation, 34(6), E40-E48. https://dx.doi.org/10.1097/HTR.0000000000000474

Suter, M., Pihet, S., Zimmermann, G., de Ridder, J., Urben, S., & Stephan, P. (2017). Predicting daily-life antisocial behaviour in institutionalized adolescents with Transgression-related Implicit Association Tests. Journal of Forensic Psychiatry and Psychology, 28(6), 881-900. 10.1080/14789949.2017.1332772

Suveg, C., Payne, M., Thomassin, K., & Jacob, M. L. (2010). Electronic diaries: A feasible method of assessing emotional experiences in youth? Journal of Psychopathology and Behavioral Assessment, 32(1), 57-67. 10.1007/s10862-009-9162-0

* Sweenie, R., Cushing, C. C., Fleming, K. K., Prabhakaran, S., & Fedele, D. A. (2022). Daily adherence variability and psychosocial differences in adolescents with asthma: a pilot study. Journal of Behavioral Medicine, 45(1), 148-158. https://dx.doi.org/10.1007/s10865-021-00247-5

* Swendeman, D., Sumstine, S., Brink, A., Mindry, D., Medich, M., & Russell, M. (2020). Smartphone Self-Monitoring by Young Adolescents and Parents to Assess and Improve Family Functioning: Qualitative Feasibility Study. JMIR Formative Research, 4(6), e15777. https://dx.doi.org/10.2196/15777

Swords, C. M., Lecarie, E. K., Doane, L. D., & Hilt, L. M. (2021). Psychological well-being of ruminative adolescents during the transition to COVID-19 school closures: An EMA study. Journal of Adolescence, 92, 189-193. https://dx.doi.org/10.1016/j.adolescence.2021.09.006

* Talic, I., Scherer, R., Marsh, H. W., Greiff, S., Moller, J., & Niepel, C. (2022). Uncovering everyday dynamics in students' perceptions of instructional quality with experience sampling. Learning and Instruction, 81, 1-14. https://dx.doi.org/10.1016/j.learninstruc.2022.101594

* Tasian, G. E., Ross, M., Song, L., Audrain-McGovern, J., Wiebe, D., Warner, S. G., Henderson, B., Patel, A., & Furth, S. L. (2019). Ecological Momentary Assessment of Factors Associated with Water Intake among Adolescents with Kidney Stone Disease. Journal of Urology, 201(3), 606-614. https://dx.doi.org/10.1016/j.juro.2018.07.064

* Thomassin, K., Morelen, D., & Suveg, C. (2012). Emotion reporting using electronic diaries reduces anxiety symptoms in girls with emotion dysregulation. Journal of Contemporary Psychotherapy, 42(4), 207-213. 10.1007/s10879-012-9205-9

* Tkaczyk, M., Lacko, D., Elavsky, S., Tancoš, M., & Smahel, D. (2023). Are smartphones detrimental to adolescent sleep? An electronic diary study of evening smartphone use and sleep. Computers in Human Behavior, 149, N.PAG-N.PAG. 10.1016/j.chb.2023.107946

Trbovich, A. M., Howie, E. K., Elbin, R. J., Ernst, N., Stephenson, K., Collins, M. W., & Kontos, A. P. (2021). The relationship between accelerometer-measured sleep and next day ecological momentary assessment symptom report during sport-related concussion recovery. Sleep Health, 7(4), 519-525. https://dx.doi.org/10.1016/j.sleh.2021.03.006

* Treloar Padovano, H., Janssen, T., Emery, N. N., Carpenter, R. W., & Miranda, R., Jr. (2019). Risk-Taking Propensity, Affect, and Alcohol Craving in Adolescents' Daily Lives. Substance Use and Misuse, 54(13), 2218-2228. https://dx.doi.org/10.1080/10826084.2019.1639753

Treloar Padovano, H., Merrill, J. E., Colby, S. M., Kahler, C. W., & Gwaltney, C. J. (2020). Affective and Situational Precipitants of Smoking Lapses Among Adolescents. Nicotine & Tobacco Research, 22(4), 492-497. https://dx.doi.org/10.1093/ntr/ntz002

Treloar Padovano, H., & Miranda, R., Jr. (2021). Incubation of alcohol craving as it naturally occurs in a developmentally diverse sample of dependent and nondependent drinkers. Addiction Biology, 26(3), e12934. https://dx.doi.org/10.1111/adb.12934

Tseng, W. L., Naim, R., Chue, A., Shaughnessy, S., Meigs, J., Pine, D. S., Leibenluft, E., Kircanski, K., & Brotman, M. A. (2023). Network analysis of ecological momentary assessment identifies frustration as a central node in irritability. Journal of Child Psychology and Psychiatry and Allied Disciplines, 64(8), 1212-1221. https://dx.doi.org/10.1111/jcpp.13794

Tupper, S. M., Rosenberg, A. M., Pahwa, P., & Stinson, J. N. (2013). Pain intensity variability and its relationship with quality of life in youths with juvenile idiopathic arthritis. Arthritis Care & Research, 65(4), 563-570. https://dx.doi.org/10.1002/acr.21850

* Turner, A. L., Brokamp, C., Wolfe, C., Reponen, T., & Ryan, P. H. (2022). Impact of Personal, Sub-hourly Exposure to Ultrafine Particles on Respiratory Health in Adolescents With Asthma. Annals of the American Thoracic Society, 22, 22. https://dx.doi.org/10.1513/AnnalsATS.202108-947OC

* Turri, F., Jones, A., Constanty, L., Ranjbar, S., Drexl, K., Miano, G., Lepage, C., Plessen, K. J., & Urben, S. (2023). Self-regulatory control processes in youths: A temporal network analysis approach. JCPP Advances, No Pagination Specified. https://dx.doi.org/10.1002/jcv2.12200

* Tutelman, P. R., Chambers, C. T., Stinson, J. N., Parker, J. A., Barwick, M., Witteman, H. O., Jibb, L., Stinson, H. C., Fernandez, C. V., Nathan, P. C., Campbell, F., & Irwin, K. (2018). The Implementation Effectiveness of a Freely Available Pediatric Cancer Pain Assessment App: A Pilot Implementation Study. JMIR Cancer, 4(2), e10280. https://dx.doi.org/10.2196/10280

Uink, B., Modecki, K. L., Barber, B. L., & Correia, H. M. (2018). Socioeconomically Disadvantaged Adolescents with Elevated Externalizing Symptoms Show Heightened Emotion Reactivity to Daily Stress: An Experience Sampling Study. Child Psychiatry and Human Development, 49(5), 741-756. https://dx.doi.org/10.1007/s10578-018-0784-x

Uink, B. N., Modecki, K. L., & Barber, B. L. (2017). Disadvantaged youth report less negative emotion to minor stressors when with peers: An experience sampling study. International Journal of Behavioral Development, 41(1), 41-51. 10.1177/0165025415626516

Upadyaya, K., Cumsille, P., Avalos, B., Araneda, S., Lavonen, J., & Salmela-Aro, K. (2021). Patterns of situational engagement and task values in science lessons. The Journal of Educational Research, 114(4), 394-403. https://dx.doi.org/10.1080/00220671.2021.1955651

Uy, J. P., & Galván, A. (2017). Acute stress increases risky decisions and dampens prefrontal activation among adolescent boys. Neuroimage, 146, 679-689. 10.1016/j.neuroimage.2016.08.067

Vaessen, T., van Nierop, M., Decoster, J., Delespaul, P., Derom, C., de Hert, M., Jacobs, N., Menne-Lothmann, C., Rutten, B., Thiery, E., van Os, J., van Winkel, R., Wichers, M., & Myin-Germeys, I. (2017). Is sensitivity to daily stress predictive of onset or persistence of psychopathology? European Psychiatry: the Journal of the Association of European Psychiatrists, 45, 167-173. https://dx.doi.org/10.1016/j.eurpsy.2017.07.002

Valkenburg, P., Beyens, I., Pouwels, J., van Driel, I. I., & Keijsers, L. (2021). Social media use and adolescents' self-esteem: Heading for a person-specific media effects paradigm. Journal of Communication, 71(1), 56-78. https://dx.doi.org/10.1093/joc/jqaa039

Valkenburg, P. M., Beyens, I., Pouwels, J. L., van Driel, II, & Keijsers, L. (2021). Social Media Browsing and Adolescent Well-Being: Challenging the 'Passive Social Media Use Hypothesis'. Journal of Computer-Mediated Communication, 27(1), 19.

Valkenburg, P. M., Pouwels, J., Beyens, I., van Driel, I. I., & Keijsers, L. (2021). Adolescents' social media experiences and their self-esteem: A person-specific susceptibility perspective. Technology, Mind, and Behavior, 2(2), No Pagination Specified. https://dx.doi.org/10.1037/tmb0000037

Valrie, C. R., Alston, K., Morgan, K., Kilpatrick, R., Sisler, I., & Fuh, B. (2021). Pediatric sickle cell pain-sleep relationships: The roles of positive and negative affect. Health Psychology, 40(11), 793-802. https://dx.doi.org/10.1037/hea0001144

* Valrie, C. R., Kilpatrick, R. L., Alston, K., Trout, K., Redding-Lallinger, R., Sisler, I., & Fuh, B. (2019). Investigating the Sleep-Pain Relationship in Youth with Sickle Cell Utilizing mHealth Technology. Journal of Pediatric Psychology, 44(3), 323-332. https://dx.doi.org/10.1093/jpepsy/jsy105

* van der Kaap-Deeder, J., Bulow, A., Waterschoot, J., Truyen, I., & Keijsers, L. (2023). A moment of autonomy support brightens adolescents' mood: Autonomy support, psychological control and adolescent affect in everyday life. Child Development, 08, 08. https://dx.doi.org/10.1111/cdev.13942

* Van Liefferinge, D., Sonuga-Barke, E., Danckaerts, M., Fayn, K., Van Broeck, N., & van der Oord, S. (2018). Measuring child and adolescent emotional lability: How do questionnaire-based ratings relate to experienced and observed emotion in everyday life and experimental settings? International Journal of Methods in Psychiatric Research, 27(3), e1720. https://dx.doi.org/10.1002/mpr.1720

* Van Oort, C., Tupper, S. M., Rosenberg, A. M., Farthing, J. P., & Baxter-Jones, A. D. (2013). Safety and feasibility of a home-based six week resistance training program in juvenile idiopathic arthritis. Pediatric Rheumatology Online Journal, 11(1), 46. https://dx.doi.org/10.1186/1546-0096-11-46

van Roekel, E., Bennik, E. C., Bastiaansen, J. A., Verhagen, M., Ormel, J., Engels, R. C., & Oldehinkel, A. J. (2016). Depressive Symptoms and the Experience of Pleasure in Daily Life: An Exploration of Associations in Early and Late Adolescence. Journal of Abnormal Child Psychology, 44(5), 999-1009. https://dx.doi.org/10.1007/s10802-015-0090-z

* van Roekel, E., Goossens, L., Verhagen, M., Wouters, S., Engels, R. C., & Scholte, R. H. (2014). Loneliness, affect, and adolescents' appraisals of company: An experience sampling method study. Journal of Research on Adolescence, 24(2), 350-363. https://dx.doi.org/10.1111/jora.12061

van Roekel, E., Ha, T., Verhagen, M., Kuntsche, E., Scholte, R. H., & Engels, R. C. (2015). Social stress in early adolescents' daily lives: Associations with affect and loneliness. Journal of Adolescence, 45, 274-283. https://dx.doi.org/10.1016/j.adolescence.2015.10.012

van Roekel, E., Scholte, R. H., Engels, R. C., Goossens, L., & Verhagen, M. (2015). Loneliness in the daily lives of adolescents: An experience sampling study examining the effects of social contexts. The Journal of Early Adolescence, 35(7), 905-930. https://dx.doi.org/10.1177/0272431614547049

van Roekel, E., Scholte, R. H. J., Engels, R. C. M. E., Goossens, L., & Verhagen, M. (2015). Loneliness in the Daily Lives of Adolescents. Journal of Early Adolescence, 35(7), 905-930. 10.1177/0272431614547049

van Roekel, E., Verhagen, M., Engels, R., Scholte, R. H. J., Cacioppo, S., & Cacioppo, J. T. (2018). Trait and State Levels of Loneliness in Early and Late Adolescents: Examining the Differential Reactivity Hypothesis. Journal of Clinical Child and Adolescent Psychology, 47(6), 888-899. https://dx.doi.org/10.1080/15374416.2016.1146993

van Roekel, E., Verhagen, M., Engels, R. C. M. E., & Kuppens, P. (2018). Variation in the serotonin transporter polymorphism (5-HTTLPR) and inertia of negative and positive emotions in daily life. Emotion, 18(2), 229-236. 10.1037/emo0000336

van Roekel, E., Verhagen, M., Scholte, R. H., Kleinjan, M., Goossens, L., & Engels, R. C. (2013). The oxytocin receptor gene (OXTR) in relation to state levels of loneliness in adolescence: evidence for micro-level gene-environment interactions. PLoS ONE [Electronic Resource], 8(11), e77689. https://dx.doi.org/10.1371/journal.pone.0077689

* van Woudenberg, T. J., Bevelander, K. E., Burk, W. J., & Buijzen, M. (2020). The reciprocal effects of physical activity and happiness in adolescents. International Journal of Behavioral Nutrition & Physical Activity, 17(1), 147. https://dx.doi.org/10.1186/s12966-020-01058-8

Vanwoerden, S., Byrd, A. L., Vine, V., Beeney, J. E., Scott, L. N., & Stepp, S. D. (2022). Momentary borderline personality disorder symptoms in youth as a function of parental invalidation and youth-perceived support. Journal of Child Psychology and Psychiatry and Allied Disciplines, 63(2), 178-186. https://dx.doi.org/10.1111/jcpp.13443

Verbeij, T., Pouwels, J. L., Beyens, I., & Valkenburg, P. M. (2021). The accuracy and validity of self-reported social media use measures among adolescents. Computers in Human Behavior Reports, 3, 11. 10.1016/j.chbr.2021.100090

Verbeij, T., Pouwels, J. L., Beyens, I., & Valkenburg, P. M. (2022). Experience sampling self-reports of social media use have comparable predictive validity to digital trace measures. Scientific Reports, 12(1), 7611. https://dx.doi.org/10.1038/s41598-022-11510-3

* Vilaysack, B., Cordier, R., Doma, K., & Chen, Y. W. (2016). Capturing everyday experiences of typically developing children aged five to seven years: A feasibility study of experience sampling methodology. Australian Occupational Therapy Journal, 63(6), 424-433. https://dx.doi.org/10.1111/1440-1630.12336

Vine, V., Victor, S. E., Mohr, H., Byrd, A. L., & Stepp, S. D. (2020). Adolescent suicide risk and experiences of dissociation in daily life. Psychiatry Research, 287, 112870. https://dx.doi.org/10.1016/j.psychres.2020.112870

Vize, C. E., Byrd, A. L., & Stepp, S. D. (2023). The Relative Importance of Psychopathy Features as Predictors of Externalizing Behaviors in Youth: A Multimethod Examination. Journal of Psychopathology & Behavioral Assessment, 45(1), 1-17. https://dx.doi.org/10.1007/s10862-022-10017-5

Vogelsmeier, L., Vermunt, J. K., Keijsers, L., & De Roover, K. (2021). Latent Markov Latent Trait Analysis for Exploring Measurement Model Changes in Intensive Longitudinal Data. Evaluation and the Health Professions, 44(1), 61-76. https://dx.doi.org/10.1177/0163278720976762

Vroegindeweij, A., Levelt, L., Houtveen, J., Van de Putte, E. M., Wulffraat, N. M., Swart, J. F., & Nijhof, S. L. (2023). Dynamic modeling of experience sampling methodology data reveals large heterogeneity in biopsychosocial factors associated with persistent fatigue in young people living with a chronic condition. Journal of Psychosomatic Research, 167, 111195. https://dx.doi.org/10.1016/j.jpsychores.2023.111195

Wagner, J., Wieczorek, L. L., & Brandt, N. D. (2023). Boosting yourself? Associations between momentary self-esteem, daily social interactions, and self-esteem development in late adolescence and late adulthood. Journal of Personality and Social Psychology, 04, 04. https://dx.doi.org/10.1037/pspp0000481

* Waldron, K. A., Lewis, M. A., Fairlie, A. M., Litt, D. M., Zhou, Z., & Bryant, D. (2023). Daily-level associations between alcohol use cognitions and normative perceptions among adolescents: An intensive longitudinal study. Addictive Behaviors, 143, 107697. https://dx.doi.org/10.1016/j.addbeh.2023.107697

Walsh, K., Basu, A., & Monk, C. (2015). The Role of Sexual Abuse and Dysfunctional Attitudes in Perceived Stress and Negative Mood in Pregnant Adolescents: An Ecological Momentary Assessment Study. Journal of Pediatric and Adolescent Gynecology, 28(5), 327-332. https://dx.doi.org/10.1016/j.jpag.2014.09.012

* Walsh, R. J., van Buuren, M., Hollarek, M., Sijtsma, H., Lee, N. C., & Krabbendam, L. (2023). Social Contexts, Momentary Mood and Affective Variability in Early Adolescence: An Exploratory Ecological Momentary Assessment Study. Journal of Early Adolescence, 37. 10.1177/02724316231160147

Wang, B., Nemesure, M. D., Park, C., Price, G. D., Heinz, M. V., & Jacobson, N. C. (2023). Leveraging deep learning models to understand the daily experience of anxiety in teenagers over the course of a year. Journal of Affective Disorders, 329, 293-299. 10.1016/j.jad.2023.02.084

* Wang, Y., Cham, H., Aladin, M., & Yip, T. (2019). Parental Cultural Socialization and Adolescent Private Regard: Exploring Mediating Pathways Through Daily Experiences. Child Development, 90(1), e19-e36. https://dx.doi.org/10.1111/cdev.12911

* Warnick, J. L., Westen, S. C., Albanese-O'Neill, A., Filipp, S. L., Schatz, D., Haller, M. J., & Janicke, D. M. (2020). Use of Ecological Momentary Assessment to Measure Self-

Monitoring of Blood Glucose Adherence in Youth With Type 1 Diabetes. Diabetes Spectrum, 33(3), 280-289. https://dx.doi.org/10.2337/ds19-0041

* Warren, C. M., & Pentz, M. A. (2019). The feasibility and acceptability of assessing inhibitory control and working memory among adolescents via an ecological momentary assessment approach. Child Neuropsychology, 25(8), 1022-1034. https://dx.doi.org/10.1080/09297049.2018.1556624

Webb, C. A., Israel, E. S., Belleau, E., Appleman, L., Forbes, E. E., & Pizzagalli, D. A. (2021). Mind-Wandering in Adolescents Predicts Worse Affect and Is Linked to Aberrant Default Mode Network-Salience Network Connectivity. Journal of the American Academy of Child and Adolescent Psychiatry, 60(3), 377-387. https://dx.doi.org/10.1016/j.jaac.2020.03.010

Webb, C. A., Murray, L., Tierney, A. O., Forbes, E. E., & Pizzagalli, D. A. (2023). Reward-related predictors of symptom change in behavioral activation therapy for anhedonic adolescents: a multimodal approach. Neuropsychopharmacology, 48(4), 623-632. https://dx.doi.org/10.1038/s41386-022-01481-4

Webb, C. A., Murray, L., Tierney, A. O., & Gates, K. M. (2023). Dynamic processes in behavioral activation therapy for anhedonic adolescents: Modeling common and patient-specific relations. Journal of Consulting and Clinical Psychology, 05, 05. https://dx.doi.org/10.1037/ccp0000830

* Webb, C. A., Swords, C. M., Lawrence, H. R., & Hilt, L. M. (2022). Which adolescents are well-suited to app-based mindfulness training? A randomized clinical trial and data-driven approach for personalized recommendations. Journal of Consulting and Clinical Psychology, 90(9), 655-669. https://dx.doi.org/10.1037/ccp0000763

* Webb, C. A., Swords, C. M., Murray, L., & Hilt, L. M. (2021). App-based Mindfulness Training for Adolescent Rumination: Predictors of Immediate and Cumulative Benefit. Mindfulness, 12(10), 2498-2509. https://dx.doi.org/10.1007/s12671-021-01719-0

Webb, C. A., Tierney, A. O., Brown, H. A., Forbes, E. E., Pizzagalli, D. A., & Ren, B. (2022). Spontaneous Thought Characteristics are Differentially Related to Heightened Negative Affect vs. Blunted Positive Affect in Adolescents: An Experience Sampling Study. Jcpp Advances, 2(4). https://dx.doi.org/10.1002/jcv2.12110

Weinstein, S. M., & Mermelstein, R. (2007). Relations between daily activities and adolescent mood: the role of autonomy. Journal of Clinical Child and Adolescent Psychology, 36(2), 182-194.

Weinstein, S. M., Mermelstein, R., Shiffman, S., & Flay, B. (2008). Mood variability and cigarette smoking escalation among adolescents. Psychology of Addictive Behaviors, 22(4), 504-513. https://dx.doi.org/10.1037/0893-164X.22.4.504

Weinstein, S. M., & Mermelstein, R. J. (2013). Dynamic associations of negative mood and smoking across the development of smoking in adolescence. Journal of Clinical Child

and Adolescent Psychology, 42(5), 629-642. https://dx.doi.org/10.1080/15374416.2013.794698

Weinstein, S. M., & Mermelstein, R. J. (2013). Influences of mood variability, negative moods, and depression on adolescent cigarette smoking. Psychology of Addictive Behaviors, 27(4), 1068-1078. https://dx.doi.org/10.1037/a0031488

Weinstein, S. M., Mermelstein, R. J., Hankin, B. L., Hedeker, D., & Flay, B. R. (2007). Longitudinal Patterns of Daily Affect and Global Mood During Adolescence. Journal of Research on Adolescence, 17(3), 587-600.

* Weinstein, S. M., Mermelstein, R. J., Hedeker, D., Hankin, B. L., & Flay, B. R. (2006). The time-varying influences of peer and family support on adolescent daily positive and negative affect. Journal of Clinical Child and Adolescent Psychology, 35(3), 420-430.

Weise, S., Parzer, P., Zimmermann, R., Furer, L., Resch, F., Kaess, M., & Koenig, J. (2020). Emotion dysregulation and resting-state autonomic function in adolescent borderline personality disorder-A multimodal assessment approach. Personality Disorders: Theory, Research, & Treatment, 11(1), 46-53. https://dx.doi.org/10.1037/per0000367

Wen, C. K. F., Liao, Y., Maher, J. P., Huh, J., Belcher, B. R., Dzubur, E., & Dunton, G. F. (2018). Relationships among affective states, physical activity, and sedentary behavior in children: Moderation by perceived stress. Health Psychology, 37(10), 904-914. https://dx.doi.org/10.1037/hea0000639

Whalen, C. K., Henker, B., Ishikawa, S. S., Jamner, L. D., Floro, J. N., Johnston, J. A., & Swindle, R. (2006). An electronic diary study of contextual triggers and ADHD: get ready, get set, get mad. Journal of the American Academy of Child and Adolescent Psychiatry, 45(2), 166-174. https://dx.doi.org/10.1097/01.chi.0000189057.67902.10

Whalen, C. K., Henker, B., King, P. S., Jamner, L. D., & Levine, L. (2004). Adolescents react to the events of September 11, 2001: focused versus ambient impact. Journal of Abnormal Child Psychology, 32(1), 1-11.

Whalen, C. K., Jamner, L. D., Henker, B., & Delfino, R. J. (2001). Smoking and moods in adolescents with depressive and aggressive dispositions: evidence from surveys and electronic diaries. Health Psychology, 20(2), 99-111.

Whalen, C. K., Jamner, L. D., Henker, B., Delfino, R. J., & Lozano, J. M. (2002). The ADHD spectrum and everyday life: experience sampling of adolescent moods, activities, smoking, and drinking. Child Development, 73(1), 209-227.

Whalen, C. K., Jamner, L. D., Henker, B., Gehricke, J. G., & King, P. S. (2003). Is there a link between adolescent cigarette smoking and pharmacotherapy for ADHD? Psychology of Addictive Behaviors, 17(4), 332-335.

* Whalen, C. K., Odgers, C. L., Reed, P. L., & Henker, B. (2011). Dissecting daily distress in mothers of children with ADHD: an electronic diary study. Journal of Family Psychology, 25(3), 402-411. https://dx.doi.org/10.1037/a0023473

* Wiebe, D. J., Storey, E. P., Orchinik, J. E., Grady, M. F., Leddy, J. J., Willer, B. S., Haider, M. N., Mannix, R., Meehan, W. P., Vernau, B. T., & Master, C. L. (2022). Measuring Recovery With Ecological Momentary Assessment in a Randomized Trial of Exercise After Sport-Related Concussion. Clinical Journal of Sport Medicine, 32(4), 345-353. 10.1097/JSM.0000000000000946

* Wieczorek, L. L., Bleckmann, E., Brandt, N. D., & Wagner, J. (2022). Gloomy and out of control? Consequences of the COVID-19 pandemic on momentary optimism in daily live of adolescents. Current Psychology, 1-11. https://dx.doi.org/10.1007/s12144-022-03313-6

* Wieczorek, L. L., Mueller, S., Ludtke, O., & Wagner, J. (2021). What makes for a pleasant social experience in adolescence? The role of perceived social interaction behavior in associations between personality traits and momentary social satisfaction. European Journal of Personality, 22, Article 08902070211017745. 10.1177/08902070211017745

* Williams, V., Romano, C., Clark, M., Korver, D., Williams, N., Goss, D., Naujoks, C., & Marvel, J. (2023). Psychometric evaluation of an electronic Asthma Symptom Diary for young children. Journal of Patientreported Outcomes, 7(1), 105. https://dx.doi.org/10.1186/s41687-023-00647-y

* Wilson, T. K., Riley, A., Khetarpal, S. K., Abernathy, P., Booth, J., & Culyba, A. J. (2023). Exploring the Impact of Racism on Black Youth: A Multidimensional Examination of Discriminatory Experiences Across Place and Time. Journal of Adolescent Health, 72(2), 246-253. https://dx.doi.org/10.1016/j.jadohealth.2022.09.028

* Wronski, M.-L., Hohnemann, C., Bernardoni, F., Bahnsen, K., Doose, A., Arold, D., Borucki, K., Holsen, L. M., Lawson, E. A., Plessow, F., Weidner, K., Roessner, V., Diestel, S., King, J. A., Seidel, M., & Ehrlich, S. (2023). Explicating the role of amygdala substructure alterations in the link between hypoleptinemia and rumination in anorexia nervosa. Acta Psychiatrica Scandinavica, 148(4), 368-381. https://dx.doi.org/10.1111/acps.13607

Wylie, M. S., De France, K., & Hollenstein, T. (2023). Adolescents suppress emotional expression more with peers compared to parents and less when they feel close to others. International Journal of Behavioral Development, 47(1), 1-8. https://dx.doi.org/10.1177/01650254221132777

* Xu, S., Wang, Z., & Woods, K. (2019). Multitasking and dual motivational systems: A dynamic longitudinal study. Human Communication Research, 45(4), 371-394. https://dx.doi.org/10.1093/hcr/hqz009

Yang, C. H., Huh, J., Mason, T. B., Belcher, B. R., Kanning, M., & Dunton, G. F. (2020). Mother-child dyadic influences of affect on everyday movement behaviors: evidence from an ecological momentary assessment study. International Journal of Behavioral Nutrition & Physical Activity, 17(1), 56. https://dx.doi.org/10.1186/s12966-020-00951-6

Yang, C. H., Zink, J., Belcher, B. R., Kanning, M., & Dunton, G. F. (2021). Age-varying Bi-directional Associations Between Momentary Affect and Movement Behaviors in Children: Evidence From a Multi-wave Ecological Momentary Assessment Study. Annals of Behavioral Medicine, 55(9), 918-931. https://dx.doi.org/10.1093/abm/kaaa124

Zenker, M., Venz, J., Koenig, J., Voss, C., Beesdo-Baum, K., & Pieper, L. (2021). Evidence for the association between physiological and emotional states in adolescents and young adults without psychopathology under ecologically valid conditions. Psychophysiology, 58(10), e13902. https://dx.doi.org/10.1111/psyp.13902

Zink, J., Belcher, B. R., Dzubur, E., Ke, W., O'Connor, S., Huh, J., Lopez, N., Maher, J. P., & Dunton, G. F. (2018). Association Between Self-Reported and Objective Activity Levels by Demographic Factors: Ecological Momentary Assessment Study in Children. JMIR MHealth and UHealth, 6(6), e150. https://dx.doi.org/10.2196/mhealth.9592

Zink, J., Nicolo, M., Imm, K., Ebrahimian, S., Yu, Q., Lee, K., Zapanta, K., Huh, J., Dunton, G. F., Goran, M. I., Page, K. A., Dieli-Conwright, C. M., & Belcher, B. R. (2020). Interstitial glucose and subsequent affective and physical feeling states: A pilot study combining continuous glucose monitoring and ecological momentary assessment in adolescents. Journal of Psychosomatic Research, 135, 110141. https://dx.doi.org/10.1016/j.jpsychores.2020.110141

Zink, J., Yang, C. H., Alves, J. M., McAlister, K. L., Huh, J., Pentz, M. A., Page, K. A., Dunton, G. F., & Belcher, B. R. (2022). Time-Varying Associations Between Device-Based and Ecological Momentary Assessment-Reported Sedentary Behaviors and the Concurrent Affective States Among Adolescents: Proof-of-Concept Study. JMIR Formative Research, 6(6), e37743. https://dx.doi.org/10.2196/37743

* Zurbriggen, C. L. A., Venetz, M., & Hinni, C. (2018). The quality of experience of students with and without special educational needs in everyday life and when relating to peers. European Journal of Special Needs Education, 33(2), 205-220. 10.1080/08856257.2018.1424777

Zuzama, N., Fiol-Veny, A., Roman-Juan, J., & Balle, M. (2020). Emotion Regulation Style and Daily Rumination: Potential Mediators between Affect and Both Depression and Anxiety during Adolescence. International Journal of Environmental Research & Public Health [Electronic Resource], 17(18), 11. https://dx.doi.org/10.3390/ijerph17186614

* Zuzama, N., Roman-Juan, J., Fiol-Veny, A., & Balle, M. (2021). The Use of Rumination and Reappraisal in Adolescents Daily Life: Links to Affect and Emotion Regulation Style. Child Psychiatry and Human Development, 17, 17. https://dx.doi.org/10.1007/s10578-021-01302-7
