# Supplementary material for: Readdressing the Ongoing Challenge of Missing Data in Youth Ecological Momentary Assessment Studies: Meta-Analysis Update
Source: J Med Internet Res. 2025 Apr 30;27:e65710. doi: 10.2196/65710 (PMC12079076; doi:10.2196/65710)

This is a Multimedia Appendix to the article **Readdressing the Ongoing Challenge of Missing Data in Youth Ecological Momentary Assessment Studies: Meta-Analysis Update** published in the Journal of Medical Internet Research. For full copyright and citation information see http://dx.doi.org/10.2196/jmir.65710

Drexl K[a], Ralisa V[a] Rosselet-Amoussou J[b], Wen CK[c1], Urben S[a], Plessen KJ[a], Glaus J[a]

[a]Division of Child and Adolescent Psychiatry, Department of Psychiatry, **Lausanne University Hospital and University of Lausanne**, Lausanne, Switzerland

[b]Medical Library-Cery, **Lausanne University Hospital and University of Lausanne**, Switzerland

[c]Dornsife Center for Self-Report Science, **University of Southern California**, Los Angeles, CA, USA

Detailed Results

Table of contents

Introductory Notes 3
Interrater Reliability 4
Table S1. Revision rates of extraction forms 4
Descriptive results 5
Table S2. Diagnostic categories 5
Figure S1. Transformation of continuous moderator variables. 7
Associations among included predictor variables 8
Figure S2. Correlation matrix of included continuous predictor variables 8
Figure S3. Correlation of study length and prompt frequency 9
Missing data patterns 10
Figure S4.1. Missingness clustered by reported acceptance 10
Figure S4.2. Missingness clustered by reported compliance 11
Figure S4.3. Missingness clustered by reported retention 12
Figure S5. Distributions participation metrics 13
Meta-analyses of Acceptance, Retention, and Compliance 14
Distribution of effect size estimates 14
Figure S6.1. Caterpillar plot of acceptance estimates 14
Figure S6.2. Caterpillar plot of compliance estimates 14
Figure S6.3. Caterpillar plot of retention estimates 15
Influential Samples 16
Figure S7.1. Studentized residuals and Cook's D scores for acceptance 18

Figure S7.2. Studentized residuals and Cook's D scores for compliance 18
Figure S7.3. Studentized residuals and Cook's D scores for retention 18
Senstivity Analyses 19
Table S3.1. Sensitivity to three-level meta-analysis and outlier removal 19
Table S3.2 Model comparisons across higher-order models 20
E9 Publication Bias 21
Figure S8.1. Funnel plot of acceptance and sample size 21
Figure S8.2. Funnel plot of compliance rates and sample size 22
Figure S8.3. Funnel plot of retention and sample size 23
Meta-regression results 24
Simple Regressions 24
Table S4.1. Sample characteristics predicting acceptance 24
Table S4.2. Design characteristics predicting acceptance 25
Table S4.3. Sample characteristics predicting compliance 26
Table S4.4. Design characteristics predicting compliance 27
Table S4.5. Sample characteristics predicting retention 28
Table S4.6. Design characteristics predicting retention 29
Figure S9.1. Meta-regression of acceptance on number of items 30
Figure S9.2. Meta-regression of compliance on publication year 30
Figure S9.3. Meta-regression of retention on study length 30
Sensitivity analyses 31
Figure S10. Meta-regressive effects from studies with uninflated compliance 31
Meta-regressions with interaction terms 32
Table S5.1. Interaction models predicting acceptance 32
Table S5.2. Interaction models predicting compliance 34
Table S5.3. Interaction models predicting retention 36
Meta-analyses of within study associations with compliance 38
Figure S11.1. Funnel plot for gender differences 38
Figure S11.2. Influence plot for gender differences 38
Figure S11.3. Funnel plot for age-compliance correlation 39
Figure S11.4. Influence plot for age-compliance correlation 39

Introductory Notes

The data and code that generated all statistical results, tables, figures, as well as this document itself are openly shared via the study's OSF repository (https://osf.io/8nkeu/).

Interrater Reliability

Blinded screening of titles and abstract yielded an interrater agreement of Cohen's $\kappa = 0.72$ (95%CI [0.7; 0.75]). Full-text screening resulted in higher agreement with Cohen's $\kappa = 0.81$ (95%CI [0.78; 0.84]). Revision rates of initial extractions quantified per variable and per section of the extraction form. The average rate of item-level modifications following review and discussion was 1.5% (SD 2.15, Range = 0.35 to 17.19). Revision on the level of entire sections are presented in **Table S1**. Note that the revision rates on the section level comprise *any* variable within the respective section that was revised, not the proportion of revised variables within the section.

Table S1. Revision rates of extraction forms

Extraction section	Revision rate (%)
Sample characteristics	11.58
General design	0.35
Recruitment, acceptance, retention	21.75
Ema schedule	11.58
Ema questionnaire	9.82
Technology	4.21
Assessment target	3.16
Compliance outcomes	19.30
Incentives, feedback, training, parent involvement	6.32

Descriptive Results

Table S2. Diagnostic categories

Harmonized diagnostic labels	Diagnosed	At-risk	Mixed	Total
**Somatic**				
Cancer	12	-	-	12
Asthma	8	-	1	9
Type 1 Diabetes	7	-	-	7
Concussion	5	-	-	5
Juvenile idiopathic arthrisis	5	-	-	5
Sickle cell desease	4	-	-	4
Migraine	2	-	-	2
Duchenne muscular dystrophy	1	-	-	1
Gastrointestinal	1	-	-	1
Hearing problems	1	-	-	1
Myopathie	1	-	-	1
Nephrolithiasis	1	-	-	1
Orthodontic	1	-	-	1
Overweight/obesity, binge-eating disorder	1	-	-	1
Progressive Familial Intrahepatic Cholestasis (PFIC)	1	-	-	1
Visual impairment	1	-	-	1
mixed/transdiagnostic sample	1	-	-	1
Total	53	0	1	54
**Psychiatric**				
ADHD	6	-	3	9
Cannabis, Nicotine, substance abuse	3	3	-	6
Depression	2	1	3	6
Suicidal thoughts and Behaviors	4	-	1	5

Harmonized diagnostic labels	Diagnosed	At-risk	Mixed	Total
Oppositional Defiant Disorder/ Conduct disorder	1	2	1	4
Anorexia nervosa	3	-	-	3
Childhood autism spectrum	3	-	-	3
Somatoform disorders	3	-	-	3
Nonsuicidal Self-Injury	2	-	1	3
Stress-related anxiety disorders	1	1	1	3
Anxiety disorders	2	-	-	2
Asperger syndrome	2	-	-	2
Alcohol abuse	1	-	1	2
Obesity, Binge eating	1	-	1	2
Schizophrenia spectrum disorder	1	-	1	2
mixed/transdiagnostic sample	1	-	-	1
Intellectual disability	-	-	1	1
Total	36	7	14	57
**Other**				
Mixed	8	1	-	9
Healthy or convenience sample	-	-	-	165
**Grand total**	**97**	**8**	**15**	**285**

Figure S1. Transformation of continuous moderator variables.

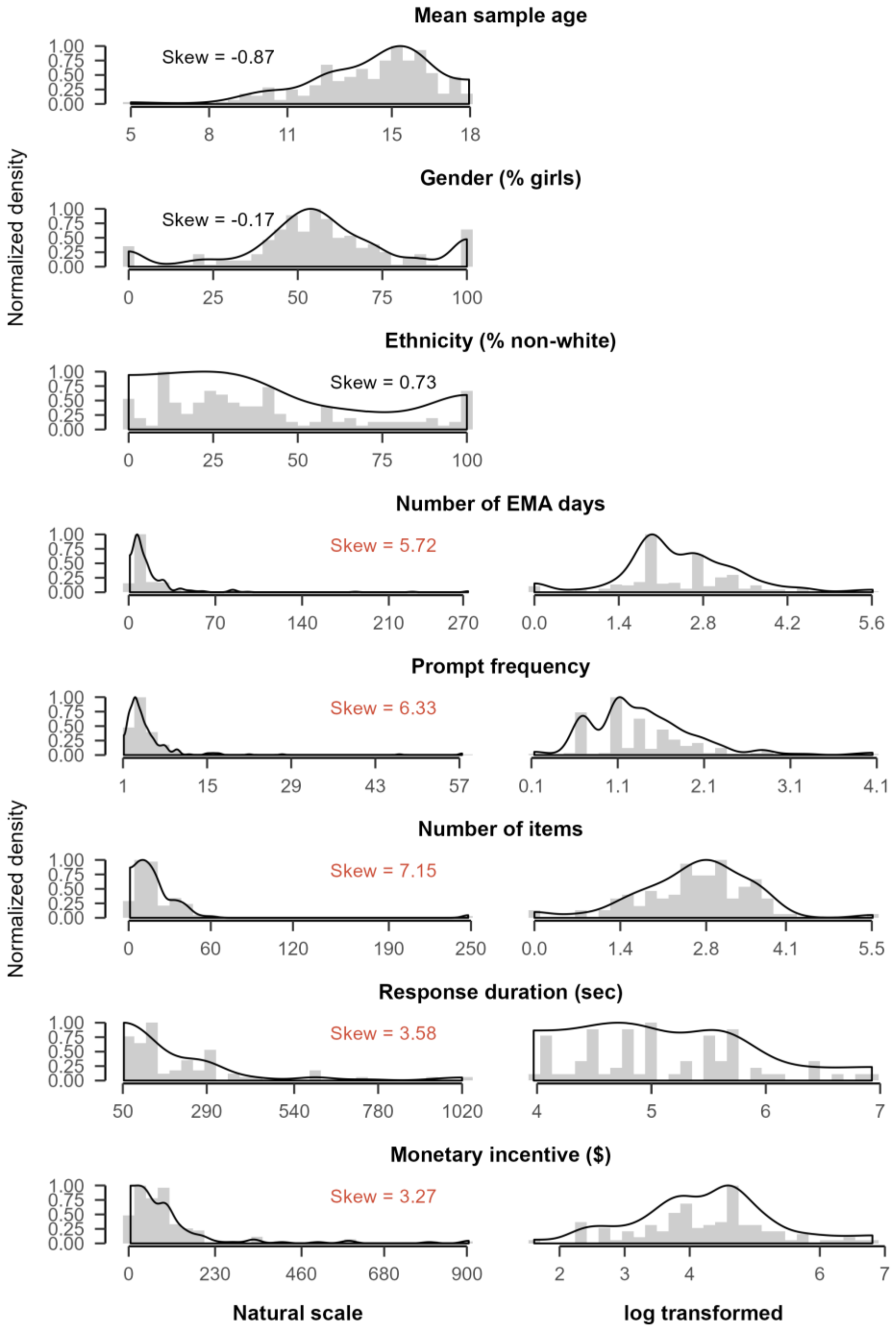

Note: Variables that expressed skew above 3 were log-transformed.

Associations among Included Predictor Variables

Figure S2. Correlation matrix of included continuous predictor variables

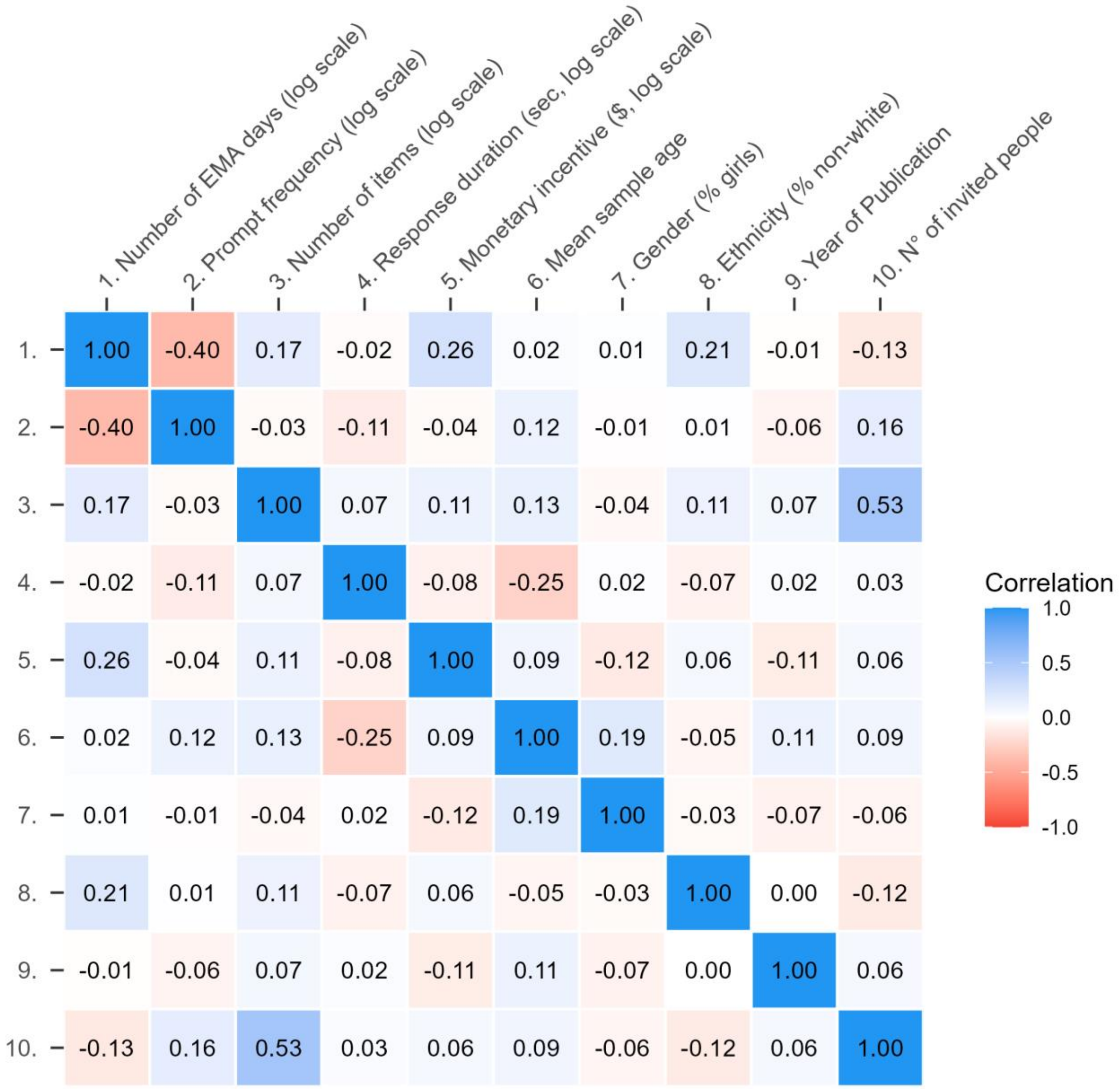

Figure S3. Correlation of study length and prompt frequency.

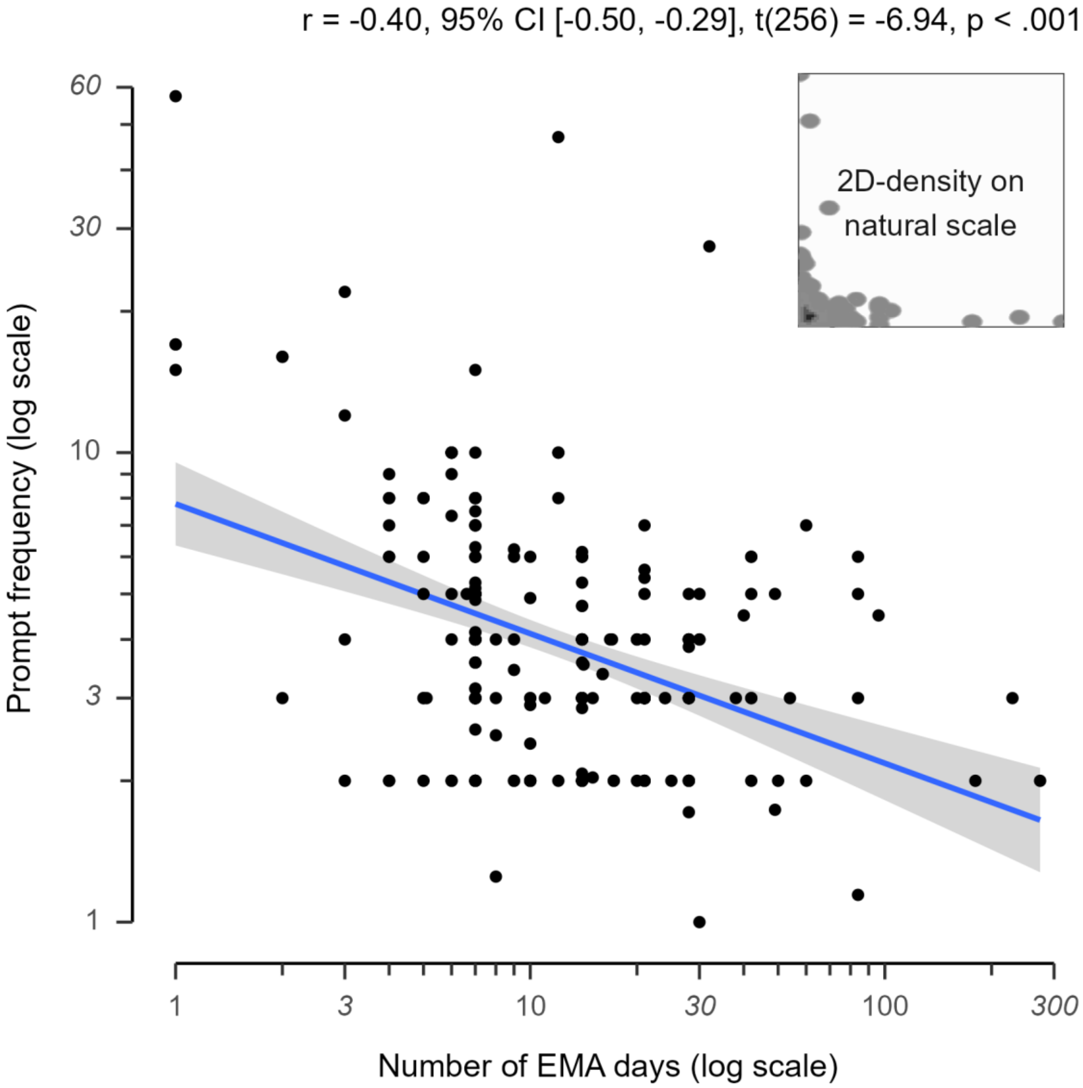

Missing Data Patterns

Figure S4.1. Missingness clustered by reported acceptance

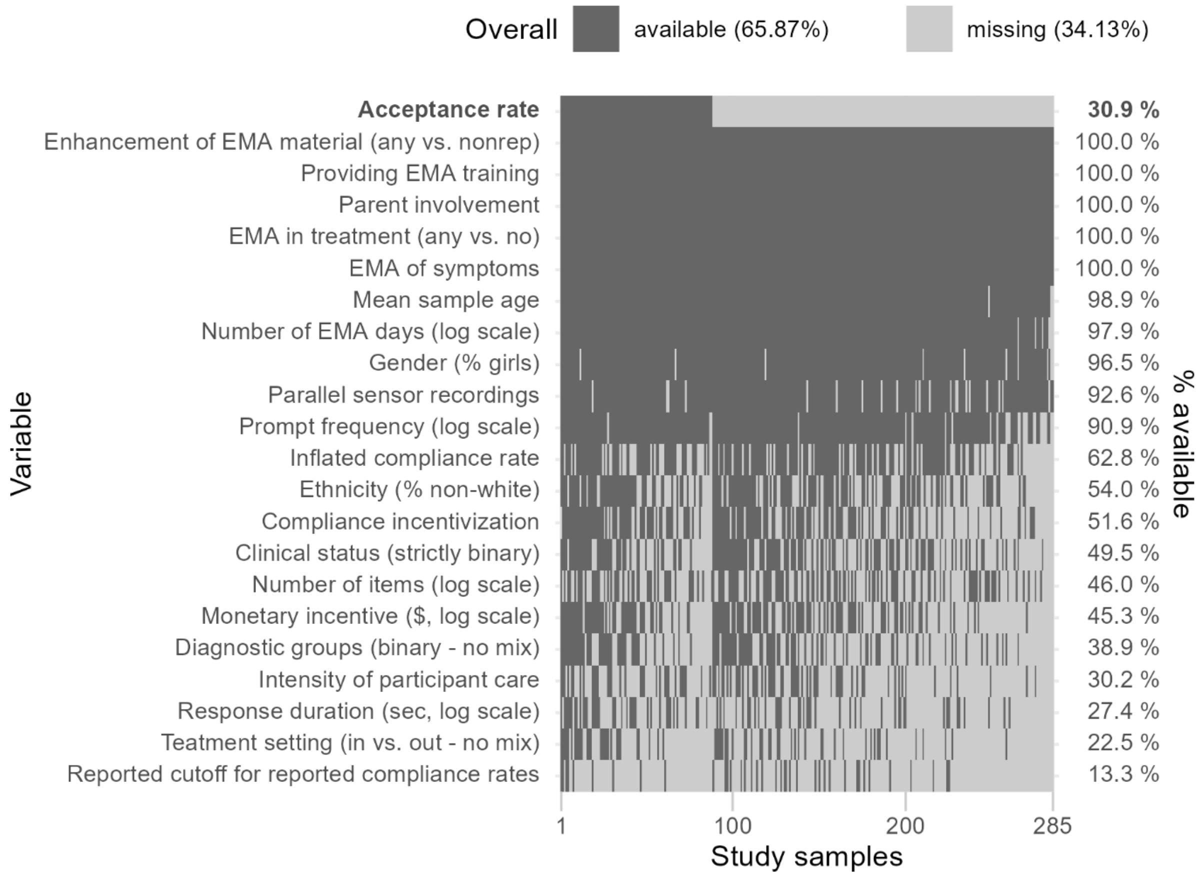

Figure S4.2. Missingness clustered by reported compliance

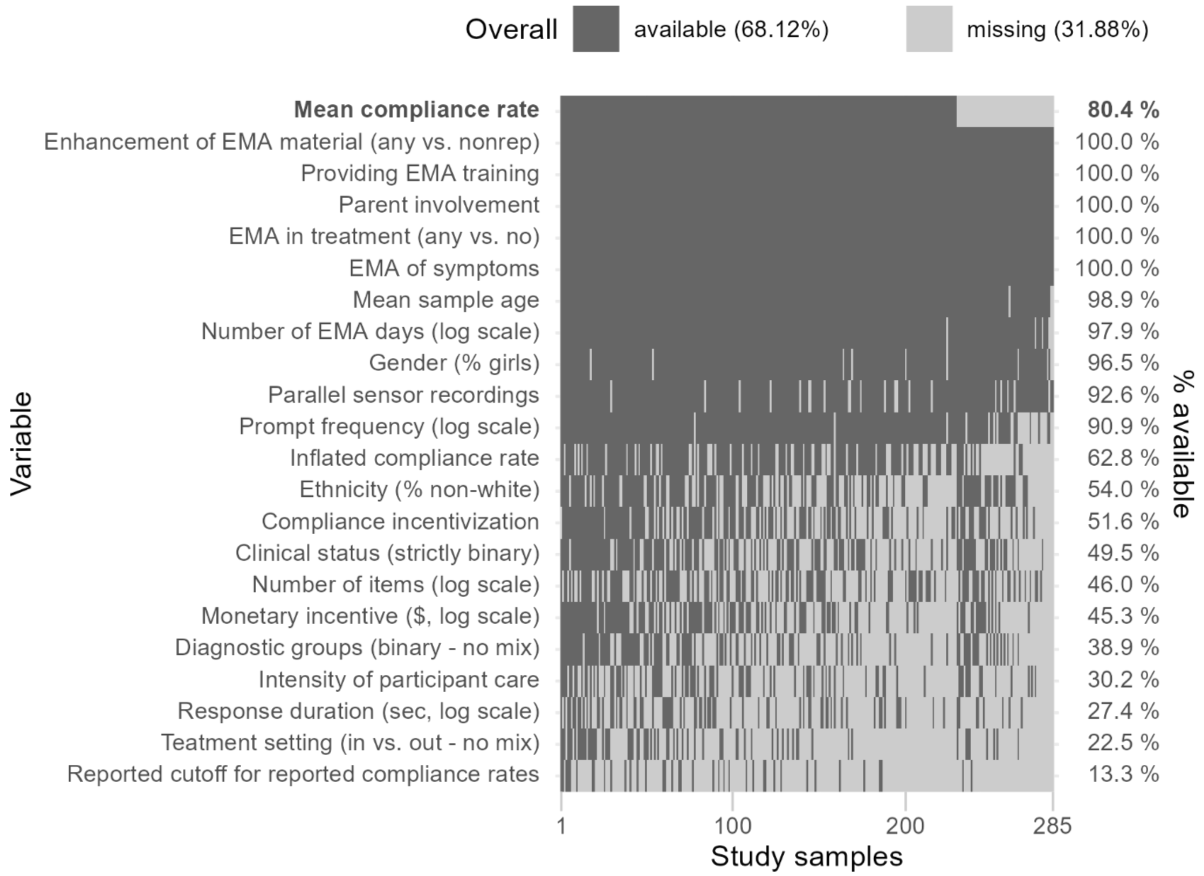

Figure S4.3. Missingness clustered by reported retention

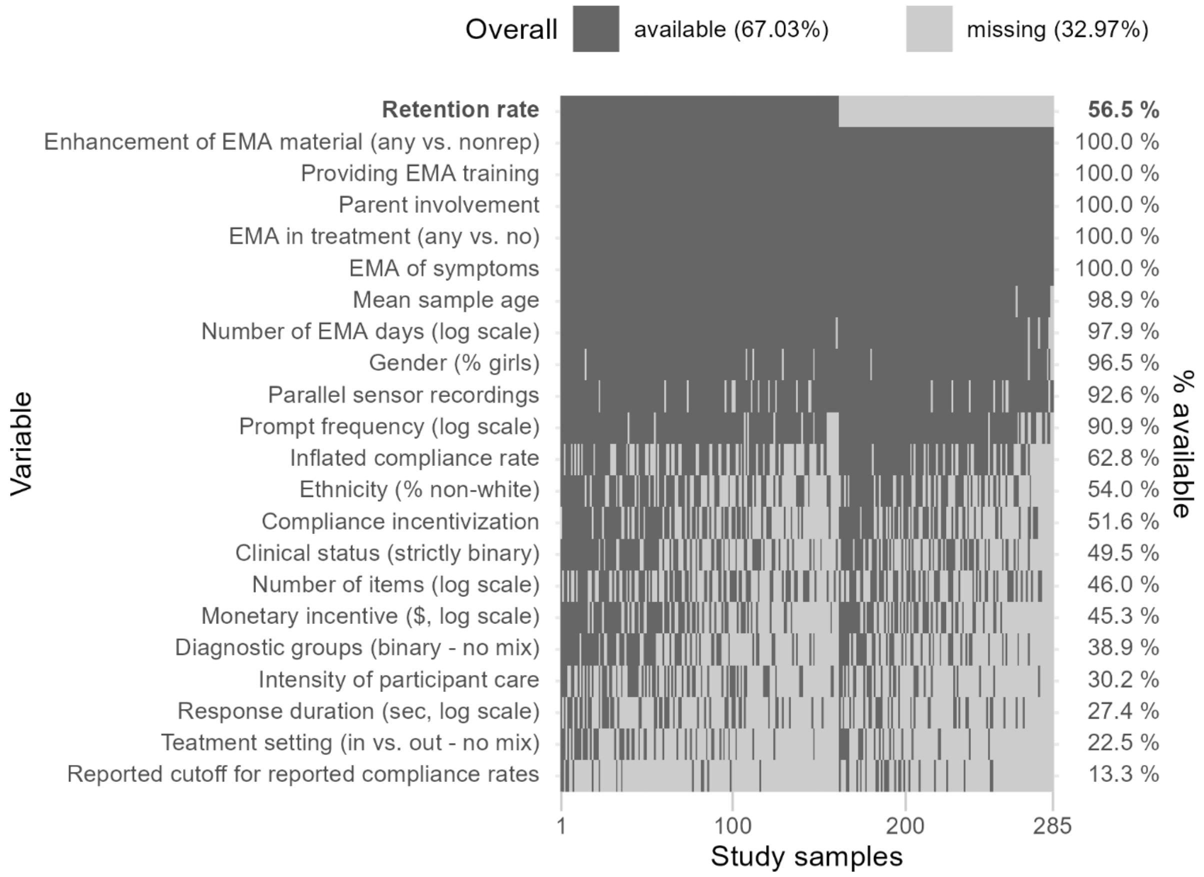

Figure S5. Distributions participation metrics

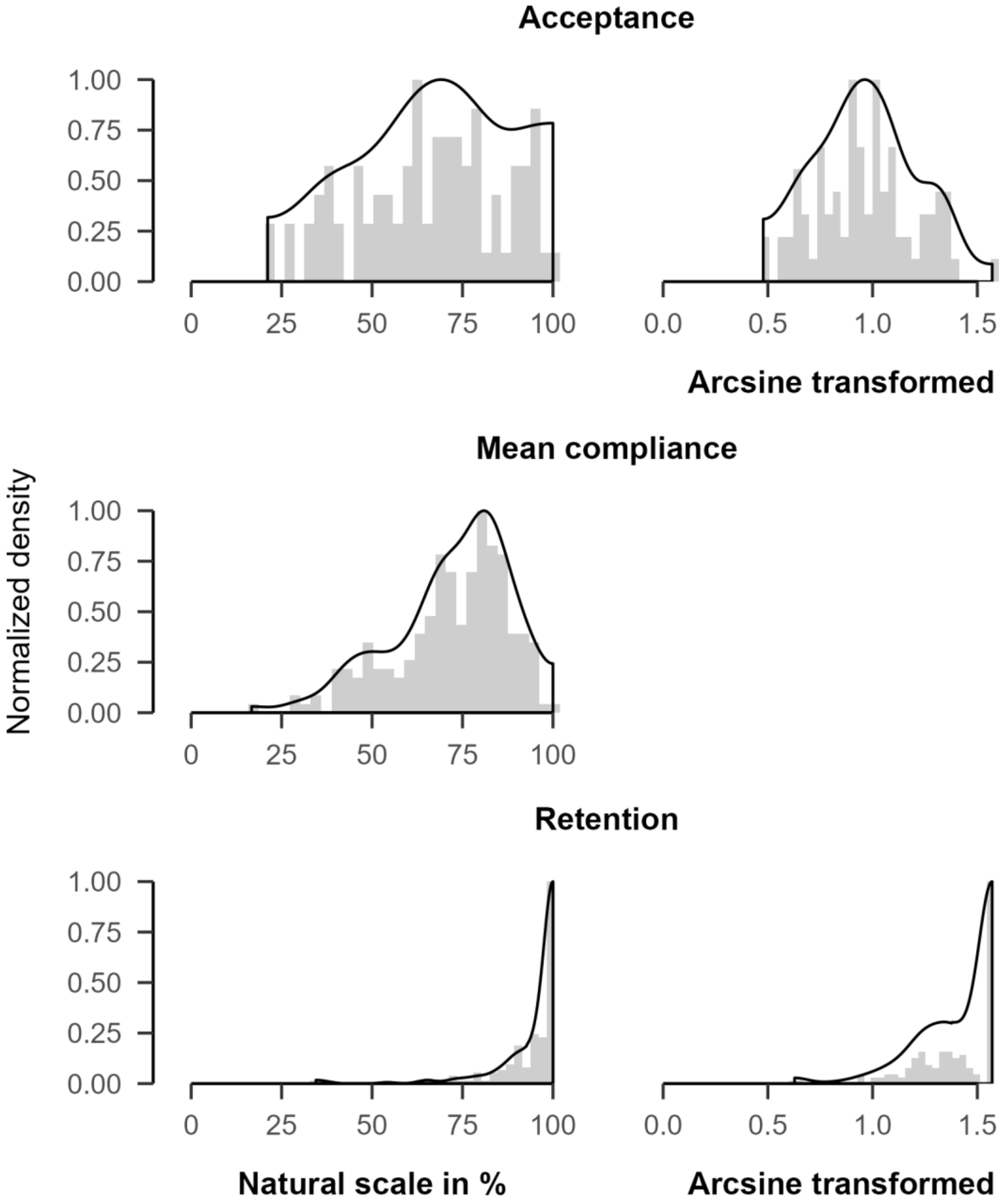

Meta-analyses of Acceptance, Retention, and Compliance

Distribution of effect size estimates

Figure S6.1. Caterpillar plot of acceptance estimates

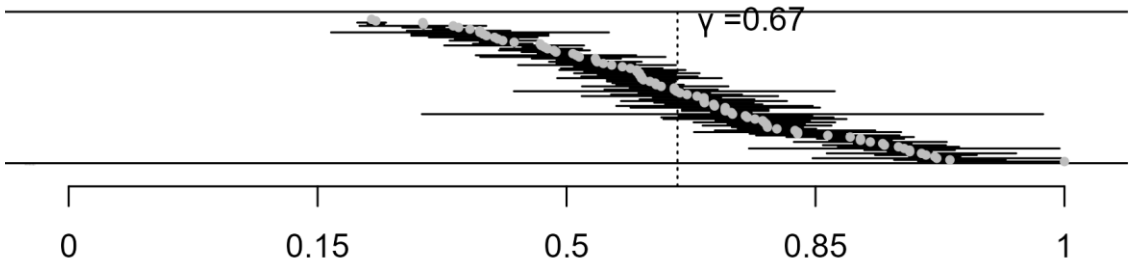

Figure S6.2. Caterpillar plot of compliance estimates

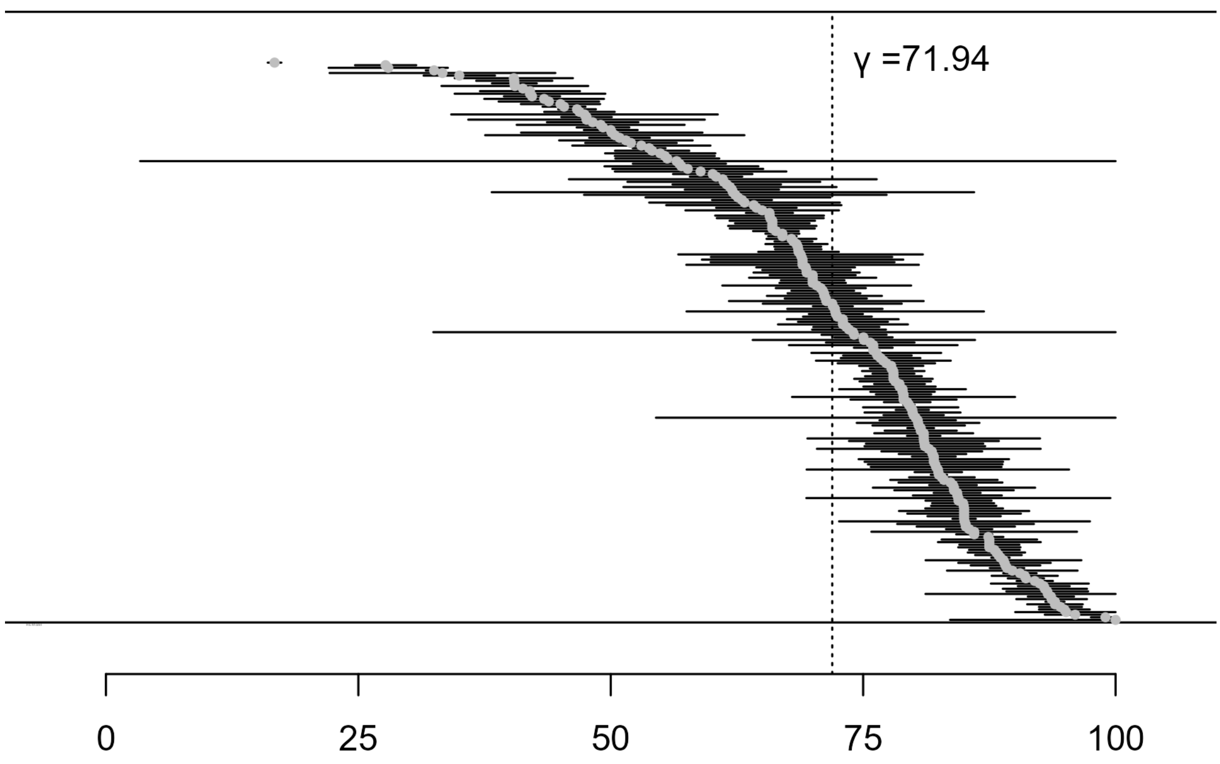

Figure S6.3. Caterpillar plot of retention estimates

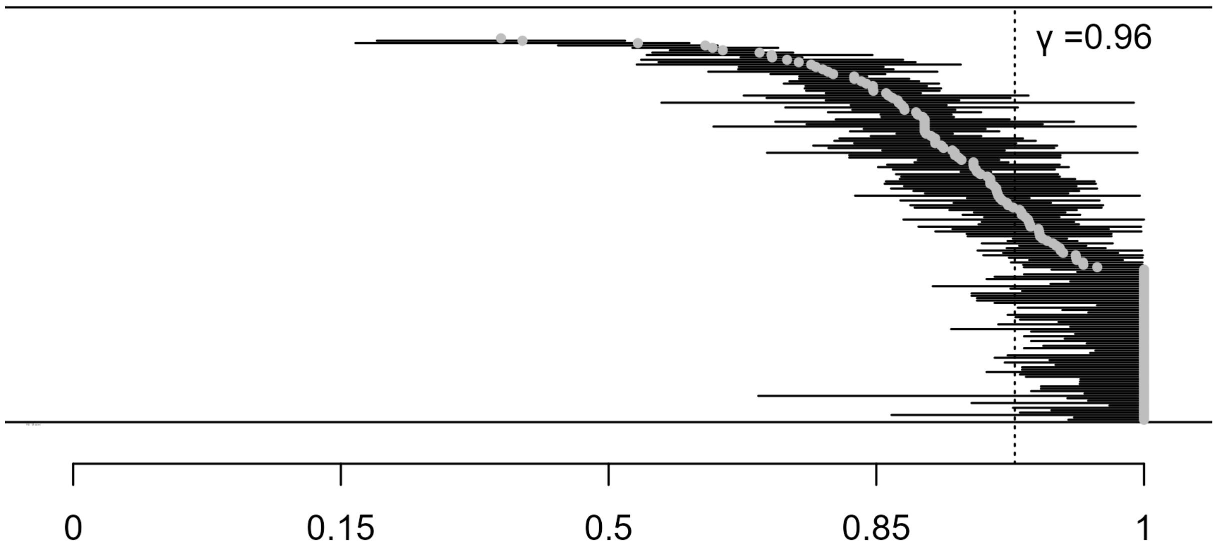

Influential Samples

Initial random-effects models estimated an average acceptance rate of 67.15% (95%CI [62.32; 71.8]), an average compliance rate of 71.94% (95%CI [69.8; 74.08]), and an average retention of 96.45% (95%CI [95.25; 97.48]). Three samples with low compliance rates (16.7% - 27.96%) [1–3] and three with low retention rates (34.48% - 54.29%) [4–6] were highly influential based on their studentized residuals and Cook's distances (see **Figure S7.1-3.**). However, we followed recommendations [7] we abstained from the exclusion of influential samples if they reflect plausible influence from planned moderators. Hence, we retained five samples [1–3,5,6] with intensive protocols (assessment days: 42 - 229; questionnaires: 53) to maintain the integrity of moderator analysis. The remaining study [4] with a low retention rate (37.5%) required participants to regularly send pain reports to the study physician via e-mail, thus, demanding significant proactive engagement beyond the app-based EMA collection. We excluded this study from further analysis since supplementary engagement demands were beyond the scope of the planned moderator analyses. After removal, average rates increased marginally for acceptance 67.27% (95%CI [62.39; 71.96), compliance 71.97% (95%CI [69.83; 74.11]), and retention 96.57% (95%CI [95.42; 97.56]; see **Figure S6.1-3** for caterpillar plots). Sensitivity analysis on the exclusion of all six influential samples showed gains of less than one percent (see **Table S2.1**).

References

1. Holton NS. *Eudaimonia and Engagement in the Classroom: Using Experience Sampling in an Exploratory Study of Well-Being in High School Students*. 2017. doi:10.25335/b1ea-sg35

2. Bentley KH, Millner AJ, Bear A, et al. Intervening on high-risk responses during ecological momentary assessment of suicidal thoughts: Is there an effect on study data? *Psychological Assessment*. 2024;36(1):66-80. doi:10.1037/pas0001288

3. Dietvorst E, Aukes MA, Legerstee JS, et al. A Smartphone Serious Game for Adolescents (Grow It! App): Development, Feasibility, and Acceptance Study. *JMIR Formative Research*. 2022;6(3):e29832. doi:10.2196/29832

4. Tutelman PR, Chambers CT, Stinson JN, et al. The Implementation Effectiveness of a Freely Available Pediatric Cancer Pain Assessment App: A Pilot Implementation Study. *JMIR Cancer*. 2018;4(2):e10280. doi:10.2196/10280

5. Bakshi N, Smith ME, Ross D, Krishnamurti L. Novel Metrics in the Longitudinal Evaluation of Pain Data in Sickle Cell Disease. *The Clinical Journal of Pain*. 2017;33(6):517. doi:10.1097/ajp.0000000000000431

6. Buhr L, Moschko T, Eppinger Ruiz de Zarate A, Schwarz U, Kühnhausen J, Gawrilow C. The Association of Self-Reported ADHD Symptoms and Sleep in Daily Life of a General Population Sample of School Children: An Inter- and Intraindividual Perspective. *Brain Sciences*. 2022;12(4):440. doi:10.3390/brainsci12040440

7. Viechtbauer W, Cheung MW-L. Outlier and influence diagnostics for meta-analysis. *Research Synthesis Methods*. 2010;1(2):112-125. doi:10.1002/jrsm.11

Figure S7.1. Studentized residuals and Cook's D scores for acceptance

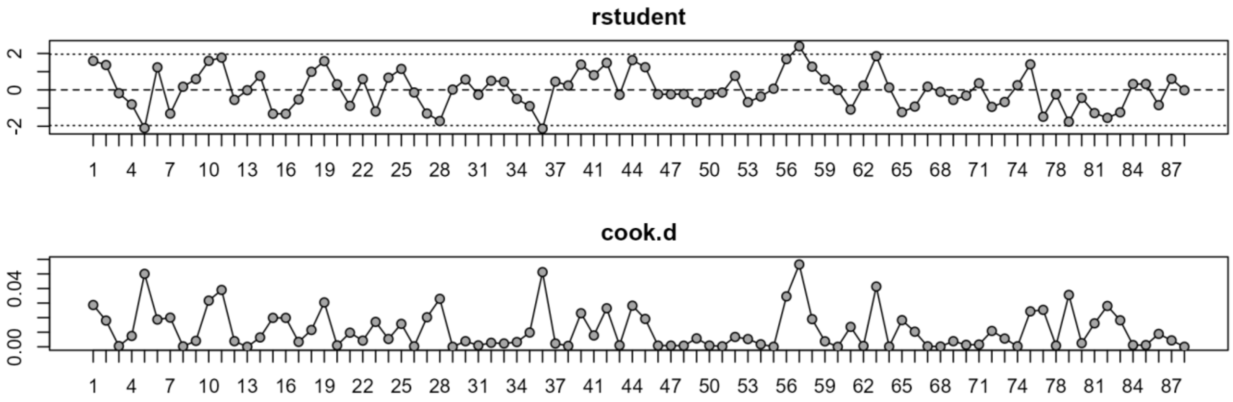

Figure S7.2. Studentized residuals and Cook's D scores for compliance

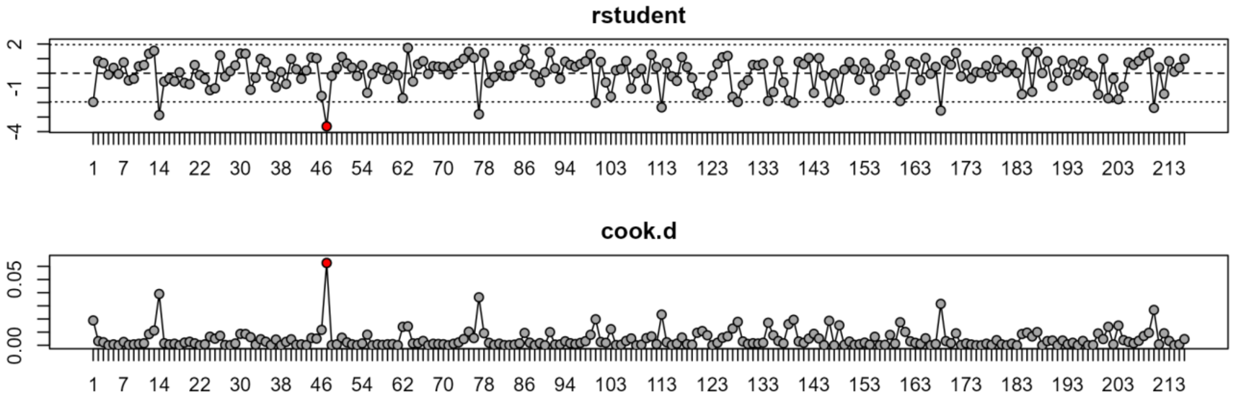

Figure S7.3. Studentized residuals and Cook's D scores for retention

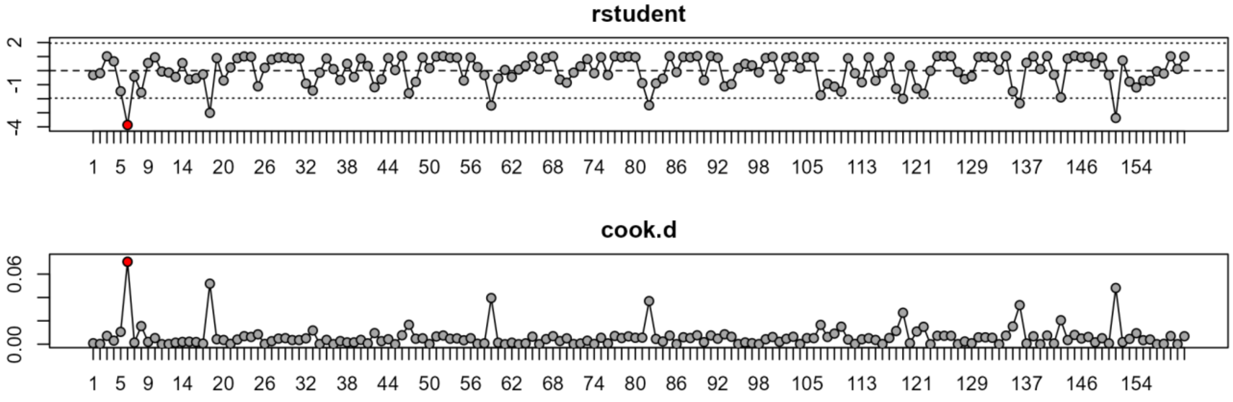

Senstivity Analyses

Table S3.1. Sensitivity to three-level meta-analysis and outlier removal

Model	k	g	Estimate±SE	δ	95% CI	τ^2±SE (σ2.1; σ2.2)	QE
**Acceptance**							
Inclusive	86	-	67.1 ± 0.1	-	[62.3; 71.8]	0.054 ± 0.009	8,395
Three-level model	86	82	67.2 ± 0.1	< ±0.001	[62.2; 72.1]	(0.055; 0.000)	8,395
Remove one influential sample	85	-	67.3 ± 0.1	< ±0.001	[62.4; 72.0]	0.055 ± 0.009	8,393
Remove all six influential samples	84	-	67.5 ± 0.1	+0.001	[62.6; 72.2]	0.055 ± 0.009	8,393
**Compliance**							
Inclusive	216	-	71.9 ± 1.1	-	[69.8; 74.1]	0.025 ± 0.002	62,061
Three-level model	216	200	71.7 ± 1.1	−0.3	[69.4; 73.9]	(0.022; 0.003)	62,061
Remove one influential sample	215	-	72.0 ± 1.1	+0.03	[69.8; 74.1]	0.025 ± 0.002	62,060
Remove all six influential samples	210	-	72.8 ± 1.0	+0.8	[70.7; 74.8]	0.021 ± 0.002	33,396
**Retention**							
Inclusive	160	-	96.5 ± 0.0	-	[95.3; 97.5]	0.032 ± 0.004	1,981
Three-level model	160	153	96.3 ± 0.0	0	[95.1; 97.4]	(0.033; 0.000)	1,981
Remove one influential sample	159	-	96.6 ± 0.0	+0.001	[95.4; 97.6]	0.031 ± 0.004	1,950
Remove all six influential samples	156	-	96.8 ± 0.0	+0.01	[95.8; 97.7]	0.026 ± 0.003	1,808

Note: δ quantifies the differences in pooled estimates between the inclusive and the respective alternative models. σ2.1 and σ2.2 quantify first and second order residual heterogeneity, respectively.

Table S3.2 Model comparisons across higher-order models

Model	df	AIC	AICc	BIC	logLik	LRT	P-value	QE	Variance components (L1 + L2 + L3; in %)
**Acceptance**									
Three-level	3	−5.85	−5.55	1.48	5.92	—	—	8,395.20	(1.42 + 0.00 + 98.58)
Two-level	2	2.27	2.42	7.15	0.87	10.12	0.001	8,395.20	
**Compliance**									
Three-level	3	−187.76	−187.65	−177.65	96.88	—	—	62,060.60	(0.52 + 13.01 + 86.47)
Two-level	2	−174.42	−174.36	−167.68	89.21	15.34	<0.001	62,060.60	
**Retention**									
Three-level	3	−71.48	−71.33	−62.27	38.74	—	—	1,980.97	(6.66 + 0.00 + 93.34)
Two-level	2	−62.29	−62.21	−56.15	33.14	11.19	<0.001	1,980.97	

E9 Publication Bias

Figure S8.1. Funnel plot of acceptance and sample size

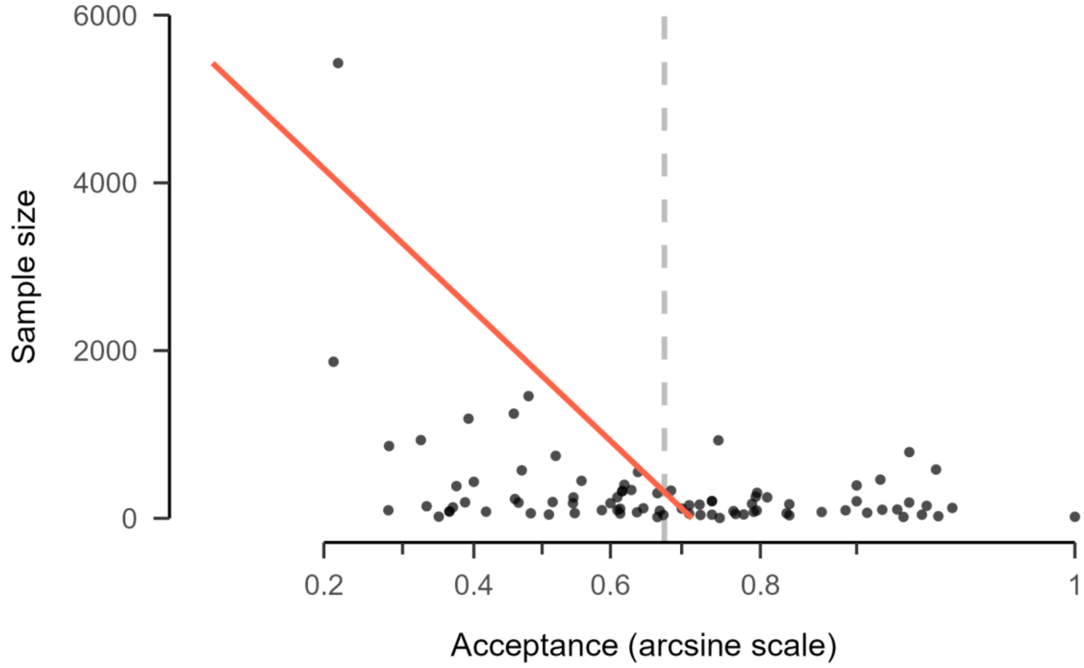

Figure S8.2. Funnel plot of compliance rates and sample size

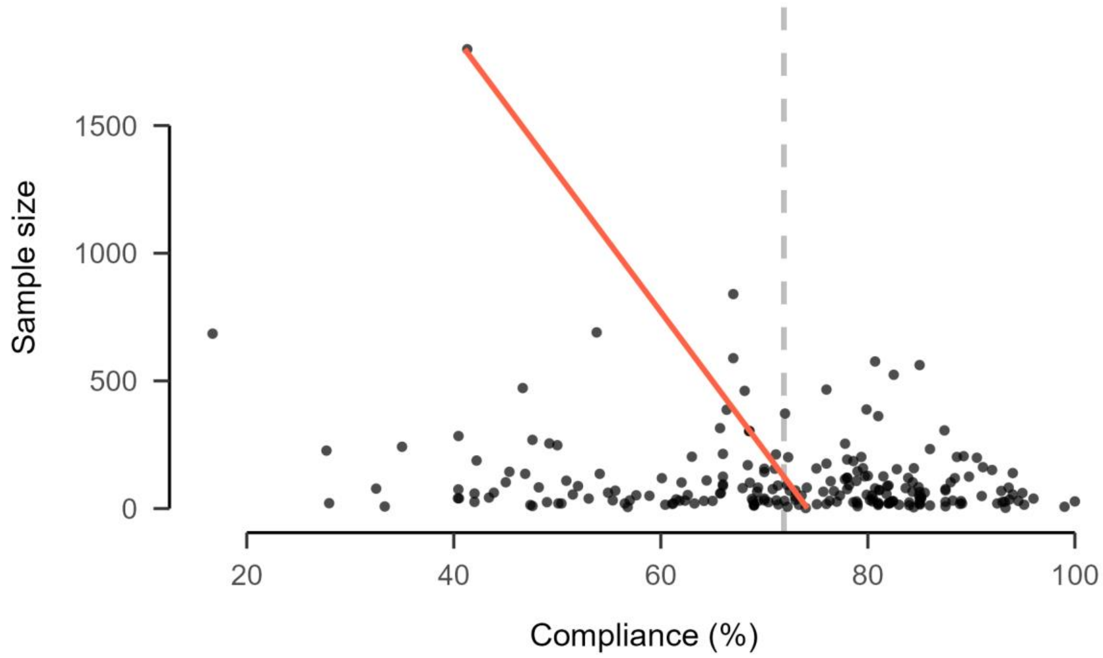

Figure S8.3. Funnel plot of retention and sample size

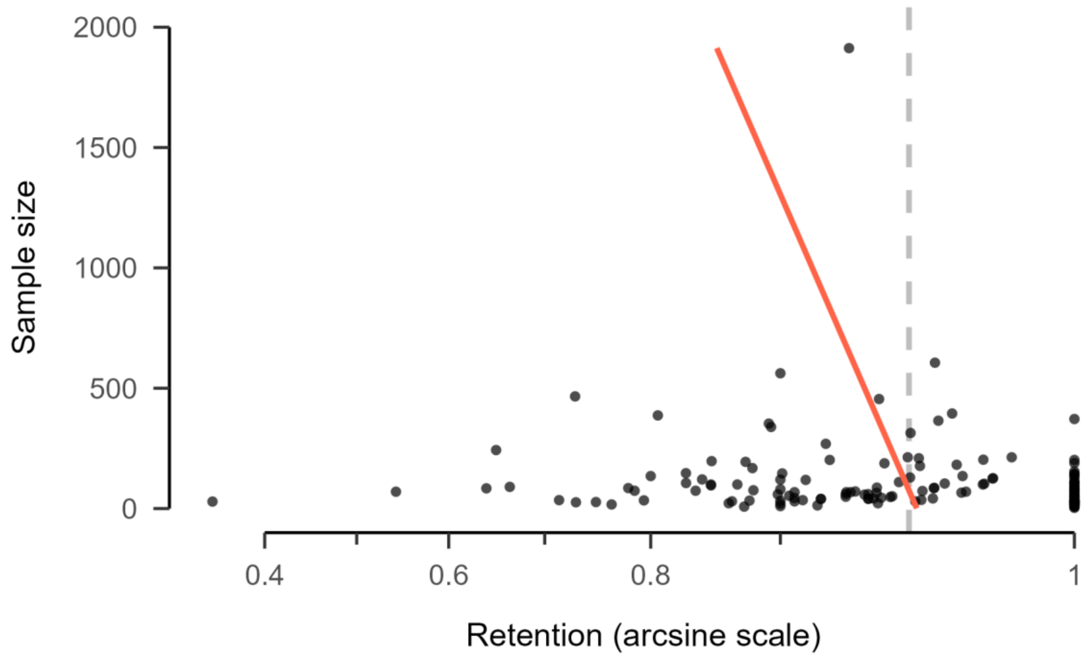

Meta-regression results

Simple Regressions

Table S4.1. Sample characteristics predicting acceptance

Characteristic	k	Estimate ± SE	95% CI	*P*-value	Q_M test (*df*)	*P*-value	R^2
Mean sample age	85						0.00
Intercept		1.11±0.19	[0.75;1.48]	—			
Beta		-0.01±0.01	[-0.04;0.01]	0.415			
Gender (% girls)	83						5.24
Intercept		1.17±0.09	[0.99;1.36]	—			
Beta		0±0	[-0.01;0]	0.016			
Ethnicity (% non-white)	52						1.05
Intercept		1.02±0.06	[0.91;1.13]	—			
Beta		0±0	[0;0]	0.207			
Diagnostic groups (binary - no mix)	39				2.01 (1)	0.156	4.02
Intercept (somatic)	23	0.93±0.04	[0.84;1.01]	—			
Beta (psychiatric)	17	0.09±0.06	[-0.04;0.22]	0.156			
EMA in treatment (any vs. no)	85				0.13 (1)	0.720	0.00
Intercept (unrelated)	62	0.96±0.03	[0.9;1.02]	—			
Beta (pre, peri, or post-	25	0.02±0.06	[-0.09;0.14]	0.720			
EMA of symptoms	85				0.04 (1)	0.842	0.00
Intercept (no)	58	0.97±0.03	[0.9;1.03]	—			
Beta (yes)	29	-0.01±0.06	[-0.12;0.1]	0.842			

Note: Parameters are presented on the arcsine scale. Unlisted predictors are dropped due to less than 40 available studies per predictor, or less than 10 per category.

Table S4.2. Design characteristics predicting acceptance

Characteristic	k	Estimate ± SE	95% CI	*P*-value	Q_M test (*df*)	*P*-value	R^2
Number of EMA days (log scale)	85						1.43
Intercept		1.09±0.09	[0.92;1.26]	—			
Beta		-0.05±0.03	[-0.11;0.01]	0.115			
Prompt frequency (log scale)	82						0.00
Intercept		0.91±0.07	[0.78;1.05]	—			
Beta		0.03±0.04	[-0.06;0.12]	0.509			
Number of items (log scale)	41						19.98
Intercept		1.24±0.11	[1.03;1.45]	—			
Beta		-0.12±0.04	[-0.19;-0.05]	0.001			
Enhancement of EMA material (any vs.	85				2.18 (1)	0.140	1.17
Intercept (no or not reported)	77	0.97±0.03	[0.92;1.03]	—			
Beta (visual enhancement or	10	-0.12±0.08	[-0.29;0.04]	0.140			
Parallel sensor recordings	81				0.38 (1)	0.540	0.00
Intercept (no add-on)	53	0.97±0.03	[0.91;1.04]	—			
Beta (add-on sensors)	30	-0.03±0.06	[-0.14;0.08]	0.540			
Compliance incentivization	56				1.19 (1)	0.275	0.28
Intercept (no compliance	23	0.91±0.05	[0.81;1.01]	—			
Beta (compliance incentivization)	34	0.07±0.07	[-0.06;0.2]	0.275			
Monetary incentive ($, log scale)	47						0.00
Intercept		0.86±0.15	[0.58;1.15]	—			
Beta		0.02±0.04	[-0.05;0.09]	0.542			
Providing EMA training	85				1.16 (1)	0.282	0.28
Intercept (no or not reported)	25	0.92±0.05	[0.82;1.01]	—			
Beta (yes)	62	0.06±0.06	[-0.05;0.17]	0.282			

Note: Parameters are presented on the arcsine scale. Unlisted predictors are dropped due to less than 40 available studies per predictor, or less than 10 per category.

Table S4.3. Sample characteristics predicting compliance

Characteristic	k	Estimate ± SE	95% CI	*P*-value	Q_M test (*df*)	*P*-value	R^2
Mean sample age	215						0.29
Intercept		80.73±7.19	[66.64;94.81]	—			
Beta		-0.62±0.5	[-1.59;0.36]	0.218			
Gender (% girls)	210						0.39
Intercept		75.5±2.92	[69.79;81.22]	—			
Beta		-0.06±0.05	[-0.16;0.03]	0.207			
Ethnicity (% non-white)	116						1.39
Intercept		75.52±2.31	[70.99;80.06]	—			
Beta		-0.08±0.05	[-0.17;0.02]	0.102			
Clinical status (strictly binary)	110				0.97 (1)	0.324	<0.01
Intercept (clinical)	84	72.12±1.71	[68.77;75.47]	—			
Beta (healthy controls)	26	3.4±3.45	[-3.36;10.17]	0.324			
Diagnostic groups (binary - no mix)	83				0.01 (1)	0.922	0.00
Intercept (somatic)	39	72.03±2.54	[67.06;77.01]	—			
Beta (psychiatric)	44	0.34±3.46	[-6.45;7.13]	0.922			
EMA in treatment (any vs. no)	215				0.00 (1)	0.998	0.00
Intercept (unrelated)	160	71.97±1.26	[69.5;74.44]	—			
Beta (pre, peri, or post-treatment)	55	-0.01±2.54	[-4.99;4.98]	0.998			
Teatment setting (in vs. out - no mix)	54				0.04 (1)	0.844	0.00
Intercept (inpatient)	13	70.64±4.89	[61.06;80.23]	—			
Beta (outpatient)	41	1.1±5.61	[-9.89;12.09]	0.844			
EMA of symptoms	215				1.39 (1)	0.238	0.13
Intercept (no)	154	72.77±1.28	[70.25;75.28]	—			
Beta (yes)	61	-2.89±2.45	[-7.68;1.91]	0.238			

Table S4.4. Design characteristics predicting compliance

Characteristic	k	Estimate ± SE	95% CI	*P*-value	Q_M test (*df*)	*P*-value	R^2
Number of EMA days (log scale)	215						0.16
Intercept		75.91±3.48	[69.08;82.73]	—			
Beta		-1.63±1.37	[-4.32;1.05]	0.234			
Prompt frequency (log scale)	213						1.09
Intercept		76.46±2.84	[70.89;82.04]	—			
Beta		-3.31±1.85	[-6.94;0.32]	0.074			
Number of items (log scale)	100						1.18
Intercept		78.99±5.59	[68.03;89.95]	—			
Beta		-2.86±1.98	[-6.75;1.03]	0.149			
Response duration (sec, log scale)	67						8.38
Intercept		32.39±15.4	[2.21;62.56]	—			
Beta		7.72±2.97	[1.91;13.53]	0.009			
Enhancement of EMA material (any vs.	215				0.20 (1)	0.655	0.00
Intercept (no or not reported)	188	71.79±1.17	[69.49;74.08]	—			
Beta (visual enhancement or	27	1.49±3.33	[-5.04;8.02]	0.655			
Parallel sensor recordings	200				0.06 (1)	0.805	0.00
Intercept (no add-on)	146	70.87±1.34	[68.24;73.49]	—			
Beta (add-on sensors)	54	0.63±2.56	[-4.39;5.65]	0.805			
Compliance incentivization	118				1.67 (1)	0.196	0.58
Intercept (no compliance incentivization)	44	71.29±2.26	[66.87;75.71]	—			
Beta (compliance incentivization)	74	3.69±2.85	[-1.9;9.28]	0.196			
Monetary incentive ($, log scale)	105						1.36
Intercept		65.95±6.09	[54.01;77.89]	—			
Beta		2.04±1.39	[-0.68;4.77]	0.141			
Providing EMA training	215				6.83 (1)	0.009	2.75
Intercept (no or not reported)	72	67.97±1.87	[64.29;71.64]	—			
Beta (yes)	143	5.99±2.29	[1.5;10.48]	0.009			
Intensity of participant care	66				1.63 (1)	0.202	1.20
Intercept (minimal contact)	22	71.03±3.19	[64.78;77.28]	—			
Beta (active contact)	44	5±3.91	[-2.67;12.67]	0.202			
Parent involvement	215				7.39 (3)	0.060	2.08
Intercept (no involvement)	158	70.83±1.27	[68.35;73.31]	—			
Beta (some parent reports)	18	2.56±3.91	[-5.11;10.23]	0.513			
Beta (parents assist child EMA)	22	0.58±3.6	[-6.48;7.65]	0.872			
Beta (parallel parent EMA)	17	10.85±4.04	[2.93;18.77]	0.007			

Table S4.5. Sample characteristics predicting retention

Characteristic	k	Estimate ± SE	95% CI	*P*-value	Q_M test (*df*)	*P*-value	R^2
Mean sample age	159						0.36
Intercept		1.49±0.09	[1.31;1.67]	—			
Beta		-0.01±0.01	[-0.02;0.01]	0.247			
Gender (% girls)	154						0.00
Intercept		1.34±0.04	[1.26;1.43]	—			
Beta		0±0	[0;0]	0.367			
Ethnicity (% non-white)	88						4.99
Intercept		1.42±0.03	[1.36;1.49]	—			
Beta		0±0	[0;0]	0.038			
Clinical status (strictly binary)	85				2.67 (1)	0.102	2.43
Intercept (clinical)	71	1.38±0.02	[1.33;1.42]	—			
Beta (healthy controls)	15	0.09±0.05	[-0.02;0.2]	0.102			
Diagnostic groups (binary - no mix)	69				4.02 (1)	0.045	5.49
Intercept (somatic)	41	1.4±0.03	[1.34;1.47]	—			
Beta (psychiatric)	29	-0.1±0.05	[-0.19;0]	0.045			
EMA in treatment (any vs. no)	159				0.08 (1)	0.776	0.00
Intercept (unrelated)	115	1.38±0.02	[1.35;1.42]	—			
Beta (pre, peri, or post-	45	0.01±0.03	[-0.06;0.08]	0.776			
EMA of symptoms	159				0.09 (1)	0.769	0.00
Intercept (no)	106	1.39±0.02	[1.35;1.42]	—			
Beta (yes)	54	-0.01±0.03	[-0.07;0.05]	0.769			

Note: Parameters are presented on the arcsine scale. Unlisted predictors are dropped due to less than 40 available studies per predictor, or less than 10 per category.

Table S4.6. Design characteristics predicting retention

Characteristic	k	Estimate ± SE	95% CI	*P*-value	Q_M test (*df*)	*P*-value	R^2
Number of EMA days (log scale)	158						10.06
Intercept		1.53±0.04	[1.46;1.61]	—			
Beta		-0.06±0.02	[-0.09;-0.03]	<0.001			
Prompt frequency (log scale)	145						0.00
Intercept		1.35±0.04	[1.28;1.43]	—			
Beta		0.02±0.03	[-0.03;0.07]	0.374			
Number of items (log scale)	79						0.45
Intercept		1.46±0.06	[1.34;1.58]	—			
Beta		-0.02±0.02	[-0.07;0.02]	0.266			
Response duration (sec, log scale)	49						1.67
Intercept		1.2±0.16	[0.89;1.51]	—			
Beta		0.04±0.03	[-0.02;0.1]	0.186			
Enhancement of EMA material (any vs.	159				0.65 (1)	0.419	0.00
Intercept (no or not reported)	135	1.38±0.02	[1.35;1.41]	—			
Beta (visual enhancement or	25	0.03±0.04	[-0.05;0.12]	0.419			
Parallel sensor recordings	146				0.03 (1)	0.855	0.00
Intercept (no add-on)	94	1.38±0.02	[1.34;1.42]	—			
Beta (add-on sensors)	53	0.01±0.03	[-0.06;0.07]	0.855			
Compliance incentivization	84				0.56 (1)	0.453	0.00
Intercept (no compliance	33	1.38±0.04	[1.31;1.45]	—			
Beta (compliance incentivization)	52	-0.03±0.05	[-0.13;0.06]	0.453			
Monetary incentive ($, log scale)	78						0.00
Intercept		1.33±0.11	[1.12;1.54]	—			
Beta		0±0.03	[-0.05;0.05]	0.928			
Providing EMA training	159				1.06 (1)	0.302	0.04
Intercept (no or not reported)	51	1.36±0.03	[1.31;1.41]	—			
Beta (yes)	109	0.03±0.03	[-0.03;0.1]	0.302			
Intensity of participant care	58				0.22 (1)	0.638	0.00
Intercept (minimal contact)	16	1.38±0.05	[1.29;1.47]	—			
Beta (active contact)	43	-0.03±0.05	[-0.13;0.08]	0.638			
Parent involvement	159				4.81 (3)	0.186	1.95
Intercept (no involvement)	115	1.39±0.02	[1.35;1.42]	—			
Beta (some parent reports)	14	0.07±0.05	[-0.03;0.17]	0.167			
Beta (parents assist child EMA)	16	-0.07±0.05	[-0.17;0.04]	0.207			
Beta (parallel parent EMA)	15	-0.05±0.05	[-0.15;0.06]	0.367			

Note: Parameters are presented on the arcsine scale. Unlisted predictors are dropped due to less than 40 available studies per predictor, or less than 10 per category.

Figure S9.1. Meta-regression of acceptance on number of items

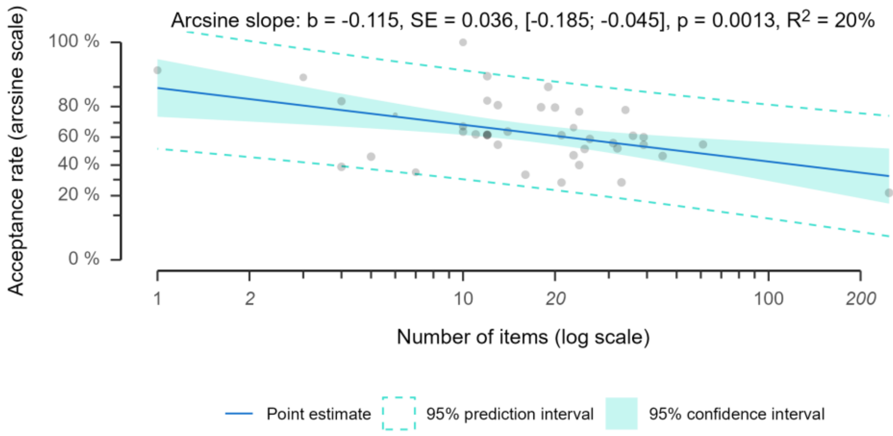

Figure S9.2. Meta-regression of compliance on publication year

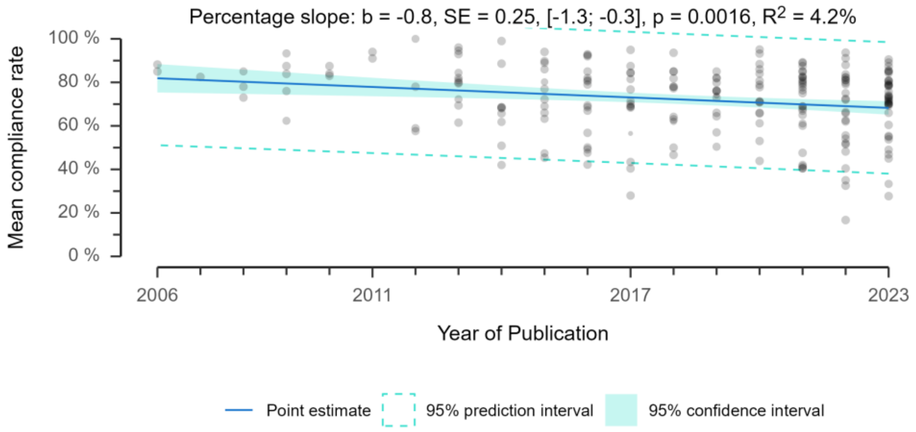

Figure S9.3. Meta-regression of retention on study length

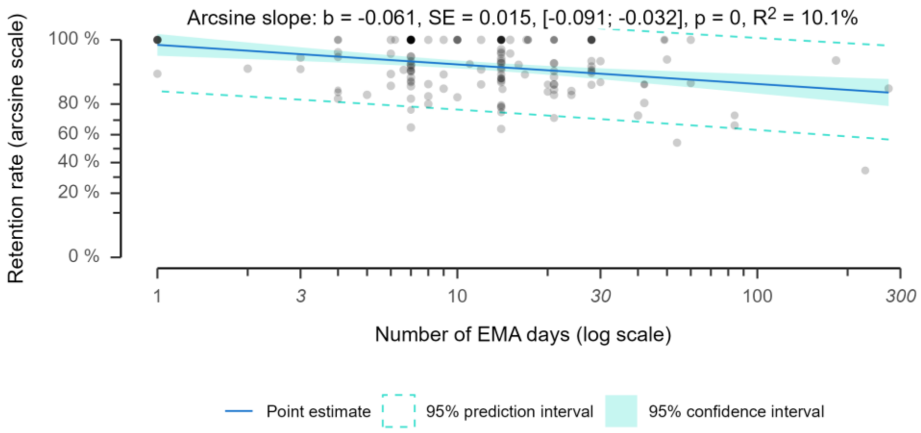

Sensitivity analyses

Figure S10. Meta-regressive effects from studies with uninflated compliance

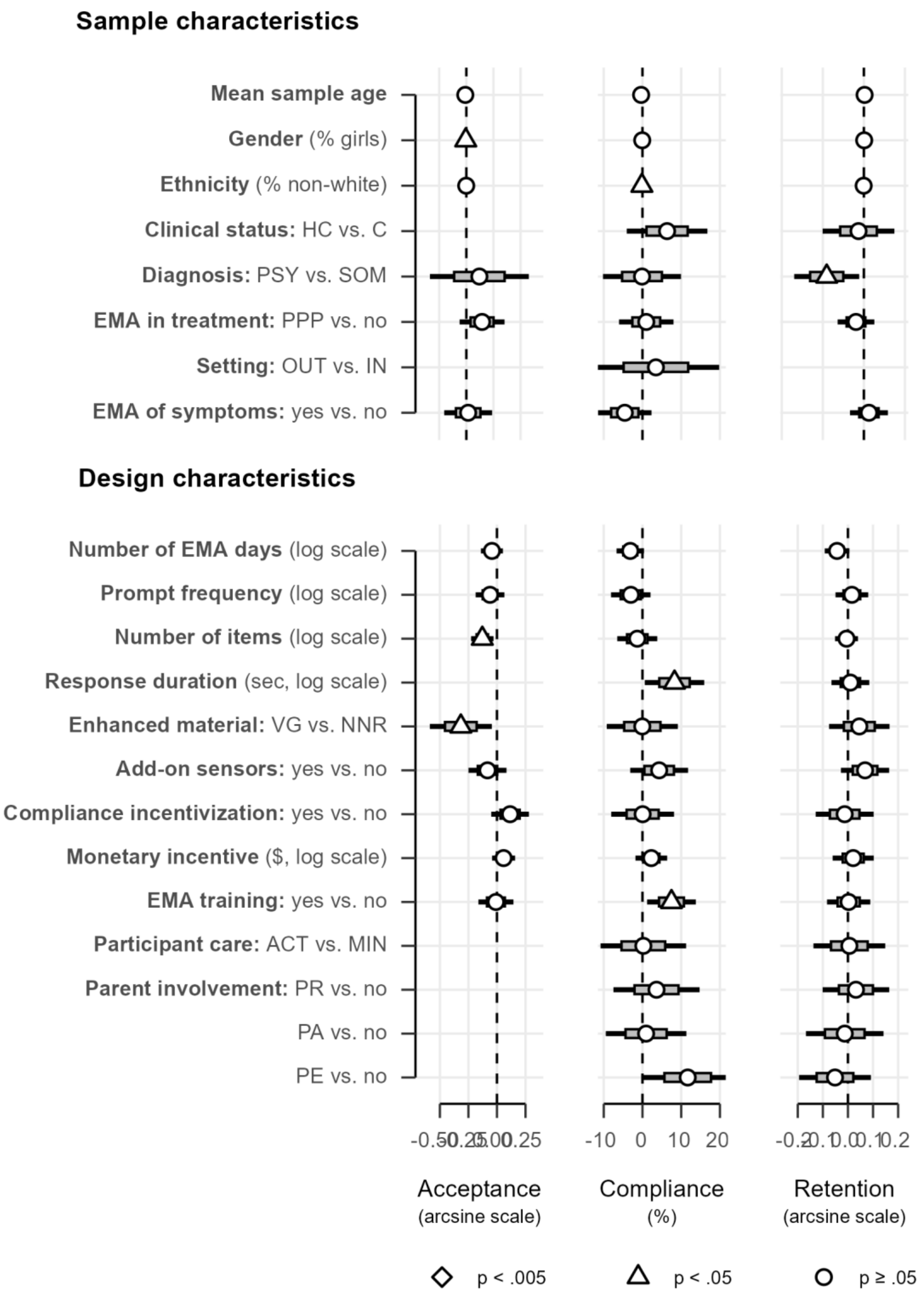

Meta-regressions with interaction terms

Table S5.1. Interaction models predicting acceptance

Characteristic	k	Estimate ± SE	95% CI	*P*-value	Q_M test (*df*)	*P*-value	R^2
Gender * Prompt frequency	80				8.48 (3)	0.037	6.12
Intercept		1.55±0.27	[1.01;2.09]	—			
Beta (Gender)		-0.01±0.01	[-0.02;0]	0.023			
Beta (Prompt frequency)		-0.27±0.18	[-0.62;0.08]	0.127			
Interaction		0.01±0	[0;0.01]	0.099			
Gender * Number of EMA days	83				7.45 (3)	0.059	4.63
Intercept		1.12±0.33	[0.47;1.78]	—			
Beta (Gender)		0±0.01	[-0.01;0.01]	0.846			
Beta (Number of EMA days)		0.01±0.12	[-0.22;0.25]	0.906			
Interaction		0±0	[-0.01;0]	0.659			
Gender * Monetary incentive	46				4.21 (3)	0.240	1.89
Intercept		1.05±0.48	[0.11;1.99]	—			
Beta (Gender)		0±0.01	[-0.02;0.01]	0.771			
Beta (Monetary incentive)		0.05±0.13	[-0.2;0.29]	0.699			
Interaction		0±0	[0;0]	0.714			
Ethnicity * Prompt frequency	51				2.04 (3)	0.564	0.00
Intercept		1.13±0.19	[0.76;1.49]	—			
Beta (Ethnicity)		0±0	[-0.01;0.01]	0.512			
Beta (Prompt frequency)		-0.08±0.14	[-0.35;0.18]	0.537			
Interaction		0±0	[-0.01;0.01]	0.746			
Ethnicity * Number of EMA days	52				3.50 (3)	0.321	1.09
Intercept		1.01±0.17	[0.68;1.33]	—			
Beta (Ethnicity)		0±0	[-0.01;0.01]	0.488			
Beta (Number of EMA days)		-0.01±0.06	[-0.12;0.11]	0.931			
Interaction		0±0	[0;0]	0.325			
Mean sample age * Prompt	82				1.26 (3)	0.739	0.00
Intercept		0.74±0.5	[-0.24;1.72]	—			
Beta (Mean sample age)		0.01±0.03	[-0.05;0.08]	0.745			
Beta (Prompt frequency)		0.23±0.31	[-0.38;0.84]	0.465			
Interaction		-0.01±0.02	[-0.05;0.03]	0.525			
Mean sample age * Number of EMA	85				7.58 (3)	0.056	5.00
Intercept		2.2±0.5	[1.21;3.18]	—			
Beta (Mean sample age)		-0.08±0.03	[-0.14;-0.01]	0.025			
Beta (Number of EMA days)		-0.43±0.19	[-0.81;-0.06]	0.023			
Interaction		0.03±0.01	[0;0.05]	0.041			
Mean sample age * Number of items	41				11.39 (3)	0.010	18.61
Intercept		1.69±0.5	[0.72;2.67]	—			
Beta (Mean sample age)		-0.03±0.03	[-0.1;0.04]	0.352			
Beta (Number of items)		-0.22±0.17	[-0.55;0.11]	0.192			
Interaction		0.01±0.01	[-0.02;0.03]	0.512			

Characteristic	k	Estimate ± SE	95% CI	*P*-value	Q_M test (*df*)	*P*-value	R^2
Mean sample age * Monetary	47				0.60 (3)	0.897	0.00
Intercept		1.65±1.65	[-1.59;4.89]	—			
Beta (Mean sample age)		-0.05±0.11	[-0.26;0.16]	0.632			
Beta (Monetary incentive)		-0.16±0.38	[-0.91;0.58]	0.666			
Interaction		0.01±0.03	[-0.04;0.06]	0.623			

Note: Parameters are presented on the arcsine scale. Unlisted predictors are dropped due to less than 40 available studies per predictor, or less than 10 per category.

Table S5.2. Interaction models predicting compliance

Characteristic	k	Estimate ± SE	95% CI	*P*-value	Q_M test (*df*)	*P*-value	R^2
Clinical status * Prompt frequency	108				5.57 (3)	0.134	2.61
Intercept (clinical)	83	79.08±4.55	[70.16;88]	—			
Beta (healthy controls)	25	7.22±9.87	[-12.12;26.56]	0.464			
Beta (Prompt frequency)	83	-5.34±3.13	[-11.48;0.8]	0.088			
Interaction	25	-1.76±5.95	[-13.42;9.91]	0.768			
Clinical status * Number of EMA days	110				3.47 (3)	0.324	0.52
Intercept (clinical)	84	79.33±5.32	[68.9;89.75]	—			
Beta (healthy controls)	26	-9.87±10.9	[-31.24;11.5]	0.365			
Beta (Number of EMA days)	84	-2.87±2	[-6.79;1.06]	0.152			
Interaction	26	5.85±4.87	[-3.71;15.4]	0.230			
Clinical status * Number of items	56				1.40 (3)	0.706	0.00
Intercept (clinical)	46	78.91±7.7	[63.82;94]	—			
Beta (healthy controls)	10	-2.54±16.57	[-35.02;29.93]	0.878			
Beta (Number of items)	46	-3.08±2.86	[-8.68;2.52]	0.281			
Interaction	10	0.99±5.4	[-9.59;11.57]	0.855			
Gender * Prompt frequency	208				4.78 (3)	0.188	1.06
Intercept		73.74±7.59	[58.86;88.61]	—			
Beta (Gender)		0.05±0.13	[-0.21;0.31]	0.702			
Beta (Prompt frequency)		0.27±4.51	[-8.56;9.1]	0.953			
Interaction		-0.06±0.08	[-0.22;0.09]	0.408			
Gender * Number of EMA days	210				3.58 (3)	0.310	0.30
Intercept		73.18±8.67	[56.18;90.18]	—			
Beta (Gender)		0.04±0.13	[-0.22;0.29]	0.777			
Beta (Number of EMA days)		1.09±3.57	[-5.91;8.1]	0.759			
Interaction		-0.04±0.05	[-0.15;0.06]	0.419			
Gender * Number of items	96				1.66 (3)	0.645	0.00
Intercept		89.29±17.01	[55.95;122.62]	—			
Beta (Gender)		-0.21±0.28	[-0.75;0.33]	0.440			
Beta (Number of items)		-5.76±6.25	[-18.02;6.49]	0.357			
Interaction		0.07±0.1	[-0.14;0.27]	0.525			
Gender * Monetary incentive	103				12.88 (3)	0.005	9.47
Intercept		18.41±19.84	[-20.47;57.28]	—			
Beta (Gender)		0.77±0.31	[0.17;1.37]	0.012			
Beta (Monetary incentive)		14.4±4.48	[5.61;23.19]	0.001			
Interaction		-0.21±0.07	[-0.34;-0.07]	0.003			
Ethnicity * Prompt frequency	116				8.55 (3)	0.036	4.72
Intercept		75.67±7.65	[60.69;90.66]	—			
Beta (Ethnicity)		0.11±0.16	[-0.21;0.43]	0.501			
Beta (Prompt frequency)		-0.18±5.54	[-11.04;10.68]	0.974			
Interaction		-0.14±0.12	[-0.38;0.09]	0.223			

Characteristic	k	Estimate ± SE	95% CI	*P*-value	Q_M test (*df*)	*P*-value	R^2
Ethnicity * Number of EMA days	116				3.93 (3)	0.269	1.23
Intercept		66.4±8.51	[49.71;83.09]	—			
Beta (Ethnicity)		0.05±0.16	[-0.26;0.35]	0.771			
Beta (Number of EMA days)		3.72±3.33	[-2.81;10.24]	0.264			
Interaction		-0.05±0.06	[-0.16;0.06]	0.392			
Ethnicity * Number of items	45				2.37 (3)	0.499	0.00
Intercept		69.86±11.45	[47.41;92.3]	—			
Beta (Ethnicity)		-0.19±0.32	[-0.81;0.43]	0.545			
Beta (Number of items)		2.12±3.68	[-5.09;9.33]	0.565			
Interaction		0.03±0.1	[-0.17;0.23]	0.747			
Ethnicity * Monetary incentive	74				5.97 (3)	0.113	4.76
Intercept		53.06±12.8	[27.97;78.14]	—			
Beta (Ethnicity)		0.41±0.23	[-0.05;0.87]	0.083			
Beta (Monetary incentive)		5.77±2.96	[-0.02;11.57]	0.051			
Interaction		-0.11±0.05	[-0.22;-0.01]	0.038			
Mean sample age * Prompt frequency	213				7.28 (3)	0.063	1.96
Intercept		114.69±19.18	[77.1;152.27]	—			
Beta (Mean sample age)		-2.65±1.32	[-5.24;-0.07]	0.044			
Beta (Prompt frequency)		-28.35±13.38	[-54.57;-2.12]	0.034			
Interaction		1.72±0.9	[-0.05;3.49]	0.057			
Mean sample age * Number of EMA days	215				3.37 (3)	0.339	0.21
Intercept		96.64±25.69	[46.3;146.99]	—			
Beta (Mean sample age)		-1.42±1.74	[-4.83;2]	0.417			
Beta (Number of EMA days)		-6.1±9.68	[-25.07;12.88]	0.529			
Interaction		0.3±0.66	[-0.99;1.59]	0.651			
Mean sample age * Number of items	100				4.09 (3)	0.251	1.18
Intercept		101.05±34.99	[32.48;169.63]	—			
Beta (Mean sample age)		-1.59±2.43	[-6.34;3.17]	0.513			
Beta (Number of items)		-5.58±12.77	[-30.61;19.45]	0.662			
Interaction		0.21±0.88	[-1.51;1.93]	0.814			
Mean sample age * Monetary incentive	105				3.15 (3)	0.370	0.46
Intercept		108.98±55.02	[1.15;216.81]	—			
Beta (Mean sample age)		-2.92±3.67	[-10.11;4.26]	0.425			
Beta (Monetary incentive)		-6.04±12.23	[-30;17.92]	0.621			
Interaction		0.54±0.81	[-1.04;2.13]	0.500			

Table S5.3. Interaction models predicting retention

Characteristic	k	Estimate ± SE	95% CI	*P*-value	Q_M test (*df*)	*P*-value	R^2
Clinical status * Prompt frequency	79				3.94 (3)	0.268	2.04
Intercept (clinical)	67	1.31±0.06	[1.19;1.44]	—			
Beta (healthy controls)	13	0.18±0.15	[-0.12;0.48]	0.231			
Beta (Prompt frequency)	67	0.05±0.05	[-0.05;0.14]	0.315			
Interaction	13	-0.06±0.1	[-0.26;0.13]	0.524			
Clinical status * Number of EMA	84				12.05 (3)	0.007	12.32
Intercept (clinical)	71	1.57±0.07	[1.44;1.7]	—			
Beta (healthy controls)	14	0.01±0.17	[-0.32;0.33]	0.970			
Beta (Number of EMA days)	71	-0.07±0.02	[-0.12;-0.02]	0.003			
Interaction	14	0.02±0.07	[-0.12;0.16]	0.781			
Gender * Prompt frequency	141				2.20 (3)	0.533	0.00
Intercept		1.39±0.11	[1.17;1.6]	—			
Beta (Gender)		0±0	[0;0]	0.666			
Beta (Prompt frequency)		-0.02±0.06	[-0.15;0.1]	0.716			
Interaction		0±0	[0;0]	0.399			
Gender * Number of EMA days	153				17.85 (3)	0.000	9.80
Intercept		1.37±0.11	[1.16;1.59]	—			
Beta (Gender)		0±0	[0;0.01]	0.134			
Beta (Number of EMA days)		-0.01±0.05	[-0.1;0.08]	0.794			
Interaction		0±0	[0;0]	0.259			
Gender * Number of items	76				5.33 (3)	0.149	1.99
Intercept		1.25±0.19	[0.88;1.63]	—			
Beta (Gender)		0±0	[0;0.01]	0.268			
Beta (Number of items)		0.01±0.07	[-0.12;0.15]	0.847			
Interaction		0±0	[0;0]	0.559			
Gender * Monetary incentive	77				1.18 (3)	0.759	0.00
Intercept		0.98±0.35	[0.3;1.66]	—			
Beta (Gender)		0.01±0.01	[0;0.02]	0.280			
Beta (Monetary incentive)		0.09±0.08	[-0.08;0.25]	0.297			
Interaction		0±0	[0;0]	0.290			
Ethnicity * Prompt frequency	82				3.68 (3)	0.298	1.40
Intercept		1.5±0.11	[1.28;1.72]	—			
Beta (Ethnicity)		0±0	[-0.01;0]	0.174			
Beta (Prompt frequency)		-0.08±0.09	[-0.25;0.1]	0.386			
Interaction		0±0	[0;0.01]	0.365			
Ethnicity * Number of EMA days	88				16.56 (3)	0.001	15.68
Intercept		1.7±0.09	[1.52;1.88]	—			
Beta (Ethnicity)		0±0	[-0.01;0]	0.030			
Beta (Number of EMA days)		-0.11±0.03	[-0.18;-0.04]	0.001			
Interaction		0±0	[0;0]	0.075			

Characteristic	k	Estimate ± SE	95% CI	*P*-value	Q_M test (*df*)	*P*-value	R^2
Ethnicity * Number of items	43				7.89 (3)	0.048	14.22
Intercept		1.63±0.13	[1.38;1.89]	—			
Beta (Ethnicity)		-0.01±0	[-0.01;0]	0.078			
Beta (Number of items)		-0.06±0.05	[-0.15;0.03]	0.171			
Interaction		0±0	[0;0]	0.195			
Ethnicity * Monetary incentive	54				1.83 (3)	0.608	0.00
Intercept		1.29±0.24	[0.82;1.77]	—			
Beta (Ethnicity)		0±0	[-0.01;0.01]	0.583			
Beta (Monetary incentive)		0.03±0.06	[-0.08;0.14]	0.649			
Interaction		0±0	[0;0]	0.407			
Mean sample age * Prompt	145				4.92 (3)	0.178	2.05
Intercept		1.19±0.23	[0.74;1.64]	—			
Beta (Mean sample age)		0.01±0.02	[-0.02;0.04]	0.501			
Beta (Prompt frequency)		0.26±0.16	[-0.06;0.58]	0.114			
Interaction		-0.02±0.01	[-0.04;0.01]	0.153			
Mean sample age * Number of EMA	158				17.50 (3)	0.001	9.61
Intercept		1.58±0.22	[1.14;2.02]	—			
Beta (Mean sample age)		0±0.02	[-0.03;0.03]	0.824			
Beta (Number of EMA days)		-0.04±0.09	[-0.22;0.13]	0.625			
Interaction		0±0.01	[-0.01;0.01]	0.844			
Mean sample age * Number of	79				4.14 (3)	0.246	2.84
Intercept		1.79±0.34	[1.12;2.46]	—			
Beta (Mean sample age)		-0.03±0.03	[-0.08;0.02]	0.316			
Beta (Number of items)		-0.09±0.13	[-0.33;0.16]	0.491			
Interaction		0.01±0.01	[-0.01;0.02]	0.589			
Mean sample age * Monetary	78				0.58 (3)	0.902	0.00
Intercept		1.05±1.02	[-0.95;3.04]	—			
Beta (Mean sample age)		0.02±0.07	[-0.12;0.15]	0.778			
Beta (Monetary incentive)		0.09±0.23	[-0.36;0.55]	0.689			
Interaction		-0.01±0.02	[-0.04;0.02]	0.696			

Note: Parameters are presented on the arcsine scale. Unlisted predictors are dropped due to less than 40 available studies per predictor, or less than 10 per category.

Meta-analyses of within study associations with compliance

Figure S11.1. Funnel plot for gender differences

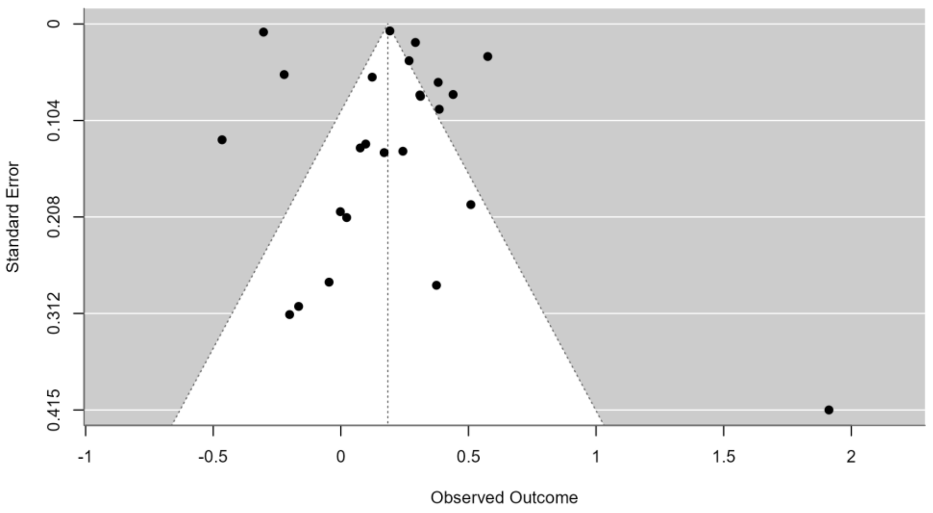

Figure S11.2. Influence plot for gender differences

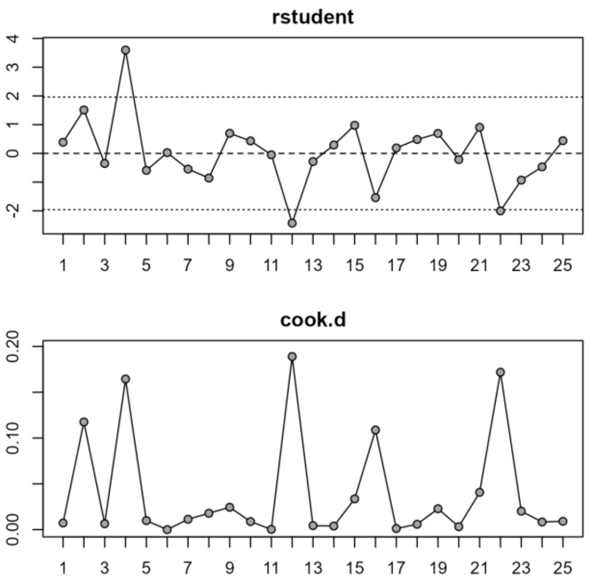

Figure S11.3. Funnel plot for age-compliance correlation

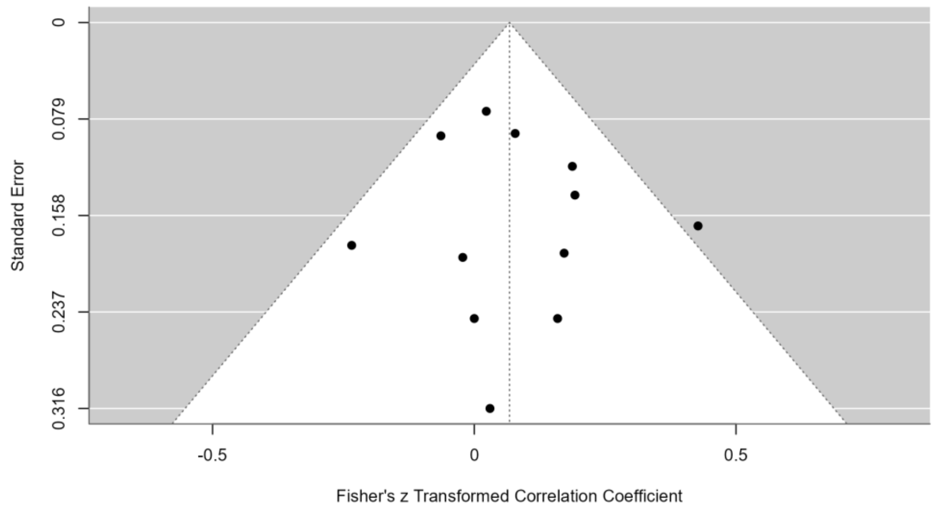

Figure S11.4. Influence plot for age-compliance correlation

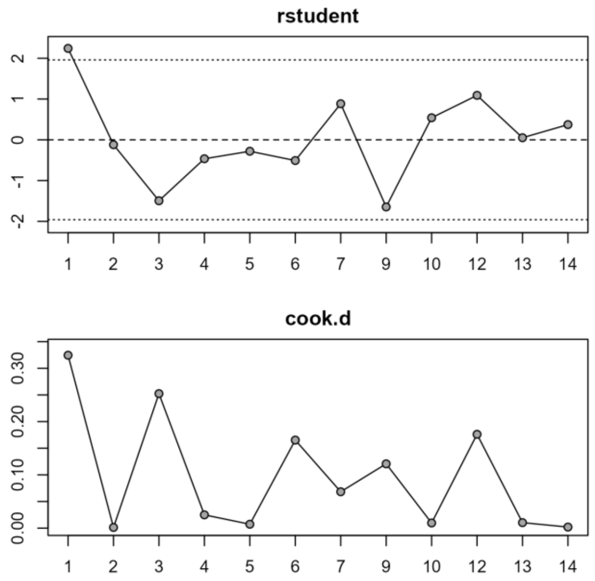

Supplement: Multimedia Appendix 5 [file jmir_v27i1e65710_app5.pdf]
